# HMGA1a Recognition Candidate DNA Sequences in Humans

**DOI:** 10.1371/journal.pone.0008004

**Published:** 2009-11-24

**Authors:** Takayuki Manabe, Taiichi Katayama, Masaya Tohyama

**Affiliations:** 1 Division of Gene Expression Mechanism, Institute for Comprehensive Medical Science, Fujita Health University, Toyoake, Aichi, Japan; 2 Department of Anatomy and Neuroscience, Osaka University Graduate School of Medicine, Suita, Osaka, Japan; 3 Department of Child Development, United Graduate School of Child Development, Osaka University, Kanazawa University, and Hamamatsu University School of Medicine, Suita, Osaka, Japan; Roswell Park Cancer Institute, United States of America

## Abstract

High mobility group protein A1a (HMGA1a) acts as an architectural transcription factor and influences a diverse array of normal biological processes. It binds AT-rich sequences, and previous reports have demonstrated HMGA1a binding to the authentic promoters of various genes. However, the precise sequences that HMGA1a binds to remain to be clarified. Therefore, in this study, we searched for the sequences with the highest affinity for human HMGA1a using an existing SELEX method, and then compared the identified sequences with known human promoter sequences. Based on our results, we propose the sequences “-(G/A)-G-(A/T)-(A/T)-A-T-T-T-” as HMGA1a-binding candidate sequences. Furthermore, these candidate sequences bound native human HMGA1a from SK-N-SH cells. When candidate sequences were analyzed by performing FASTAs against all known human promoter sequences, 500–900 sequences were hit by each one. Some of the extracted genes have already been proven or suggested as HMGA1a-binding promoters. The candidate sequences presented here represent important information for research into the various roles of HMGA1a, including cell differentiation, death, growth, proliferation, and the pathogenesis of cancer.

## Introduction

High mobility group protein A1a (HMGA1a) participates in a wide variety of nuclear processes acting as an architectural transcription factor regulating the expression of numerous genes [1]–[3]. This protein influences a diverse array of normal biological processes, including cell differentiation, death, growth and proliferation, and is involved in the pathogenesis of cancer via protein–protein and DNA–protein interactions [1]–[3]. Therefore, HMGA1a protein has been described as the central ‘hub’ of nuclear function [2].

HMGA1a binds AT-rich sequences via its own AT-hook, and functions in a variety of ways [1]–[3]. Many previous reports have demonstrated HMGA1a binding to the authentic promoters of various genes (for example, human KIT Ligand (hKL) [4], Xeroderma pigmentosum complementation group A [5], Cox2 [6], [7], interferon-β [8], interleukin-10 [9] and -4 [10], iNos/Nos2 [11], c-Fos and SM22α [12]) using DNase I protection assays and/or electrophoretic mobility shift assays (EMSAs). Furthermore, several HMGA1a-regulating genes and pathways have been suggested by microarray analyses [13]. However, although AT-rich sequences exist within authentic gene promoters, their affinity for HMGA1a varies from strong to weak to none at all; even within the same promoter, AT-rich sequences can have vastly differing affinities for HMGA1a [8], [14]. It remains to be clarified exactly which sequences HMGA1a binds to, and whether and how co-factors, structures, and the existence of binding regions on the surface of the DNA-protein complex influence HMGA1a-DNA binding. Therefore, using an existing SELEX method to study all known human promoter sequences, we searched for the sequences with the highest affinity for human HMGA1a.

## Results and Discussion

### Determination of HMGA1a Recognition Candidate DNA Sequences in Humans

**Figure 1:**
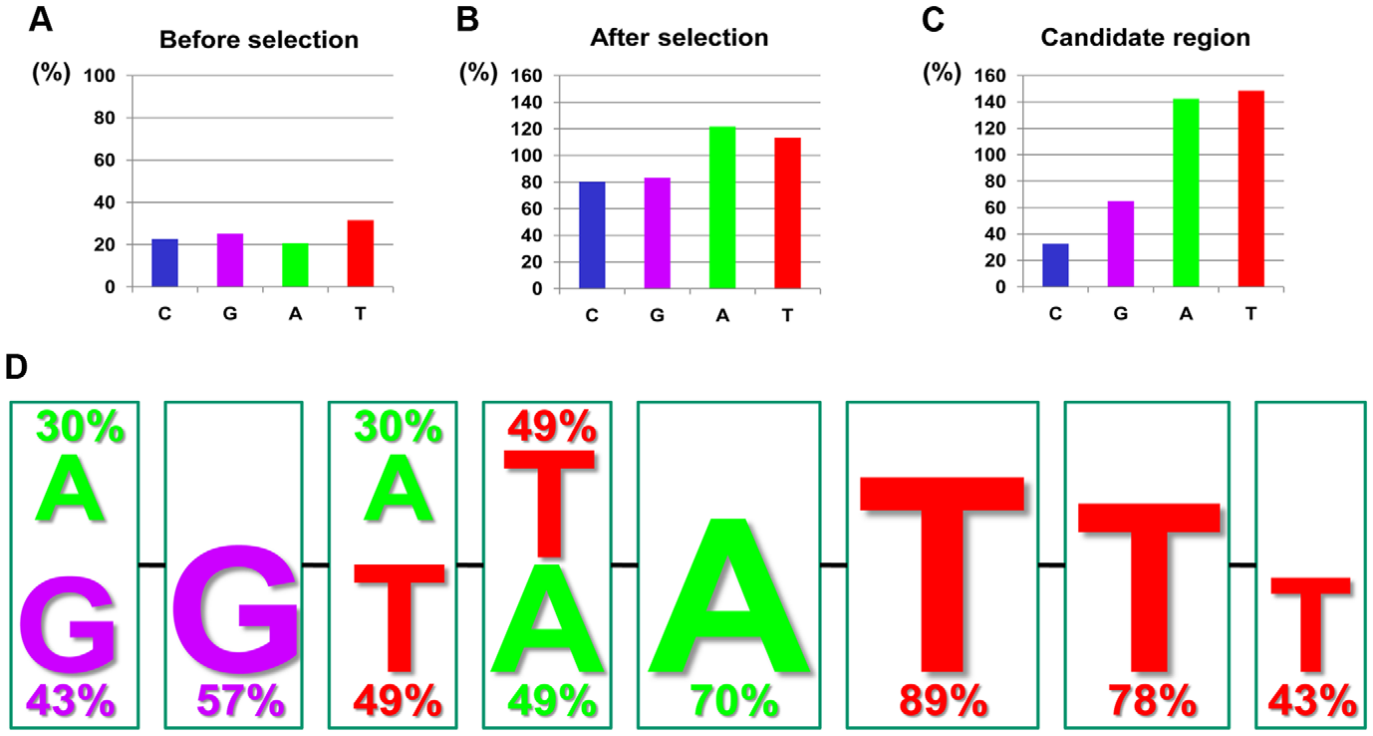
HMGA1a Recognition Candidate DNA Sequences by SELEX. Ratio of bases in synthetic DNA sequences before (**A**) and after (**B**) SELEX assays. (**C**) Ratio of bases in regions of candidate DNA sequences after SELEX assays. (**D**) Candidate HMGA1a binding sequences are shown.

The ratios of the four bases in the synthesized random sequences used in this research, which were placed between T7 sequences, were almost uniform, as a result of a direct sequencing (Figure 1a). When these random sequences of DNA were analyzed using the SELEX method with *E. coli.*-expressed recombinant HMGA1a [15], the ratio of the four bases became AT-rich, with the frequencies of A and T significantly higher (by about 40%) than the frequencies of G and C (Figure 1b). This result shows that the SELEX system selects specific bases; in the case here, and as reported [1], AT-rich sequences. The relative levels of bases in regions assumed to be recognition sequences was as follows: C<G≪<A/T (Figures S1 and 1c). The bases A and T were twice as common, or more, as the bases C and G (Figures S1 and1c). We propose the sequences “-(G/A)-G-(A/T)-(A/T)-A-T-T-T-” as HMGA1a-binding candidate sequences (Figure 1d). Besides being AT-rich, the inclusion of a GG sequence immediately before the AT-rich sequence is interesting. Indeed, the existence of such a GG sequence in authentic promoters has been reported [3], [8], [10].

### The Candidate Sequences Bound Native Human HMGA1a

**Figure 2:**
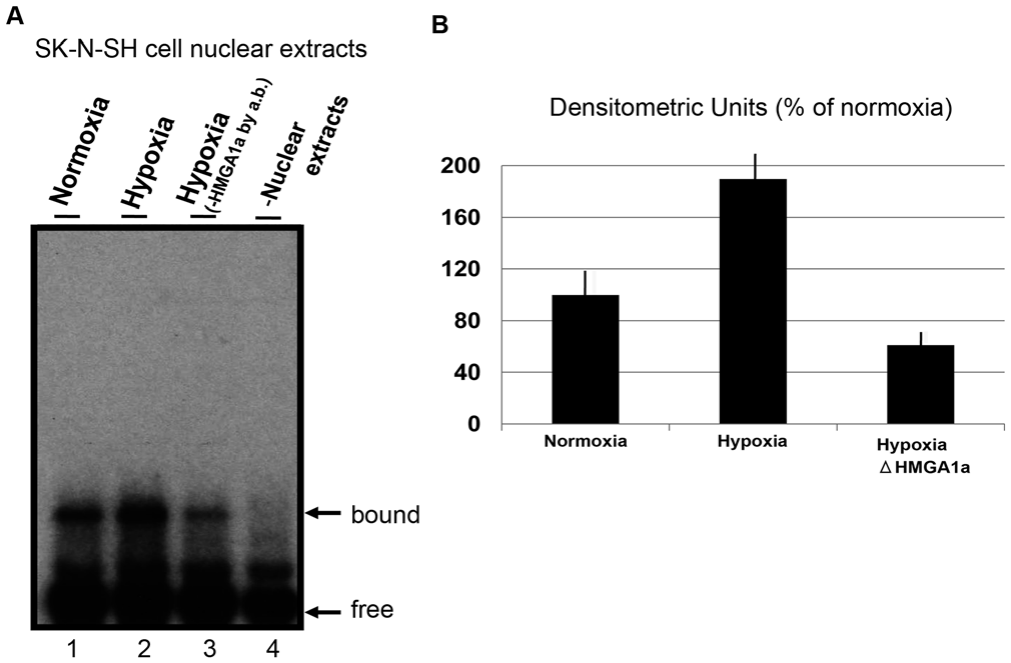
Effects of endogenous HMGA1a on binding to the candidate sequences by EMSA. (**A**) Radiography of EMSA using nuclear extracts obtained from human neuroblastoma SK-N-SH cells under normoxic (lane 1) or hypoxic (lane 2) conditions, or hypoxia (HMGA1a removal: lane 3). (**B**) Densitometric quantitative data from (A) shown as the % of the levels in normoxia.

Native HMGA1a undergoes various post-translational modifications [1], [2]. Therefore, binding of endogenous HMGA1a from human cell nuclear extracts to these candidate sequences was examined by EMSAs (Figures 2A and 2B). Previous reports have demonstrated that HMGA1a expression is significantly increased by hypoxia stimuli in human neuroblastoma SK-N-SH cells, but not in HEK293T or HeLa cells [16], [17]. Using the system described in those reports, binding that was weak under normoxia (Figure 2A, lane 1) became much stronger following hypoxic stimulation (Fig. 2A, lane 2 and Fig. 2B). This increase in binding was prevented by inactivation of HMGA1a in the nuclear extracts using an antibody against it (Figures. 2A and 2B). Therefore, our advocated candidate sequence bound native human HMGA1a from SK-N-SH cells.

### The Candidate Sequences in All Known Human Promoters by FASTA Analysis

There were eight candidate sequences in total: GGAAATTT, GGATATTT, GGTAATTT, GGTTATTT, AGAAATTT, AGATATTT, AGTAATTT, and AGTTATTT. When all known human promoter sequences were analyzed by performing a FASTA on each sequence, 500–900 sequences were hit by each one (Table S1). It is interesting that two or more candidate sequences were found in many of the extracted gene promoters, while the vast majority of human promoter sequences were not hit by any of the candidate sequences (Table 1). This strongly suggests that these candidate sequences are genuine. Moreover, it is also interesting that some of the genes that have already been proven or suggested to have promoters that bind HMGA1a were extracted (Table 2).

**Table 1:** Percentage of repetition in other candidate sequences on the hit gene promoters retrieved using each candidate sequence.

	(a)								
Retrieval candidate sequences	0 (b)	1	2	3	4	5	6	7 (c)	Number of total hits
GGAAATTT	5.8%	13.3%	20.1%	24.6%	22.0%	10.4%	3.4%	0.48%	618
GGTAATTT	4.9%	14.1%	20.4%	23.5%	20.1%	12.3%	4.2%	0.54%	553
GGATATTT	5.9%	13.2%	21.5%	23.2%	19.9%	11.2%	4.5%	0.59%	508
GGTTATTT	6.4%	13.4%	19.5%	23.2%	21.9%	11.4%	3.5%	0.55%	543
AGTTATTT	6.9%	11.9%	24.5%	22.9%	20.0%	10.0%	3.5%	0.38%	781
AGAAATTT	7.0%	15.0%	21.8%	25.1%	18.2%	9.8%	2.8%	0.35%	859
AGATATTT	5.2%	16.6%	20.1%	23.6%	19.7%	11.3%	3.1%	0.39%	767
AGTAATTT	5.4%	12.7%	20.0%	26.6%	19.1%	12.3%	3.5%	0.45%	661

(a): Number of repetition with other candidate sequences. (b): Only the retrieval sequence. (c): All candidate sequences.

**Table 2:** List of genes that have already been proven or suggested to have promoters that bind HMGA1a.

No.	Promoters	(a)	(b)	(c)	(d)	(e)	(f)	(g)	(h)	Ref.
EP64001	rag-1	+	−	+	+	+	+	+	+	[19]
EP07113	interferon -gamma (IFNγ)	+	+	−	−	+	+	+	−	[18]
EP16050	HMG-CoA reductase	+	−	+	+	−	+	+	−	[13]
EP11141	estrogen receptor	+	+	+	−	+	+	−	−	[13]
EP73494	CCNB2 (coding for the cyclin B2 protein)	−	−	+	−	+	+	+	+	[23]
EP11104	β-globin	−	+	+	−	+	+	+	−	[26]
EP07112	interferon -beta (IFNβ)	+	−	−	−	+	+	+	−	[8]
EP73108	CD44	−	−	+	+	+	+	−	−	[22]
EP07121	MHCII HLA-DRA	−	+	+	−	+	−	−	+	[27]
EP11145	FOS	+	−	+	−	−	−	+	−	[12]
EP47012	Inducible Nitric Oxide Synthase (iNOS)	+	+	−	−	−	−	−	+	[11]
EP59011	elk-1	−	−	−	+	−	−	+	+	[13]
EP07114	interleukin-2 (IL-2)	−	+	+	−	−	−	−	−	[20]
EP15045	ErbB2 (HER2/neu)	+	−	−	−	+	−	−	−	[25]
EP15046	ErbB2 (HER2/neu)	+	−	−	−	+	−	−	−	[25]
EP25083	rhodopsin	−	−	+	+	−	−	−	−	[21]
EP73053	crystallin, alpha B (CRYAB)	−	+	−	−	−	−	−	−	[24]
EP26038	interleukin-4 (IL-4)	−	−	−	−	−	−	+	−	[10]

(a): GGAAATTT, (b): GGTAATTT, (c): GGATATTT, (d): GGTTATTT, (e): AGTTATTT, (f): AGAAATTT, (g): AGATATTT, (h): AGTAATTT.

The functions of HMGA1a are diverse and it is known to have a role in disease appearance; thus, the possibility of its becoming a target of treatments has been suggested ([28], Table 3). That is, the candidate sequences proposed by this study may be a blocker of the transcription of cancer-related genes (Table 3), as decoy DNAs. We also reported that a decoy RNA of a specific HMGA1a-binding sequence prevents cell death [29]. In conclusion, the candidate sequences presented here represent important information for research into the various roles of HMGA1a.

**Table 3:** List of genes that have already been proven to associate with each HMGA1a-related cancer/tumor.

HMGA1a-related tumor/cancer (organs) (a)	Each tumor/cancer-associated gene promoter	(b)	(c)	(d)	(e)	(f)	(g)	(h)	(i)	Ref.
Pancreas	EP73094: histone deacetylase 1 (HDAC1)	−	+	−	−	+	+	−	−	[30]
	EP14063: interleukin-1 alpha (IL-1α)	−	+	+	+	+	+	+	+	[31]
	EP11158: tumor necrosis factor alpha (TNFα)	−	+	−	−	−	+	−	−	[32]
Pituitary	EP73927: tumor-transforming gene-1 (PTTG1)	−	−	+	−	+	+	+	+	[33]
	EP73494: CCNB2 (coding for the cyclin B2 protein)	−	−	+	−	+	+	+	+	[23]
Thyroid	EP74305: glutathione peroxidase 3 (GPX3)	−	−	−	−	−	−	+	+	[34]
Thyroid and Breast	EP74327: melanoma antigen gene A3 (MAGE-A3)	−	+	−	−	+	+	+	+	[35], [36]
Breast	EP73942: tumor susceptibility gene 101 (TSG101)	+	−	+	−	+	+	+	+	[37]
	EP11141: estrogen receptor	+	+	+	−	+	+	−	−	[38]
	EP15045, EP15046: ErbB2 (HER2/neu)	+	−	−	−	+	−	−	−	[39]
	EP73108: CD44	−	−	+	+	+	+	−	−	[40]
	EP73128: a disintegrin and metalloproteinase 15 (ADAM15)	−	−	−	−	+	−	−	−	[41]
	EP17080, 17081: interleukin-1 (IL-6)	−	−	−	−	+	−	+	+	[42]
Uterine	EP74172: heterogeneous nuclear ribonucleoprotein A1 (hnRNP A1)	+	+	−	+	−	+	+	−	[43]
	EP74085: hnRNP C	+	−	−	+	+	+	−	−	[43]
	EP74526: proliferating cell nuclear antigen (PCNA)	−	+	−	−	−	+	+	+	[43]
Glioblast	EP73956: melanoma antigen gene E1 (MAGE-E1)	+	−	−	+	−	+	+	−	[44]
Pancreas, Pituitary, Thyroid and Breast	EP15043: epidermal growth factor (EGFR)(ErbB1/HER1)	−	−	−	−	−	+	−	−	[45]–[47]
	EP15044: EGFR (ErbB1/HER1)	−	−	−	+	+	+	−	+	[45]–[47]
	EP73733: CCND3 (coding for the cyclin D3 protein)	−	−	−	−	+	−	−	+	[48]–[51]
	EP73959, EP73960: hnRNP K	−	−	+	+	−	+	−	+	[52]–[55]

(a): Reviewed in Ref. 1, (b): GGAAATTT, (c): GGTAATTT, (d): GGATATTT, (e): GGTTATTT, (f): AGTTATTT, (g): AGAAATTT, (h): AGATATTT, (i): AGTAATTT.

## Materials and Methods

### DNA Selection Assay In Vitro (SELEX)

A synthesized DNA (1 pmol) [5′-GGTGATCAGATTCTGATCCA (N_31_) TGAAGCTTGGATCCGTCGC-3′] molecule containing a 31-nucleotide random sequence (20.7% A, 22.7% C, 31.5% T, 25.1% G by direct sequencing of 16 clones) was amplified (seven cycles) by PCR, followed by incubation with *E. coli.*-expressed rHMGA1a in incubation buffer [16] for 30 min at 25°C. The reaction solution was then subjected to immunoprecipitation with an antibody against HMGA1, followed by amplification (seven cycles) by PCR. The PCR products were cloned into a pGEM-T vector and analyzed by direct sequencing.

### Gel electrophoresis Mobility Shift Assay (EMSA)

After determining the protein content in the nuclear extracts, an aliquot containing 5 µg of protein was incubated with 1 µg of poly-dIdC in incubation buffer; then, 1 µg of ^32^P-labeled-DNA probe (gcg-G/A-G-T/A-A/T-ATTTcgc) was added in a total volume of 50 µl, and the incubation was allowed to continue for another 30 min at 25°C. Bound and free probes were separated by 4% polyacrylamide gel electrophoresis in buffer (pH 8.5) containing 50 mM Tris, 0.38 M glycine and 2 mM EDTA at a constant voltage of 11 V/cm for 1.5 h at 4°C. Dried gels were analyzed by autoradiography.

## Supporting Information

Figure S1 Direct sequencing data after SELEX assay.
(3.58 MB TIF)

Table S1 Hit gene promoters of each candidate gene.
(0.32 MB PDF)
