# Supplementary figures and images for: HMGA1a Recognition Candidate DNA Sequences in Humans
Source: PLoS One. 2009 Nov 24;4(11):e8004. doi: 10.1371/journal.pone.0008004 (PMC2777381; doi:10.1371/journal.pone.0008004)

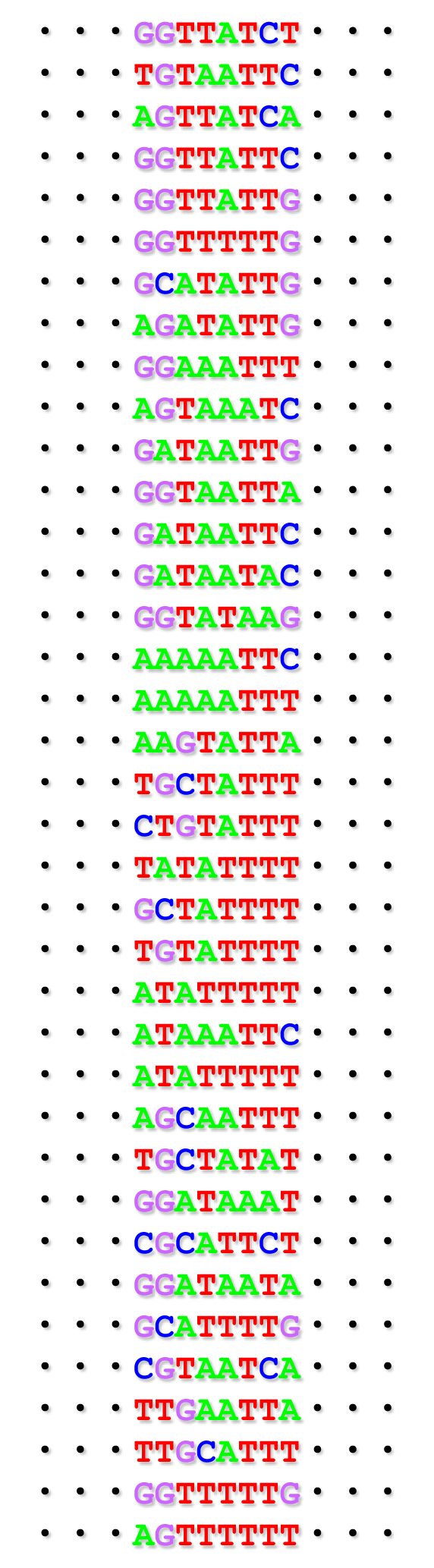

Supplement: Figure S1 — Direct sequencing data after SELEX assay. (3.58 MB TIF) [file pone.0008004.s001.tif]
