# Supplementary material for: HMGA1a Recognition Candidate DNA Sequences in Humans
Source: PLoS One. 2009 Nov 24;4(11):e8004. doi: 10.1371/journal.pone.0008004 (PMC2777381; doi:10.1371/journal.pone.0008004)
Supplement: Table S1 — Hit gene promoters of each candidate gene. (0.32 MB PDF) [file pone.0008004.s002.pdf]

Supplemental Table S1

Hit gene promoters of each candidate gene

GGAAATTT

EP74605 (+) Hs PMSCL1 P1
EP74608 (+) Hs MRPL24
EP74606 (+) Hs PMSCL1 P2+
EP74612 (+) Hs PMAIP1
EP74613 (+) Hs CPSF5
EP74616 (+) Hs COIL
EP73942 (+) Hs TSG101
EP14062 (-) Hs IFN-a'4b
EP11134 (-) Hs IFN l'2c1 (-a'17)
EP07113 (+) Hs g'-interferon
EP73950 (+) Hs ABCE1
EP73953 (+) Hs ELAC2
EP73956 (+) Hs MAGEE1
EP41009 (+) Hs ARF 3
EP39004 (+) Hs CSRP1
EP56002 (+) Hs glycophorin A
EP56004 (-) Hs glycophorin E
EP56003 (-) Hs glycophorin B
EP25021 (+) Hs IFIT1
EP73970 (+) Hs CUL1
EP27009 (+) Hs IFI 6-16
EP35004 (-) Hs H19
EP73975 (+) Hs VDAC1
EP74326 (+) Hs FBXO22
EP58019 (+) Hs WT1
EP28011 (+) Hs[GdX]
EP73980 (+) Hs MPHOSPH6
EP64001 (+) Hs rag-1
EP73984 (+) Hs CD37
EP73983 (+) Hs STK16
EP16071 (+) Hs[ERV3]
EP73991 (+) Hs MRPS31
EP73993 (+) Hs HRB2
EP74003 (+) Hs PRPF18
EP74010 (+) Hs SNRPD3
EP74392 (+) Hs RAB6A
EP11141 (+) Hs estrogen receptor
EP73771 (+) Hs SNX6
EP73779 (+) Hs MGC13204
EP74031 (+) Hs GOT2
EP27010 (+) Hs L-myc
EP74035 (+) Hs PPP2R5C
EP73252 (+) Hs TMEM30A
EP74037 (+) Hs AK2
EP74040 (+) Hs VAMP8
EP26030 (+) Hs c-abl 7 kb E1P1
EP73559 (+) Hs CTSL
EP73811 (+) Hs HPCL2
EP74065 (+) Hs PSMB8
EP74067 (+) Hs RPS24
EP74066 (+) Hs MRPS16
EP74070 (+) Hs COX6A1
EP74075 (+) Hs WBSCR1
EP74079 (+) Hs PGAM1 P1
EP74080 (+) Hs PGAM1 P2+
EP74085 (+) Hs HNRPC
EP26001 (+) Hs TCR va' HD-Mar
EP74115 (+) Hs ZNF265
EP74116 (+) Hs SAA4
EP74117 (+) Hs APCS
EP40002 (+) Hs complement C5
EP74134 (+) Hs UMPS
EP74136 (+) Hs TIMM17A
EP73008 (+) Hs PDHA1
EP73009 (+) Hs GJA1
EP74140 (+) Hs DNAJA2
EP73019 (+) Hs SSBP1
EP74151 (+) Hs BCAS2
EP74510 (+) Hs KNG
EP74167 (+) Hs TCFL1
EP74173 (+) Hs ART3
EP74185 (+) Hs FTSJ3
EP73066 (+) Hs MMS19L
EP73067 (+) Hs FLJ13154
EP74200 (+) Hs IGFBP6
EP73071 (+) Hs RPL34
EP73700 (+) Hs PGM1
EP74207 (+) Hs EEF1B2 P2+
EP74206 (+) Hs EEF1B2 P1
EP73082 (+) Hs SPP1
EP73083 (+) Hs IL8
EP73086 (+) Hs STOML2
EP73962 (+) Hs RPL39L
EP73964 (+) Hs TBPL1
EP73968 (+) Hs ING3
EP73722 (+) Hs SEPW1
EP73097 (+) Hs HSPCA
EP73972 (+) Hs HERPUD1
EP74233 (+) Hs ANP32A
EP73106 (+) Hs ZFP36L1
EP73107 (+) Hs IGFBP3
EP74242 (+) Hs MRPL50
EP73113 (+) Hs MKLN1
EP74243 (+) Hs PDCD10
EP74251 (+) Hs GARS P1
EP73996 (+) Hs EHD1
EP74252 (+) Hs GARS P2+
EP45003 (+) Hs endothelin-B rec.
EP73132 (+) Hs IL10RB
EP74273 (+) Hs PMVK
EP74274 (+) Hs KARS
EP73774 (+) Hs MLPH
EP73152 (+) Hs NDUFA9
EP73837 (+) Hs IDI1
EP74288 (+) Hs CKN1
EP74293 (+) Hs KRT6A
EP74295 (+) Hs CDC2
EP74386 (+) Hs CLDN4
EP11070 (+) Hs histone H2B
EP74300 (+) Hs GABARAPL1
EP74302 (+) Hs TUBB P1
EP74303 (+) Hs TUBB P2+
EP73798 (+) Hs CIR
EP73866 (+) Hs SLC25A19
EP73182 (+) Hs RPLP1
EP37011 (-) Hs MT-IG
EP11073 (+) Hs histone H3b
EP74325 (+) Hs DUT
EP74074 (+) Hs IFNGR1
EP73196 (+) Hs EBNA1BP2
EP74336 (+) Hs HLA DRB1
EP73299 (+) Hs ACTR3
EP74340 (+) Hs GUK1
EP74342 (+) Hs FXYD6
EP74341 (+) Hs UP
EP74346 (+) Hs HLA-E
EP73219 (+) Hs RPL36A
EP36018 (-) Hs nucleolin
EP74359 (+) Hs RBM4
EP73228 (+) Hs CCT2
EP73251 (+) Hs C20ORF31
EP73253 (+) Hs FLJ11305
EP74491 (+) Hs ATP5O
EP73266 (+) Hs MBNL2

EP74144 (+) Hs RNF5
EP74146 (+) Hs NIF3L1
EP73023 (+) Hs MGC2668
EP74408 (+) Hs TAF9
EP74415 (+) Hs HTATIP
EP25034 (+) Hs a'1(I) collagen
EP74165 (+) Hs KRT8
EP74431 (+) Hs SUCLG1
EP73064 (+) Hs OCIA
EP15029 (+) Hs b'-fibrinogen
EP73323 (+) Hs LOC51185
EP74460 (+) Hs TCTE1L
EP74465 (+) Hs DAZAP2
EP73770 (+) Hs SSB
EP74474 (+) Hs ADSL
EP73343 (+) Hs TM4SF13
EP74476 (+) Hs STIP1
EP14059 (-) Hs IFN-a'6
EP74484 (+) Hs TNFAIP1
EP74489 (+) Hs ATP1A1
EP74492 (+) Hs ATP1B3
EP26029 (+) Hs CRP
EP16046 (-) Hs a'-fetoprotein
EP73368 (+) Hs HSPCB
EP73367 (+) Hs ATF6
EP73370 (+) Hs GTF3C5
EP17083 (+) Hs G-CSF
EP74509 (+) Hs FIBP
EP37006 (-) Hs CSF-1
EP74512 (+) Hs ERCC3
EP74511 (+) Hs HARS
EP73379 (+) Hs SPON2
EP73134 (+) Hs FABP5
EP73197 (+) Hs MCP
EP74275 (+) Hs CKAP1
EP74427 (+) Hs MRPS6
EP73396 (+) Hs RIPK3
EP74536 (+) Hs GCA
EP74537 (+) Hs POLR2C
EP73839 (+) Hs HNRPA3
EP73405 (+) Hs PWP1
EP74543 (+) Hs NDUFS5
EP57008 (+) Hs FBP
EP74558 (+) Hs SDCBP
EP73181 (+) Hs FXR1
EP73434 (+) Hs CTGF
EP74123 (+) Hs NDUFB10
EP74575 (+) Hs CCT6A P2+
EP74577 (+) Hs TYMS P1
EP73445 (+) Hs TUBB4
EP11110 (-) Hs e'-globin
EP35012 (+) Hs P-glycoprotein 1
EP73205 (+) Hs LPXN
EP11111 (+) Hs haptoglob Hp1F
EP73581 (+) Hs DNAJA1
EP73479 (+) Hs CEPT1
EP30054 (+) Hs arginase liver
EP30056 (+) Hs OAT
EP74622 (+) Hs PDHB
EP73490 (+) Hs GBP2
EP74629 (+) Hs PFKP
EP73520 (+) Hs CFL1
EP27011 (+) Hs TPI1
EP73332 (+) Hs HPGD
EP73424 (+) Hs PROL3
EP73280 (+) Hs FLJ10276
EP73873 (+) Hs FAM14A
EP40003 (+) Hs DAF
EP73535 (+) Hs TTC4
EP73542 (+) Hs TXNL
EP73354 (+) Hs KIAA0971
EP32001 (+) Hs protein C
EP07095 (+) Hs factor IX
EP73897 (+) Hs DDX39
EP74439 (+) Hs UQCRH
EP73561 (+) Hs CLK1
EP28005 (+) Hs pepsinogen C
EP73315 (+) Hs OVCOV1
EP73568 (+) Hs CITED1
EP74452 (+) Hs TBCC
EP30065 (+) Hs AMY1
EP73331 (+) Hs HSPC251
EP73922 (+) Hs COG4
EP35073 (-) Hs P450 XVIIA1 CYP17
EP73591 (+) Hs EIF3S2
EP73932 (+) Hs NINJ1
EP73690 (+) Hs UBE2N
EP74481 (+) Hs MRPL49
EP68001 (-) Hs CA2
EP74578 (+) Hs TYMS P2+
EP73695 (+) Hs NME2
EP73603 (+) Hs P4HA2
EP73410 (+) Hs OSF
EP07107 (+) Hs preproenkephalin
EP73610 (+) Hs M6PR
EP30039 (-) Hs CRF
EP24001 (+) Hs GRF
EP73623 (+) Hs TARS
EP73727 (+) Hs SNX1
EP07106 (-) Hs CG/LH/FSH/TSH a'
EP30074 (-) Hs FSH b'
EP73112 (+) Hs NONO
EP74532 (+) Hs CCT4
EP14056 (+) Hs prolactin
EP73118 (+) Hs ADH5
EP73749 (+) Hs NDUFA4
EP73659 (+) Hs ENO3
EP14057 (+) Hs thyroglobulin
EP74549 (+) Hs NTS
EP74357 (+) Hs MRPL37
EP17072 (-) Hs IGF II E3P3
EP74556 (+) Hs UBL1
EP73423 (+) Hs CCT8
EP74366 (+) Hs SYAP1
EP74369 (+) Hs VRK1
EP73682 (+) Hs CEACAM1
EP73149 (+) Hs RAB1A
EP14058 (-) Hs IFN-a'13
EP73805 (+) Hs SMT3H2
EP11135 (-) Hs IFN l'2h (-a'14)
EP14060 (-) Hs IFN-a'5
EP74033 (+) Hs HADH2
EP73921 (+) Hs ATP5L
EP74583 (+) Hs TNFSF10
EP74042 (+) Hs SNRPA P1
EP17079 (+) Hs IL1B
EP74589 (+) Hs CLNS1A
EP73171 (+) Hs LUC7A
EP73706 (+) Hs BPGM
EP73708 (+) Hs ACTA2
EP57002 (-) Hs haptoglob HpR
EP71003 (+) Hs RGS1
EP73538 (+) Hs USP11
EP74604 (+) Hs HLA-DPA1
EP73726 (+) Hs SNAP25
EP11158 (+) Hs TNF-a'
EP73876 (+) Hs RAE1
EP17089 (+) Hs HSPA8
EP11159 (+) Hs TNF-b'
EP73732 (+) Hs CAPG
EP73318 (+) Hs ADIPOR1
EP15046 (+) Hs c-erbB2/neu P2+
EP24025 (-) Hs a'2(I) collagen
EP74084 (+) Hs UFD1L
EP73550 (+) Hs EI24
EP73208 (+) Hs RPL32
EP73644 (+) Hs COPEB
EP16068 (+) Hs MHCII HLA DQ2b'
EP60012 (+) Hs a'-1a-AdrenR
EP25039 (-) Hs b'2-AR

EP73502 (+) Hs TOM1
EP73755 (+) Hs PLEK
EP16050 (+) Hs HMG-CoA red.
EP73125 (+) Hs T1A
EP48005 (+) Hs VLDL rec.
EP73765 (+) Hs PKIA
EP73235 (+) Hs SLC31A2
EP73236 (+) Hs CDCA8
EP73238 (+) Hs FLJ10525
EP11146 (+) Hs c-myc P1
EP11148 (+) Hs c-myc P2+
EP73242 (+) Hs FLJ11160
EP73584 (+) Hs DCTD
EP73020 (+) Hs TOSO
EP73534 (+) Hs CTSC
EP74129 (+) Hs CHEK1
EP73254 (+) Hs HT007
EP73540 (+) Hs CD83
EP73796 (+) Hs SRP54
EP26032 (+) Hs c-abl 6 kb E2P3
EP73017 (+) Hs UNRIP
EP73894 (+) Hs TRAP1
EP73804 (+) Hs CCT6B
EP73707 (+) Hs C1S
EP73273 (+) Hs SC5DL
EP73807 (+) Hs GSN
EP73031 (+) Hs C14ORF2
EP73285 (+) Hs COX7A2
EP73820 (+) Hs CKLFSF6
EP73290 (+) Hs LOC51705
EP73061 (+) Hs ITM2B
EP73629 (+) Hs TAF15
EP25050 (+) Hs surfactant p. 5K
EP73185 (+) Hs CLU
EP73835 (+) Hs PECI
EP73302 (+) Hs PRDX4
EP73397 (+) Hs RNP24
EP07117 (+) Hs Ig k' HK101
EP73652 (+) Hs GDI2
EP73851 (+) Hs ATP5E
EP74289 (+) Hs SEPP1
EP73864 (+) Hs CYP3A5
EP73852 (+) Hs ATP6V1D
EP74541 (+) Hs POLR2J
EP73854 (+) Hs HSPC177
EP73320 (+) Hs SBDS
EP73859 (+) Hs LPL
EP73073 (+) Hs SID6
EP14076 (+) Hs CD74
EP73735 (+) Hs GLUL
EP73965 (+) Hs RNF7
EP73146 (+) Hs ATP5J
EP73007 (+) Hs IF
EP73352 (+) Hs C14ORF92
EP73785 (+) Hs MKI67IP
EP73699 (+) Hs PFN2
EP26031 (-) Hs c-abl 7 kb E1P2
EP73361 (+) Hs RER1
EP73364 (+) Hs CORO1A
EP73119 (+) Hs TF
EP73371 (+) Hs CHORDC1
EP73717 (+) Hs PSMC5
EP73372 (+) Hs HBP1
EP73720 (+) Hs RNF4
EP73037 (+) Hs HBXIP
EP73039 (+) Hs DDX18
EP73042 (+) Hs ADH1B
EP73385 (+) Hs CSNK2A1
EP73870 (+) Hs DKFZP586F1524
EP73140 (+) Hs PFDN5
EP73741 (+) Hs HTN1
EP74172 (+) Hs HNRPA1
EP74433 (+) Hs GNB3
EP73399 (+) Hs TMP21
EP73287 (+) Hs ATP6V1H
EP74287 (+) Hs NACA
EP73063 (+) Hs ABP1
EP73941 (+) Hs ERH
EP73408 (+) Hs BTG1
EP74174 (+) Hs PSMA4
EP07111 (+) Hs LeIF-J (IFN-a'7)
EP73210 (+) Hs SLC31A1
EP07112 (-) Hs b'-interferon
EP73301 (+) Hs AF1Q
EP73164 (+) Hs C9ORF19
EP74298 (+) Hs WBP11
EP73920 (+) Hs MRPS22
EP73704 (+) Hs PRG1
EP73075 (+) Hs DUSP11
EP73762 (+) Hs C3ORF4
EP73206 (+) Hs FLJ22965
EP73709 (+) Hs FKBP3
EP73958 (+) Hs TIMM8A
EP74594 (+) Hs CTSL2
EP73827 (+) Hs DC6
EP25044 (-) Hs IL-5 (EDF/TRF)
EP73715 (+) Hs PSMB7
EP74083 (+) Hs LAMR1
EP74464 (+) Hs HSPA5
EP74601 (+) Hs PGRMC1
EP73334 (+) Hs CD14
EP73721 (+) Hs SAT
EP73723 (+) Hs SFRS3
EP73439 (+) Hs NNMT
EP73438 (+) Hs NFE2L2
EP73101 (+) Hs NP
EP31007 (+) Hs HMG-14
EP73110 (+) Hs PPGB
EP73737 (+) Hs H2AFZ
EP73548 (+) Hs ITM2A
EP73353 (+) Hs STAF65
EP73344 (+) Hs ASML3B
EP74043 (+) Hs SNRPA P2+
EP47002 (+) Hs a'_2 integrin
EP73461 (+) Hs ARPC2
EP73121 (+) Hs ACTG2
EP74547 (+) Hs HMGB2
EP41008 (+) Hs snRNP E
EP73471 (+) Hs FRSB
EP73759 (+) Hs NME1
EP74212 (+) Hs SNX17
EP73475 (+) Hs SGK
EP74012 (+) Hs DAP3
EP11145 (+) Hs FOS
EP73769 (+) Hs SEPT6
EP68002 (+) Hs ribosomal p. S19
EP73888 (+) Hs C20ORF43
EP73241 (+) Hs HIMAP4
EP73150 (+) Hs COX7A2L
EP73155 (+) Hs PSMF1
EP73258 (+) Hs RPL37A
EP74432 (+) Hs MOCS2
EP73620 (+) Hs RCN1
EP73165 (+) Hs RAB18
EP74388 (+) Hs MAT2B
EP73308 (+) Hs FKBP1A
EP74046 (+) Hs UQCRC2
EP73262 (+) Hs G3BP
EP74047 (+) Hs RAC1
EP73517 (+) Hs HADHSC
EP74401 (+) Hs MRPS23
EP74208 (+) Hs NFKBIL1
EP73296 (+) Hs DDX49
EP74521 (+) Hs JUN
EP74455 (+) Hs RNPS1
EP30058 (-) Hs MT-IE
EP74153 (+) Hs SKP2
EP74163 (+) Hs NCBP2
EP74413 (+) Hs PCBP2
EP73465 (+) Hs LHFP

EP17067 (+) Hs glucagon
EP74573 (+) Hs DAD1
EP73293 (+) Hs POLE3
EP74426 (+) Hs VPS29
EP73321 (+) Hs LOC51125
EP73647 (+) Hs CPA3
EP73600 (+) Hs VASP
EP74124 (+) Hs VBP1
EP73214 (+) Hs LOC57019
EP73117 (+) Hs AKR1C3
EP73557 (+) Hs HRMT1L1
EP73791 (+) Hs ZNF207
EP73220 (+) Hs TRAF2
EP73269 (+) Hs QPCT
EP73221 (+) Hs PSA1
EP73661 (+) Hs PRSS1
EP73223 (+) Hs SQRDL
EP74026 (+) Hs KYNU
EP11087 (+) Hs g'-fibrinogen
EP73692 (+) Hs LGALS3
EP73328 (+) Hs HSPC148
EP73336 (+) Hs IMAGE145052
EP74320 (+) Hs TM4SF1 P2+
EP74389 (+) Hs BAT1
EP73926 (+) Hs CLDN5
EP73145 (+) Hs ST13
EP73979 (+) Hs MRPL43
EP73346 (+) Hs DDX25
EP74127 (+) Hs COPS5
EP73389 (+) Hs RPL41

EP73825 (+) Hs FLJ10374
EP73256 (+) Hs HMT
EP73255 (+) Hs BAP29
EP74548 (+) Hs DARS
EP74087 (+) Hs GOLPH3
EP73291 (+) Hs LOC51706
EP73393 (+) Hs NDUFV1
EP74493 (+) Hs RPS3A
EP74442 (+) Hs RPL15
EP73627 (+) Hs PTP4A1
EP74597 (+) Hs ARF6
EP73324 (+) Hs VRK3
EP73482 (+) Hs CSE1L
EP73904 (+) Hs RPL18
EP73028 (+) Hs FLJ14800
EP73421 (+) Hs USP16
EP73879 (+) Hs EIF2S1
EP73910 (+) Hs CLTA
EP74164 (+) Hs COX11
EP73822 (+) Hs PLEKHB2
EP11068 (+) Hs histone H2A
EP74520 (+) Hs TCFL4
EP73586 (+) Hs APOL1
EP74505 (+) Hs HMGN3
EP73853 (+) Hs CBX3
EP73377 (+) Hs ARFIP2
EP73303 (+) Hs AUP1
EP73283 (+) Hs ALDH1A1
EP73173 (+) Hs SAP18
EP74361 (+) Hs IKBKG

EP74282 (+) Hs HMG20B
EP73653 (+) Hs GOT1
EP48003 (+) Hs collagenaseIV 72K
EP74193 (+) Hs APG12L
EP73912 (+) Hs CSRP2
EP11098 (-) Hs z'-globin
EP73967 (+) Hs NOLA3
EP73719 (+) Hs RGS2
EP73556 (+) Hs HIF1A
EP73560 (+) Hs B2M
EP73832 (+) Hs GM2A
EP73378 (+) Hs SF3B1
EP17030 (-) Hs snRNA U1 (pU1-6)
EP17096 (+) Hs CD3 (T3) g'
EP73496 (+) Hs VAMP3
EP73574 (+) Hs PET112L
EP73348 (+) Hs HSPC051
EP73384 (+) Hs CA12
EP73435 (+) Hs MDH1
EP74356 (+) Hs BAG1
EP24034 (-) Hs P450 IA1 +MC
EP73687 (+) Hs UBE2D1
EP73365 (+) Hs D1S155E
EP73294 (+) Hs SLC38A2
EP40004 (+) Hs C4BP b' A19 mRNA
EP73018 (+) Hs NK4
EP73444 (+) Hs TSC22
EP73541 (+) Hs TRIP15
EP73923 (+) Hs SYNJ2BP
EP73702 (+) Hs POU2F1

EP73701 (+) Hs PIP
EP74143 (+) Hs TUBG1
EP74390 (+) Hs GGPS1
EP73357 (+) Hs MGP
EP73799 (+) Hs CNIH
EP28009 (-) Hs IGF II E4P4
EP73503 (+) Hs LDHA
EP73366 (+) Hs PARK7
EP74319 (+) Hs TM4SF1 P1
EP74299 (+) Hs SELENBP1
EP74248 (+) Hs HPD
EP74574 (+) Hs CCT6A P1
EP73918 (+) Hs ATP6V0D1
EP73174 (+) Hs PRNP
EP74445 (+) Hs CLPP
EP73326 (+) Hs UBAP1
EP73130 (+) Hs CRACC
EP73477 (+) Hs TAF11
EP74414 (+) Hs MAP2K1IP1
EP73407 (+) Hs ARHGDIB
EP73831 (+) Hs GLA
EP74324 (+) Hs GNG5
EP11121 (+) Hs P450 IA1 +TCDD
EP73637 (+) Hs CRISP2
EP73841 (+) Hs NAPA
EP73592 (+) Hs EIF3S1
EP73624 (+) Hs TCF12
EP47012 (+) Hs[NOS2] iNOS
EP73913 (+) Hs DFFA
EP73899 (+) Hs HSBP1

EP73506 (+) Hs EIF4A1
EP73664 (+) Hs PSMA2
EP73533 (+) Hs ANXA1
EP07105 (-) Hs POMC (ACTH,b'LPH)
EP74349 (+) Hs TPMT
EP74581 (+) Hs GNG11
EP73665 (+) Hs PTMA
EP73536 (+) Hs UCHL1
EP74514 (+) Hs TINF2
EP73310 (+) Hs COX7B
EP74032 (+) Hs MSN
EP73543 (+) Hs TM9SF2
EP73792 (+) Hs SPOP
EP73485 (+) Hs BNIP3L
EP73582 (+) Hs EIF3S6
EP74071 (+) Hs RARS
EP30003 (+) Hs c-N-ras
EP73448 (+) Hs AGR2
EP73611 (+) Hs MAD2L1
EP73076 (+) Hs LOC81501
EP73917 (+) Hs CLPTM1
EP28010 (+) Hs IGF II E2P2
EP73826 (+) Hs ACTR10
EP15045 (+) Hs c-erbB2/neu P1
EP73298 (+) Hs ACAT2
EP74506 (+) Hs PSMD10
EP74352 (+) Hs SIAT7D
EP73509 (+) Hs CTSB
EP16042 (+) Hs ALB
EP73752 (+) Hs NDUFS4

EP74468 (+) Hs HSD17B4

EP74599 (+) Hs CD79B

EP73263 (+) Hs JWA

EP73957 (+) Hs APOH

EP73022 (+) Hs CPSF3

EP73337 (+) Hs RRP4

EP74142 (+) Hs RFX4

EP74104 (+) Hs ACPP

EP74281 (+) Hs PLDN

EP73188 (+) Hs SERPINH1

EP73454 (+) Hs CCT7

EP74586 (+) Hs SFRS8

EP74394 (+) Hs TNRC5

EP74076 (+) Hs RPS4Y

EP74387 (+) Hs TAX1BP1

EP74449 (+) Hs DEAH

EP73814 (+) Hs KIAA0174

EP73179 (+) Hs FLJ13868

EP73872 (+) Hs DC50

EP74591 (+) Hs B3GNT6

EP74106 (+) Hs PAH

EP73159 (+) Hs FABP4

EP73440 (+) Hs PPIE

EP73780 (+) Hs MGC4251

EP11105 (-) Hs d'-globin

EP73673 (+) Hs SPARC

EP73626 (+) Hs ZNF134

EP73764 (+) Hs RAD18

EP73995 (+) Hs EIF1AY

EP73006 (+) Hs CAV1

EP73678 (+) Hs THBS1

EP74475 (+) Hs UROS

EP74409 (+) Hs PSMC2

GGTAATTT

EP73928 (+) Hs RBM14

EP74604 (+) Hs HLA-DPA1

EP74612 (+) Hs PMAIP1

EP73698 (+) Hs PBP

EP73162 (+) Hs LCN7

EP74624 (+) Hs NUP62

EP07114 (+) Hs IL-2 (TCGF)

EP73171 (+) Hs LUC7A

EP56002 (+) Hs glycophorin A

EP73177 (+) Hs MKKS

EP73962 (+) Hs RPL39L

EP73965 (+) Hs RNF7

EP73719 (+) Hs RGS2

EP25038 (+) Hs IFI 54K

EP73724 (+) Hs SH3BGRL

EP73726 (+) Hs SNAP25

EP73975 (+) Hs VDAC1

EP58019 (+) Hs WT1

EP28011 (+) Hs[GdX]

EP73980 (+) Hs MPHOSPH6

EP73984 (+) Hs CD37

EP73985 (+) Hs CDC23

EP73739 (+) Hs NR4A1

EP73741 (+) Hs HTN1

EP73998 (+) Hs TAF7

EP74001 (+) Hs STK25 P2

EP48005 (+) Hs VLDL rec.

EP11141 (+) Hs estrogen receptor

EP74362 (+) Hs ETV5

EP74363 (+) Hs STX10

EP73765 (+) Hs PKIA

EP74369 (+) Hs VRK1

EP74022 (+) Hs SFRS5

EP74037 (+) Hs AK2

EP73793 (+) Hs RPL14

EP73795 (+) Hs SP100

EP74048 (+) Hs NIPSNAP1

EP73808 (+) Hs IL2RG

EP74409 (+) Hs PSMC2

EP74066 (+) Hs MRPS16

EP73814 (+) Hs KIAA0174

EP73283 (+) Hs ALDH1A1

EP74075 (+) Hs WBSCR1

EP17089 (+) Hs HSPA8

EP73827 (+) Hs DC6

EP74172 (+) Hs HNRPA1

EP74082 (+) Hs NARS

EP74083 (+) Hs LAMR1

EP74087 (+) Hs GOLPH3

EP74093 (+) Hs TDO2

EP74101 (+) Hs RPL24

EP73852 (+) Hs ATP6V1D

EP74112 (+) Hs MRPL45

EP74115 (+) Hs ZNF265

EP74116 (+) Hs SAA4

EP74461 (+) Hs NOLC1

EP74119 (+) Hs HLA DQA1

EP74121 (+) Hs NDUFB2

EP73872 (+) Hs DC50

EP73003 (+) Hs RPS8

EP73885 (+) Hs NOSIP

EP74143 (+) Hs TUBG1

EP73895 (+) Hs RNF13

EP73021 (+) Hs NRBP

EP74149 (+) Hs GSTP1

EP74150 (+) Hs NDUFB4

EP74155 (+) Hs MRPS25

EP74160 (+) Hs YARS

EP74162 (+) Hs CS

EP74169 (+) Hs TCL1A

EP74167 (+) Hs TCFL1

EP74173 (+) Hs ART3

EP73048 (+) Hs B4GALT3

EP74178 (+) Hs RTN3

EP73050 (+) Hs CDC10

EP73397 (+) Hs RNP24

EP73399 (+) Hs TMP21

EP74186 (+) Hs TOR3A

EP74188 (+) Hs NUDC

EP74191 (+) Hs TSTA3 P1

EP74192 (+) Hs TSTA3 P2+

EP73065 (+) Hs MATR3

EP74200 (+) Hs IGFBP6

EP74201 (+) Hs PLAT

EP74202 (+) Hs SCAND1

EP74203 (+) Hs MRPS18B

EP73948 (+) Hs KHDRBS1

EP73509 (+) Hs CTSB

EP73074 (+) Hs DDX5

EP74209 (+) Hs KRT17 P1

EP74210 (+) Hs KRT17 P2+

EP73079 (+) Hs TMEM14B

EP74213 (+) Hs APLP2

EP74214 (+) Hs PSMD13

EP73424 (+) Hs PROL3

EP73088 (+) Hs GNPI

EP11104 (+) Hs b'-globin

EP73093 (+) Hs CASQ2

EP74223 (+) Hs CRSP9

EP74224 (+) Hs RBX1

EP74225 (+) Hs EIF3S4

EP74573 (+) Hs DAD1

EP74574 (+) Hs CCT6A P1

EP74347 (+) Hs MYOZ2 P1

EP73101 (+) Hs NP

EP74232 (+) Hs SURF4

EP73444 (+) Hs TSC22

EP74582 (+) Hs S100B

EP74242 (+) Hs MRPL50

EP73112 (+) Hs NONO

EP74243 (+) Hs PDCD10

EP73995 (+) Hs EIF1AY

EP73121 (+) Hs ACTG2

EP73999 (+) Hs STK25 P1

EP74002 (+) Hs STK25 P3+

EP73130 (+) Hs CRACC

EP73474 (+) Hs SERF2

EP73136 (+) Hs SLC26A3

EP74110 (+) Hs UBE2G2

EP73141 (+) Hs MRPL44

EP74275 (+) Hs CKAP1

EP49001 (+) Hs histone H1t

EP73150 (+) Hs COX7A2L

EP73149 (+) Hs RAB1A

EP73495 (+) Hs SCYE1

EP42001 (+) Hs BCKDHA

EP73497 (+) Hs GSTO1

EP47012 (+) Hs[NOS2] iNOS

EP73500 (+) Hs CCNC

EP74036 (+) Hs IMPA1

EP74295 (+) Hs CDC2

EP73851 (+) Hs ATP5E

EP74300 (+) Hs GABARAPL1

EP73799 (+) Hs CNIH

EP73175 (+) Hs PXMP3

EP74309 (+) Hs RPS15A

EP73523 (+) Hs IDH3A

EP73181 (+) Hs FXR1

EP73525 (+) Hs MEP1A

EP74152 (+) Hs BET1

EP74317 (+) Hs RPLP0 P1

EP11073 (+) Hs histone H3b

EP73188 (+) Hs SERPINH1

EP73191 (+) Hs ATP6V1C1

EP74327 (+) Hs MAGEA3

EP74070 (+) Hs COX6A1

EP73195 (+) Hs SFRS6

EP73823 (+) Hs ESRRBL1

EP31009 (+) Hs HMG-17

EP74090 (+) Hs SLC1A5
EP73211 (+) Hs DHRS6
EP73249 (+) Hs FLJ10509
EP73842 (+) Hs TES
EP74348 (+) Hs MYOZ2 P2+
EP73221 (+) Hs PSA1
EP36018 (-) Hs nucleolin
EP15034 (+) Hs collagenase
EP74474 (+) Hs ADSL
EP07121 (+) Hs MHCII HLA-DRA
EP74108 (+) Hs EIF4EL3
EP30065 (+) Hs AMY1
EP74376 (+) Hs HEXB
EP73246 (+) Hs C6ORF37
EP74382 (+) Hs BZW2 P2+
EP73251 (+) Hs C20ORF31
EP74384 (+) Hs NDUFS3
EP73600 (+) Hs VASP
EP73257 (+) Hs RPL23A
EP74392 (+) Hs RAB6A
EP74396 (+) Hs IMPA2
EP74395 (+) Hs MCM2
EP73892 (+) Hs ZNF9
EP73269 (+) Hs QPCT
EP74405 (+) Hs MEL
EP74406 (+) Hs ERCC1
EP74151 (+) Hs BCAS2
EP74408 (+) Hs TAF9
EP73618 (+) Hs RARRES2
EP74410 (+) Hs APPBP1
EP73284 (+) Hs CDC20
EP74164 (+) Hs COX11
EP25041 (+) Hs a'1(III) collagen
EP74423 (+) Hs DSCR5
EP73385 (+) Hs CSNK2A1
EP30071 (+) Hs TSH b'
EP74184 (+) Hs SSSCA1
EP73312 (+) Hs SDCBP2
EP30043 (-) Hs PTH P1+
EP73317 (+) Hs RPS27L
EP74449 (+) Hs DEAH
EP11087 (+) Hs g'-fibrinogen
EP74454 (+) Hs AARS
EP74456 (+) Hs ILF2
EP73326 (+) Hs UBAP1
EP07095 (+) Hs factor IX
EP73328 (+) Hs HSPC148
EP74460 (+) Hs TCTE1L
EP17072 (-) Hs IGF II E3P3
EP74462 (+) Hs PSME1
EP74465 (+) Hs DAZAP2
EP74469 (+) Hs SPARCL1 P1
EP74470 (+) Hs SPARCL1 P2+
EP74564 (+) Hs HPRT1 P2+
EP35012 (+) Hs P-glycoprotein 10
EP73342 (+) Hs p100
EP73924 (+) Hs HN1
EP74378 (+) Hs CCNH
EP14059 (-) Hs IFN-a'6
EP74483 (+) Hs TMSB4X
EP73351 (+) Hs KIAA0102
EP16044 (-) Hs prealbumin
EP74493 (+) Hs RPS3A
EP74494 (+) Hs MCM7
EP26029 (+) Hs CRP
EP73364 (+) Hs CORO1A
EP73800 (+) Hs PSMD14
EP74502 (+) Hs SNRPG
EP73712 (+) Hs PRPS1
EP74506 (+) Hs PSMD10
EP14047 (+) Hs PRBP
EP74513 (+) Hs PPIG
EP73381 (+) Hs GNPAT
EP73138 (+) Hs B3GAT3
EP74517 (+) Hs DHX38
EP74266 (+) Hs CDK5RAP1
EP74519 (+) Hs EIF3S5
EP74521 (+) Hs JUN
EP74522 (+) Hs IMMT
EP74523 (+) Hs DDX1
EP74524 (+) Hs PSMB5
EP74525 (+) Hs C1QBP
EP74526 (+) Hs PCNA
EP73321 (+) Hs LOC51125
EP73744 (+) Hs KPNB2
EP74535 (+) Hs PSMC6
EP73893 (+) Hs PHGDH
EP74538 (+) Hs YWHAE
EP53001 (-) Hs a'2-globin
EP73407 (+) Hs ARHGDIB
EP73410 (+) Hs OSF
EP73412 (+) Hs POLD2
EP11070 (+) Hs histone H2B
EP74554 (+) Hs HNRPAB
EP73421 (+) Hs USP16
EP74557 (+) Hs DECR1
EP74556 (+) Hs UBL1
EP73920 (+) Hs MRPS22
EP74318 (+) Hs RPLP0 P2+
EP73436 (+) Hs MFAP1
EP73435 (+) Hs MDH1
EP73591 (+) Hs EIF3S2
EP74575 (+) Hs CCT6A P2+
EP73787 (+) Hs PNN
EP73790 (+) Hs PSMD11
EP73448 (+) Hs AGR2
EP11068 (+) Hs histone H2A
EP73259 (+) Hs DAB2
EP74587 (+) Hs TTID
EP73452 (+) Hs DNAJB1
EP74590 (+) Hs WDR1
EP57002 (-) Hs haptoglob HpR
EP73461 (+) Hs ARPC2
EP74595 (+) Hs SORD
EP73613 (+) Hs MGAT1
EP74600 (+) Hs STMN2
EP74598 (+) Hs PSMB4
EP73466 (+) Hs SDCCAG10
EP73220 (+) Hs TRAF2
EP74157 (+) Hs HIBCH
EP73906 (+) Hs PGLS
EP64003 (+) Hs TdT
EP30054 (+) Hs arginase liver
EP30056 (+) Hs OAT
EP73238 (+) Hs FLJ10525
EP73836 (+) Hs GMPPA
EP16033 (-) Hs a'-cardiac actin0
EP23004 (+) Hs ALDH_2 mit
EP73400 (+) Hs TOMM34
EP73505 (+) Hs RBM3
EP73064 (+) Hs OCIA
EP73511 (+) Hs ALDOC
EP73632 (+) Hs ASNS
EP25039 (-) Hs b'2-AR
EP73865 (+) Hs DDX24
EP73639 (+) Hs TXNRD1
EP27007 (+) Hs Bcl-2 E2P2
EP30017 (+) Hs PGK1
EP73532 (+) Hs GATM
EP73986 (+) Hs CKMT1
EP24039 (+) Hs vimentin
EP17067 (+) Hs glucagon
EP73540 (+) Hs CD83
EP73798 (+) Hs CIR
EP73290 (+) Hs LOC51705
EP73447 (+) Hs PAICS
EP73551 (+) Hs PRDX6
EP73669 (+) Hs SEMG1
EP73554 (+) Hs NDUFB9
EP74441 (+) Hs ETFB

EP74440 (+) Hs SFRS2
EP74596 (+) Hs ATP5G2
EP73464 (+) Hs PRDX2
EP73316 (+) Hs LOC51064
EP73033 (+) Hs TGFBI
EP73133 (+) Hs RPS6
EP73042 (+) Hs ADH1B
EP30035 (-) Hs PSG1 (PSb'G)
EP73040 (+) Hs DKFZP564G2022
EP73580 (+) Hs TSN
EP73443 (+) Hs CCT3
EP73146 (+) Hs ATP5J
EP73346 (+) Hs DDX25
EP73708 (+) Hs ACTA2
EP73595 (+) Hs EIF2S2
EP74593 (+) Hs SNRPD1
EP73751 (+) Hs NDUFB6
EP73752 (+) Hs NDUFS4
EP73943 (+) Hs SMC2L1
EP73603 (+) Hs P4HA2
EP73608 (+) Hs AHCY
EP73607 (+) Hs APEX1
EP73921 (+) Hs ATP5L
EP73475 (+) Hs SGK
EP74106 (+) Hs PAH
EP30039 (-) Hs CRF
EP73616 (+) Hs PHB
EP73617 (+) Hs RALB
EP73472 (+) Hs RBM5
EP73774 (+) Hs MLPH
EP28009 (-) Hs IGF II E4P4
EP29004 (-) Hs calcitonin/a'CGRP
EP74558 (+) Hs SDCBP
EP73971 (+) Hs NAT8
EP73439 (+) Hs NNMT
EP73633 (+) Hs ADM
EP07106 (-) Hs CG/LH/FSH/TSH a'0
EP14058 (-) Hs IFN-a'13
EP30074 (-) Hs FSH b'
EP73156 (+) Hs RTN4
EP73854 (+) Hs HSPC177
EP73113 (+) Hs MKLN1
EP73648 (+) Hs CPB1
EP73117 (+) Hs AKR1C3
EP73993 (+) Hs HRB2
EP73350 (+) Hs HSU15552
EP74281 (+) Hs PLDN
EP73265 (+) Hs MAPRE1
EP73664 (+) Hs PSMA2
EP73758 (+) Hs HDAC2
EP73830 (+) Hs ASAH1
EP28010 (+) Hs IGF II E2P2
EP73081 (+) Hs KIF22
EP73478 (+) Hs SLC35A2
EP33035 (+) Hs CNTF
EP73886 (+) Hs CRYL1
EP36006 (+) Hs AGT P3
EP74563 (+) Hs HPRT1 P1
EP73375 (+) Hs LYPLA3
EP74374 (+) Hs MRPL16
EP73687 (+) Hs UBE2D1
EP73949 (+) Hs HSPE1
EP74529 (+) Hs DHPS
EP73740 (+) Hs HSPD1
EP73889 (+) Hs FLJ10298
EP74035 (+) Hs PPP2R5C
EP73696 (+) Hs OMG
EP74153 (+) Hs SKP2
EP73700 (+) Hs PGM1
EP07113 (+) Hs g'-interferon
EP14063 (+) Hs IL-1a'
EP73623 (+) Hs TARS
EP74047 (+) Hs RAC1
EP73567 (+) Hs GYG
EP73822 (+) Hs PLEKHB2
EP73619 (+) Hs RBMX
EP73622 (+) Hs SSR1
EP25044 (-) Hs IL-5 (EDF/TRF)
EP74146 (+) Hs NIF3L1
EP73463 (+) Hs BCKDK
EP17083 (+) Hs G-CSF
EP73186 (+) Hs PRKCABP
EP73471 (+) Hs FRSB
EP73991 (+) Hs MRPS31
EP73653 (+) Hs GOT1
EP74311 (+) Hs PSMA3
EP73203 (+) Hs PSAP
EP73207 (+) Hs RPL22
EP74091 (+) Hs SERPINB1
EP60012 (+) Hs a'-1a-AdrenR
EP45002 (+) Hs endothelin-A rec.
EP74336 (+) Hs HLA DRB1
EP73797 (+) Hs ATIC
EP73118 (+) Hs ADH5
EP73394 (+) Hs LILRA2
EP73769 (+) Hs SEPT6
EP73460 (+) Hs ARPC5
EP74394 (+) Hs TNRC5
EP73968 (+) Hs ING3
EP73427 (+) Hs REG1B
EP73037 (+) Hs HBXIP
EP74271 (+) Hs HLA DRB3
EP73241 (+) Hs HIMAP4
EP25008 (+) Hs N-myc E1P1+
EP25009 (-) Hs N-myc E1P2
EP25010 (+) Hs N-myc E2P3
EP73159 (+) Hs FABP4
EP73045 (+) Hs CYP11A1
EP73535 (+) Hs TTC4
EP45003 (+) Hs endothelin-B rec.
EP73174 (+) Hs PRNP
EP74288 (+) Hs CKN1
EP73994 (+) Hs WBSCR22
EP74299 (+) Hs SELENBP1
EP73546 (+) Hs USP49
EP73185 (+) Hs CLU
EP73275 (+) Hs FLJ20320
EP73389 (+) Hs RPL41
EP28005 (+) Hs pepsinogen C
EP74439 (+) Hs UQCRH
EP73281 (+) Hs FANCL
EP73039 (+) Hs DDX18
EP11139 (+) Hs c-sis (PDGF2)
EP73428 (+) Hs SARS
EP73518 (+) Hs DNAJB6
EP74414 (+) Hs MAP2K1IP1
EP73408 (+) Hs BTG1
EP73736 (+) Hs GNAI2
EP73831 (+) Hs GLA
EP73807 (+) Hs GSN
EP73645 (+) Hs CPA1
EP07117 (+) Hs Ig k' HK101
EP73080 (+) Hs FLJ12525
EP73745 (+) Hs KPNB3
EP73313 (+) Hs LR8
EP74097 (+) Hs CAPZA1
EP73593 (+) Hs SNAP23
EP73365 (+) Hs D1S155E
EP73187 (+) Hs PON2
EP73437 (+) Hs MYL9
EP16068 (+) Hs MHCII HLA DQ2b'00
EP73881 (+) Hs ODC1
EP73078 (+) Hs ITM2C
EP73151 (+) Hs C5orf13
EP73859 (+) Hs LPL
EP73801 (+) Hs RDHL
EP73335 (+) Hs COX7C
EP73614 (+) Hs MTIF2
EP73657 (+) Hs ATP6V1E1

EP73289 (+) Hs YPEL5
EP73051 (+) Hs PDLIM1
EP73455 (+) Hs NOL5A
EP73229 (+) Hs CG005
EP73325 (+) Hs PAIP2
EP27006 (+) Hs Bcl-2 E1P1+
EP73586 (+) Hs APOL1
EP74332 (+) Hs APOA1BP
EP73462 (+) Hs ARPC1B
EP73235 (+) Hs SLC31A2
EP73746 (+) Hs MCM3
EP30036 (+) Hs PSG5
EP73109 (+) Hs FBP1
EP74452 (+) Hs TBCC
EP74358 (+) Hs ZWINT
EP73947 (+) Hs EIF3S6IP
EP73482 (+) Hs CSE1L
EP73925 (+) Hs GADD45B
EP73285 (+) Hs COX7A2
EP73031 (+) Hs C14ORF2
EP73168 (+) Hs ACTR6
EP73053 (+) Hs CRYAB
EP74541 (+) Hs POLR2J
EP74259 (+) Hs SPR
EP73391 (+) Hs TMSB10
EP74349 (+) Hs TPMT
EP26001 (+) Hs TCR va' HD-Mar
EP73499 (+) Hs CAPZB
EP73527 (+) Hs LGMN
EP73095 (+) Hs MEP50
EP73675 (+) Hs SRP14
EP73668 (+) Hs RPA2
EP73569 (+) Hs NAP1L1
EP74589 (+) Hs CLNS1A
EP74071 (+) Hs RARS
EP72002 (+) Hs AGT P2
EP30044 (-) Hs PTH P2
EP11098 (-) Hs z'-globin
EP73423 (+) Hs CCT8
EP73839 (+) Hs HNRPA3
EP73661 (+) Hs PRSS1
EP74381 (+) Hs BZW2 P1
EP74039 (+) Hs PTBP1
EP73165 (+) Hs RAB18
EP74292 (+) Hs MRPL13
EP73818 (+) Hs C14ORF100
EP74253 (+) Hs SRP19
EP74067 (+) Hs RPS24
EP73406 (+) Hs SLC25A5
EP73320 (+) Hs SBDS
EP73322 (+) Hs COPS4
EP73131 (+) Hs TFG
EP74467 (+) Hs QDPR
EP73199 (+) Hs CTRC
EP73923 (+) Hs SYNJ2BP
EP74360 (+) Hs METTL1
EP26027 (+) Hs th'-globin
EP73519 (+) Hs ATP5C1
EP73849 (+) Hs FADS1
EP73857 (+) Hs FH
EP73528 (+) Hs COPS3
EP73677 (+) Hs STATH
EP73781 (+) Hs GSS
EP73276 (+) Hs FLJ20420
EP74393 (+) Hs GNB2L1
EP73541 (+) Hs TRIP15
EP73556 (+) Hs HIF1A
EP73023 (+) Hs MGC2668
EP73374 (+) Hs FTSJ1
EP74540 (+) Hs CD79A
EP74154 (+) Hs TIMM13
EP73264 (+) Hs PROL4
EP73484 (+) Hs CTRB1
EP73094 (+) Hs HDAC1
EP73465 (+) Hs LHFP
EP73568 (+) Hs CITED1
EP73144 (+) Hs CDC5L
EP74407 (+) Hs PRKAB1
EP73673 (+) Hs SPARC
EP72001 (+) Hs AGT P1
EP74559 (+) Hs GYPC
EP73067 (+) Hs FLJ13154
EP73877 (+) Hs EIF3S3
EP73029 (+) Hs FLJ14904
EP73557 (+) Hs HRMT1L1
EP73897 (+) Hs DDX39
EP73666 (+) Hs PTPN6
EP73759 (+) Hs NME1
EP73654 (+) Hs HMOX1
EP73575 (+) Hs PIGH
EP73647 (+) Hs CPA3
EP74432 (+) Hs MOCS2
EP74508 (+) Hs GCSH
EP74491 (+) Hs ATP5O
EP73147 (+) Hs MASP1
EP74339 (+) Hs USP5
EP74074 (+) Hs IFNGR1
EP73280 (+) Hs FLJ10276
EP73702 (+) Hs POU2F1
EP73228 (+) Hs CCT2
EP73414 (+) Hs SIAT4C
EP73548 (+) Hs ITM2A
EP73894 (+) Hs TRAP1
EP73789 (+) Hs PSMD8
EP74585 (+) Hs NDUFA5

GGATATTT

EP73927 (+) Hs PTTG1IP

EP73928 (+) Hs RBM14

EP74563 (+) Hs HPRT1 P1

EP74616 (+) Hs COIL

EP74573 (+) Hs DAD1

EP74615 (+) Hs PRCC

EP73940 (+) Hs NEU1

EP73942 (+) Hs TSG101

EP73947 (+) Hs EIF3S6IP

EP73946 (+) Hs HEXA

EP74630 (+) Hs VAPA

EP73957 (+) Hs APOH

EP73960 (+) Hs HNRPK P2+

EP73714 (+) Hs PSMB1

EP58019 (+) Hs WT1

EP73878 (+) Hs AP1S2

EP64001 (+) Hs rag-1

EP73985 (+) Hs CDC23

EP74367 (+) Hs DKC1

EP73988 (+) Hs APEX2

EP16071 (+) Hs[ERV3]

EP74343 (+) Hs MT1L

EP47007 (+) Hs HDC

EP74093 (+) Hs TDO2

EP45002 (+) Hs endothelin-A rec.

EP74006 (+) Hs GRHPR

EP73760 (+) Hs AHSA1

EP74009 (+) Hs VAT1

EP11141 (+) Hs estrogen receptor

EP11145 (+) Hs FOS

EP74018 (+) Hs AATF

EP73771 (+) Hs SNX6

EP73238 (+) Hs FLJ10525

EP74025 (+) Hs SDHD

EP25008 (+) Hs N-myc E1P1+

EP74028 (+) Hs UBE2L6

EP27006 (+) Hs Bcl-2 E1P1+

EP74035 (+) Hs PPP2R5C

EP74036 (+) Hs IMPA1

EP73937 (+) Hs RFC3

EP74039 (+) Hs PTBP1

EP73797 (+) Hs ATIC

EP74049 (+) Hs EIF4A2

EP74052 (+) Hs LIG1

EP74063 (+) Hs CDKN1A

EP37011 (-) Hs MT-IG

EP74065 (+) Hs PSMB8

EP74066 (+) Hs MRPS16

EP74417 (+) Hs SDHB

EP74077 (+) Hs RPS9 P1

EP73827 (+) Hs DC6

EP74083 (+) Hs LAMR1

EP74097 (+) Hs CAPZA1

EP74446 (+) Hs APLP1 P1

EP73599 (+) Hs UQCRC1

EP74102 (+) Hs TCEB1 P1

EP74106 (+) Hs PAH

EP74109 (+) Hs DYT1

EP74115 (+) Hs ZNF265

EP11160 (+) Hs CD3 (T3) d'

EP74469 (+) Hs SPARCL1 P1

EP74125 (+) Hs WRB P1

EP74131 (+) Hs SLC25A4

EP73006 (+) Hs CAV1

EP74484 (+) Hs TNFAIP1

EP74136 (+) Hs TIMM17A

EP74143 (+) Hs TUBG1

EP74146 (+) Hs NIF3L1

EP74154 (+) Hs TIMM13

EP73031 (+) Hs C14ORF2

EP73034 (+) Hs GBAS

EP73036 (+) Hs SH3BGRL3

EP74521 (+) Hs JUN

EP74173 (+) Hs ART3

EP74185 (+) Hs FTSJ3

EP74186 (+) Hs TOR3A

EP74187 (+) Hs PLGL

EP73688 (+) Hs UBE2D3

EP73059 (+) Hs GALE

EP74193 (+) Hs APG12L

EP74197 (+) Hs GPX2

EP74547 (+) Hs HMGB2

EP73073 (+) Hs SID6

EP73074 (+) Hs DDX5

EP07114 (+) Hs IL-2 (TCGF)

EP73076 (+) Hs LOC81501

EP74208 (+) Hs NFKBIL1

EP73077 (+) Hs ALDH7A1

EP73079 (+) Hs TMEM14B

EP73080 (+) Hs FLJ12525

EP73712 (+) Hs PRPS1

EP73093 (+) Hs CASQ2

EP73969 (+) Hs PPAN

EP11105 (-) Hs d'-globin

EP73723 (+) Hs SFRS3

EP74229 (+) Hs RNASE4

EP74233 (+) Hs ANP32A

EP73105 (+) Hs LGTN

EP74242 (+) Hs MRPL50

EP74244 (+) Hs MRPL9

EP74247 (+) Hs GLRX

EP17030 (-) Hs snRNA U1 (pU1-6)

EP17031 (-) Hs snRNA U1 (pHU1-1)

EP73459 (+) Hs CTNNA1

EP74251 (+) Hs GARS P1

EP74256 (+) Hs ANXA5

EP74604 (+) Hs HLA-DPA1

EP74258 (+) Hs FXYD3

EP07056 (+) Hs DHFR

EP15041 (+) Hs transferrin rec.

EP73133 (+) Hs RPS6

EP74267 (+) Hs HSPA9B P1

EP74268 (+) Hs HSPA9B P2+

EP74270 (+) Hs PRAME

EP73767 (+) Hs SOD2

EP73144 (+) Hs CDC5L

EP73149 (+) Hs RAB1A

EP73152 (+) Hs NDUFA9

EP74286 (+) Hs CASP6

EP73155 (+) Hs PSMF1

EP74291 (+) Hs FXYD5

EP74299 (+) Hs SELENBP1

EP73795 (+) Hs SP100

EP74050 (+) Hs EDF1

EP73176 (+) Hs SPUVE

EP74311 (+) Hs PSMA3

EP73179 (+) Hs FLJ13868

EP73808 (+) Hs IL2RG

EP74317 (+) Hs RPLP0 P1

EP74318 (+) Hs RPLP0 P2+

EP11073 (+) Hs histone H3b

EP15024 (+) Hs histone H3.3

EP73190 (+) Hs SERPINA3

EP73095 (+) Hs MEP50

EP49012 (-) Hs histone H4t

EP73199 (+) Hs CTRC

EP73543 (+) Hs TM9SF2

EP74336 (+) Hs HLA DRB1

EP73206 (+) Hs FLJ22965

EP73208 (+) Hs RPL32

EP74345 (+) Hs ARL4 P2+

EP74344 (+) Hs ARL4 P1

EP74091 (+) Hs SERPINB1

EP74354 (+) Hs SERPINB6

EP73565 (+) Hs FDFT1

EP74105 (+) Hs PRKRA

EP30051 (-) Hs stromelysin

EP73234 (+) Hs DLD

EP41007 (+) Hs rp S17

EP73045 (+) Hs CYP11A1
EP74376 (+) Hs HEXB
EP73250 (+) Hs VPS35
EP74385 (+) Hs SNRPD2
EP74387 (+) Hs TAX1BP1
EP73256 (+) Hs HMT
EP73259 (+) Hs DAB2
EP74396 (+) Hs IMPA2
EP74401 (+) Hs MRPS23
EP74402 (+) Hs SNURF
EP74403 (+) Hs RBM7
EP74151 (+) Hs BCAS2
EP74414 (+) Hs MAP2K1IP1
EP74415 (+) Hs HTATIP
EP74416 (+) Hs JTB
EP26019 (+) Hs neurofilament L
EP73912 (+) Hs CSRP2
EP73915 (+) Hs VCP P1
EP73299 (+) Hs ACTR3
EP16038 (+) Hs fibronectin
EP60004 (+) Hs link
EP17050 (+) Hs vWf
EP73647 (+) Hs CPA3
EP74184 (+) Hs SSSCA1
EP74444 (+) Hs CLIC1 P2+
EP30043 (-) Hs PTH P1+
EP74447 (+) Hs APLP1 P2+
EP73658 (+) Hs ADFP
EP15029 (+) Hs b'-fibrinogen
EP74450 (+) Hs PSMD2
EP74463 (+) Hs CPE
EP74465 (+) Hs DAZAP2
EP74470 (+) Hs SPARCL1 P2+
EP73686 (+) Hs TPT1
EP73343 (+) Hs TM4SF13
EP14058 (-) Hs IFN-a'13
EP74481 (+) Hs MRPL49
EP14060 (-) Hs IFN-a'5
EP11135 (-) Hs IFN l'2h (-a'14)
EP73698 (+) Hs PBP
EP74388 (+) Hs MAT2B
EP74497 (+) Hs GABARAP P2+
EP73372 (+) Hs HBP1
EP73427 (+) Hs REG1B
EP73724 (+) Hs SH3BGRL
EP74519 (+) Hs EIF3S5
EP73731 (+) Hs CALM2
EP11112 (-) Hs ferritin H
EP25049 (-) Hs ferritin L
EP74533 (+) Hs MEST
EP74536 (+) Hs GCA
EP73987 (+) Hs MLANA
EP73158 (+) Hs RPL26
EP73408 (+) Hs BTG1
EP74546 (+) Hs UCP2
EP73277 (+) Hs FLJ20424
EP73416 (+) Hs SPTLC1
EP74550 (+) Hs ATP5G1
EP11087 (+) Hs g'-fibrinogen
EP74555 (+) Hs HLA-DPB1
EP74309 (+) Hs RPS15A
EP74564 (+) Hs HPRT1 P2+
EP74119 (+) Hs HLA DQA1
EP74570 (+) Hs TRA1
EP74574 (+) Hs CCT6A P1
EP74582 (+) Hs S100B
EP73450 (+) Hs SLC35A1
EP74588 (+) Hs NFYB
EP35012 (+) Hs P-glycoprotein 1
EP73910 (+) Hs CLTA
EP74589 (+) Hs CLNS1A
EP74592 (+) Hs FADD
EP74593 (+) Hs SNRPD1
EP74090 (+) Hs SLC1A5
EP73614 (+) Hs MTIF2
EP74601 (+) Hs PGRMC1
EP73465 (+) Hs LHFP
EP25036 (+) Hs MT-IB
EP73468 (+) Hs P5
EP30058 (-) Hs MT-IE
EP73814 (+) Hs KIAA0174
EP73281 (+) Hs FANCL
EP73624 (+) Hs TCF12
EP73476 (+) Hs SR
EP14030 (+) Hs a'-tubulin ba'1
EP73831 (+) Hs GLA
EP73604 (+) Hs LIPF
EP42001 (+) Hs BCKDHA
EP74381 (+) Hs BZW2 P1
EP74382 (+) Hs BZW2 P2+
EP73503 (+) Hs LDHA
EP17058 (+) Hs LCAT
EP73508 (+) Hs ANXA4
EP25021 (+) Hs IFIT1
EP73512 (+) Hs CALM3
EP48007 (+) Hs TNNI1
EP74321 (+) Hs POLR2K
EP30039 (-) Hs CRF
EP73524 (+) Hs IFI27
EP73525 (+) Hs MEP1A
EP73982 (+) Hs MTAP
EP73872 (+) Hs DC50
EP73884 (+) Hs LIPA
EP73012 (+) Hs MGC3248
EP73011 (+) Hs NTPBP
EP73891 (+) Hs CMAS
EP73022 (+) Hs CPSF3
EP73369 (+) Hs COG2
EP73030 (+) Hs MGC14156
EP73568 (+) Hs CITED1
EP25044 (-) Hs IL-5 (EDF/TRF)
EP73576 (+) Hs RAB5A
EP73586 (+) Hs APOL1
EP73591 (+) Hs EIF3S2
EP73592 (+) Hs EIF3S1
EP73494 (+) Hs CCNB2
EP11068 (+) Hs histone H2A
EP73602 (+) Hs YWHAB
EP73943 (+) Hs SMC2L1
EP11134 (-) Hs IFN l'2c1 (-a'17)
EP73312 (+) Hs SDCBP2
EP16046 (-) Hs a'-fetoprotein
EP30078 (-) Hs THYL
EP74504 (+) Hs ARF5
EP73484 (+) Hs CTRB1
EP73625 (+) Hs UGDH
EP73745 (+) Hs KPNB3
EP74022 (+) Hs SFRS5
EP74513 (+) Hs PPIG
EP73640 (+) Hs UBE2A
EP74103 (+) Hs TCEB1 P2+
EP73467 (+) Hs LRRC17
EP73411 (+) Hs CDIPT
EP73130 (+) Hs CRACC
EP73417 (+) Hs ANP32B
EP73821 (+) Hs FLJ20422
EP73140 (+) Hs PFDN5
EP73480 (+) Hs MAT2A
EP74369 (+) Hs VRK1
EP73734 (+) Hs FNTB
EP73153 (+) Hs NDUFS1
EP74031 (+) Hs GOT2
EP32001 (+) Hs protein C
EP73842 (+) Hs TES
EP74034 (+) Hs SKP1A
EP30057 (-) Hs MT-IA
EP74040 (+) Hs VAMP8
EP73391 (+) Hs TMSB10
EP30003 (+) Hs c-N-ras
EP17079 (+) Hs IL1B

EP73796 (+) Hs SRP54
EP74590 (+) Hs WDR1
EP73455 (+) Hs NOL5A
EP73456 (+) Hs CHI3L1
EP73177 (+) Hs MKKS
EP73716 (+) Hs PSMC1
EP73807 (+) Hs GSN
EP73801 (+) Hs RDHL
EP73273 (+) Hs SC5DL
EP74407 (+) Hs PRKAB1
EP73945 (+) Hs S100A10
EP14063 (+) Hs IL-1a'
EP73086 (+) Hs STOML2
EP73532 (+) Hs GATM
EP73533 (+) Hs ANXA1
EP73728 (+) Hs SNX2
EP73732 (+) Hs CAPG
EP74165 (+) Hs KRT8
EP73315 (+) Hs OVCOV1
EP73202 (+) Hs IK
EP73205 (+) Hs LPXN
EP25050 (+) Hs surfactant p. 5K
EP73260 (+) Hs COX4I1
EP74567 (+) Hs POLD4
EP73560 (+) Hs B2M
EP11142 (+) Hs IL-2 receptor P1
EP73041 (+) Hs PEF
EP17093 (+) Hs TCR vb'8.1
EP11104 (+) Hs b'-globin
EP40004 (+) Hs C4BP b' A19 mRNA

EP73765 (+) Hs PKIA
EP73050 (+) Hs CDC10
EP73003 (+) Hs RPS8
EP73518 (+) Hs DNAJB6
EP74457 (+) Hs TFAM
EP73923 (+) Hs SYNJ2BP
EP73469 (+) Hs PBEF
EP73246 (+) Hs C6ORF37
EP73579 (+) Hs TM4SF3
EP27007 (+) Hs Bcl-2 E2P2
EP73931 (+) Hs RPS25
EP73536 (+) Hs UCHL1
EP73877 (+) Hs EIF3S3
EP73365 (+) Hs D1S155E
EP73258 (+) Hs RPL37A
EP73546 (+) Hs USP49
EP74166 (+) Hs ARL1
EP74142 (+) Hs RFX4
EP73357 (+) Hs MGP
EP73553 (+) Hs NDUFAB1
EP73616 (+) Hs PHB
EP73211 (+) Hs DHRS6
EP73561 (+) Hs CLK1
EP73291 (+) Hs LOC51706
EP74252 (+) Hs GARS P2+
EP74426 (+) Hs VPS29
EP73056 (+) Hs RPL27A
EP74629 (+) Hs PFKP
EP73061 (+) Hs ITM2B
EP73916 (+) Hs VCP P2+

EP73578 (+) Hs TM4SF2
EP73880 (+) Hs RPL35
EP16050 (+) Hs HMG-CoA red.
EP73588 (+) Hs STC2
EP07117 (+) Hs Ig k' HK101
EP25083 (+) Hs rhodopsin
EP73644 (+) Hs COPEB
EP73670 (+) Hs SEMG2
EP17094 (-) Hs TCR vb'8.2
EP73367 (+) Hs ATF6
EP73606 (+) Hs AKR1B1
EP73991 (+) Hs MRPS31
EP73327 (+) Hs HSPC016
EP73103 (+) Hs CBARA1
EP74500 (+) Hs MRPL27
EP74562 (+) Hs PSMA1
EP73339 (+) Hs SMP1
EP73340 (+) Hs E2IG3
EP16037 (+) Hs involucrin
EP40002 (+) Hs complement C5
EP73231 (+) Hs PES1
EP27010 (+) Hs L-myc
EP25010 (+) Hs N-myc E2P3
EP73918 (+) Hs ATP6V0D1
EP73466 (+) Hs SDCCAG10
EP74240 (+) Hs PTGES2
EP74440 (+) Hs SFRS2
EP30071 (+) Hs TSH b'
EP73823 (+) Hs ESRRBL1
EP73772 (+) Hs FLJ13188

EP28005 (+) Hs pepsinogen C
EP73886 (+) Hs CRYL1
EP73617 (+) Hs RALB
EP73055 (+) Hs HNRPR
EP73432 (+) Hs BTF3
EP74011 (+) Hs DCTN3
EP73334 (+) Hs CD14
EP73920 (+) Hs MRPS22
EP73371 (+) Hs CHORDC1
EP73243 (+) Hs C20ORF29
EP73255 (+) Hs BAP29
EP73167 (+) Hs FLJ13390
EP73301 (+) Hs AF1Q
EP74432 (+) Hs MOCS2
EP30017 (+) Hs PGK1
EP30044 (-) Hs PTH P2
EP74298 (+) Hs WBP11
EP73861 (+) Hs PP1201
EP73751 (+) Hs NDUFB6
EP74292 (+) Hs MRPL13
EP74522 (+) Hs IMMT
EP73747 (+) Hs MIF
EP74111 (+) Hs BIN1
EP74117 (+) Hs APCS
EP73705 (+) Hs PRKAR1A
EP73443 (+) Hs CCT3
EP73857 (+) Hs FH
EP73959 (+) Hs HNRPK P1
EP73314 (+) Hs HIP
EP74078 (+) Hs RPS9 P2+

EP74378 (+) Hs CCNH
EP73316 (+) Hs LOC51064
EP73965 (+) Hs RNF7
EP36010 (-) Hs NCA
EP73382 (+) Hs PNLIP
EP73435 (+) Hs MDH1
EP73192 (+) Hs SERPIND1
EP73311 (+) Hs GORASP2
EP74366 (+) Hs SYAP1
EP73377 (+) Hs ARFIP2
EP73383 (+) Hs PPIB
EP74253 (+) Hs SRP19
EP73397 (+) Hs RNP24
EP73876 (+) Hs RAE1
EP74248 (+) Hs HPD
EP73087 (+) Hs DREV1
EP73431 (+) Hs BAK1
EP73646 (+) Hs CPA2
EP74003 (+) Hs PRPF18
EP73835 (+) Hs PECI
EP73222 (+) Hs LOC57862
EP73249 (+) Hs FLJ10509
EP73888 (+) Hs C20ORF43
EP74126 (+) Hs WRB P2+
EP73917 (+) Hs CLPTM1
EP73398 (+) Hs TDE1
EP73913 (+) Hs DFFA
EP73161 (+) Hs PIGPC1
EP74454 (+) Hs AARS
EP73696 (+) Hs OMG

EP73700 (+) Hs PGM1
EP74389 (+) Hs BAT1
EP11127 (-) Hs LH b'
EP73538 (+) Hs USP11
EP73016 (+) Hs RPS12
EP73386 (+) Hs XPNPEP1
EP74496 (+) Hs GABARAP P1
EP73322 (+) Hs COPS4
EP73675 (+) Hs SRP14
EP73239 (+) Hs C20ORF44
EP73321 (+) Hs LOC51125
EP73834 (+) Hs LYZ
EP73325 (+) Hs PAIP2
EP11110 (-) Hs e'-globin
EP74587 (+) Hs TTID
EP73887 (+) Hs RDH11
EP30036 (+) Hs PSG5
EP73669 (+) Hs SEMG1
EP73652 (+) Hs GDI2
EP73154 (+) Hs RPL10
EP73118 (+) Hs ADH5
EP74289 (+) Hs SEPP1
EP73863 (+) Hs PEPD
EP73424 (+) Hs PROL3
EP73790 (+) Hs PSMD11
EP74443 (+) Hs CLIC1 P1
EP73333 (+) Hs LTA4H
EP73584 (+) Hs DCTD
EP73399 (+) Hs TMP21
EP73873 (+) Hs FAM14A

EP73554 (+) Hs NDUFB9
EP73471 (+) Hs FRSB
EP73108 (+) Hs CD44
EP73535 (+) Hs TTC4
EP73645 (+) Hs CPA1
EP73474 (+) Hs SERF2
EP73779 (+) Hs MGC13204
EP74132 (+) Hs PAK4
EP73323 (+) Hs LOC51185
EP73175 (+) Hs PXMP3
EP25009 (-) Hs N-myc E1P2
EP73744 (+) Hs KPNB2
EP74575 (+) Hs CCT6A P2+
EP11070 (+) Hs histone H2B
EP11143 (+) Hs IL-2 receptor P2
EP74110 (+) Hs UBE2G2
EP74471 (+) Hs HSPA2
EP73193 (+) Hs RGS4
EP07121 (+) Hs MHCII HLA-DRA
EP73294 (+) Hs SLC38A2
EP73595 (+) Hs EIF2S2
EP74246 (+) Hs DDX19
EP73841 (+) Hs NAPA
EP74029 (+) Hs ETFA
EP73029 (+) Hs FLJ14904
EP73393 (+) Hs NDUFV1
EP73229 (+) Hs CG005
EP73542 (+) Hs TXNL
EP74377 (+) Hs NDUFV2

GGTTATTT

EP74605 (+) Hs PMSCL1 P1

EP74606 (+) Hs PMSCL1 P2+

EP74609 (+) Hs EIF4B

EP73433 (+) Hs ATF4

EP74614 (+) Hs TSNAX

EP74573 (+) Hs DAD1

EP11068 (+) Hs histone H2A

EP14061 (-) Hs IFN-a'16

EP74622 (+) Hs PDHB

EP74624 (+) Hs NUP62

EP74629 (+) Hs PFKP

EP73952 (+) Hs SNX3 P2+

EP73953 (+) Hs ELAC2

EP73954 (+) Hs WDR13

EP73959 (+) Hs HNRPK P1

EP39004 (+) Hs CSRP1

EP56002 (+) Hs glycophorin A

EP56004 (-) Hs glycophorin E

EP56003 (-) Hs glycophorin B

EP25021 (+) Hs IFIT1

EP73967 (+) Hs NOLA3

EP11139 (+) Hs c-sis (PDGF2)

EP73970 (+) Hs CUL1

EP33035 (+) Hs CNTF

EP74608 (+) Hs MRPL24

EP73727 (+) Hs SNX1

EP73196 (+) Hs EBNA1BP2

EP64001 (+) Hs rag-1

EP31007 (+) Hs HMG-14

EP33023 (+) Hs TNP1

EP74338 (+) Hs COPS7A P2+

EP25083 (+) Hs rhodopsin

EP25085 (-) Hs opsin red

EP74344 (+) Hs ARL4 P1

EP74353 (+) Hs BUB3

EP11143 (+) Hs IL-2 receptor P2

EP74017 (+) Hs PIN1

EP35031 (+) Hv RuBPCA RcaA

EP74369 (+) Hs VRK1

EP73237 (+) Hs RNPC4

EP11148 (+) Hs c-myc P2+

EP74028 (+) Hs UBE2L6

EP74378 (+) Hs CCNH

EP74031 (+) Hs GOT2

EP74033 (+) Hs HADH2

EP60011 (+) Hs Cyclin D1

EP17089 (+) Hs HSPA8

EP74076 (+) Hs RPS4Y

EP74084 (+) Hs UFD1L

EP74085 (+) Hs HNRPC

EP73847 (+) Hs RPS27

EP73598 (+) Hs UNG

EP26001 (+) Hs TCR va' HD-Mar

EP74104 (+) Hs ACPP

EP74106 (+) Hs PAH

EP74107 (+) Hs PACSIN2

EP73856 (+) Hs CLN2

EP74113 (+) Hs TARDBP

EP11160 (+) Hs CD3 (T3) d'

EP74117 (+) Hs APCS

EP73868 (+) Hs ALDH6A1

EP73875 (+) Hs SMARCE1

EP74480 (+) Hs CRI1

EP74134 (+) Hs UMPS

EP25014 (+) Hs 2'5'-oligoA synt.

EP73011 (+) Hs NTPBP

EP74143 (+) Hs TUBG1

EP73370 (+) Hs GTF3C5

EP74157 (+) Hs HIBCH

EP74159 (+) Hs RAB11B

EP74161 (+) Hs CCNB1

EP74002 (+) Hs STK25 P3+

EP74546 (+) Hs UCP2

EP74523 (+) Hs DDX1

EP73046 (+) Hs RPS10

EP73051 (+) Hs PDLIM1

EP74186 (+) Hs TOR3A

EP73058 (+) Hs GPX1

EP74190 (+) Hs DPAGT1

EP73648 (+) Hs CPB1

EP73063 (+) Hs ABP1

EP73066 (+) Hs MMS19L

EP74197 (+) Hs GPX2

EP74202 (+) Hs SCAND1

EP74203 (+) Hs MRPS18B

EP73073 (+) Hs SID6

EP73951 (+) Hs SNX3 P1

EP74206 (+) Hs EEF1B2 P1

EP74207 (+) Hs EEF1B2 P2+

EP74208 (+) Hs NFKBIL1

EP74213 (+) Hs APLP2

EP73712 (+) Hs PRPS1

EP73081 (+) Hs KIF22

EP73960 (+) Hs HNRPK P2+

EP73963 (+) Hs CAMLG

EP74218 (+) Hs HNRPH3

EP74223 (+) Hs CRSP9

EP74570 (+) Hs TRA1

EP73723 (+) Hs SFRS3

EP74227 (+) Hs LYPLA2

EP74233 (+) Hs ANP32A

EP74234 (+) Hs RPL4

EP74237 (+) Hs RPL35A P1

EP73109 (+) Hs FBP1

EP74238 (+) Hs RPL35A P2+

EP74245 (+) Hs BCL2L12

EP73115 (+) Hs FLJ10769

EP73119 (+) Hs TF

EP73999 (+) Hs STK25 P1

EP73127 (+) Hs EFEMP2

EP17041 (-) Hs snRNA U4C

EP74259 (+) Hs SPR

EP73131 (+) Hs TFG

EP73759 (+) Hs NME1

EP74265 (+) Hs PQBP1

EP73475 (+) Hs SGK

EP74268 (+) Hs HSPA9B P2+

EP73138 (+) Hs B3GAT3

EP49001 (+) Hs histone H1t

EP74279 (+) Hs NDUFA8

EP74284 (+) Hs TIMM9

EP74286 (+) Hs CASP6

EP73158 (+) Hs RPL26

EP74298 (+) Hs WBP11

EP11070 (+) Hs histone H2B

EP73661 (+) Hs PRSS1

EP74311 (+) Hs PSMA3

EP74312 (+) Hs MRPL10

EP73181 (+) Hs FXR1

EP11073 (+) Hs histone H3b

EP74322 (+) Hs CYB561

EP74325 (+) Hs DUT

EP74324 (+) Hs GNG5

EP73193 (+) Hs RGS4

EP49012 (-) Hs histone H4t

EP73197 (+) Hs MCP

EP73543 (+) Hs TM9SF2

EP73294 (+) Hs SLC38A2

EP73548 (+) Hs ITM2A

EP74340 (+) Hs GUK1

EP74342 (+) Hs FXYD6

EP73302 (+) Hs PRDX4

EP74345 (+) Hs ARL4 P2+

EP74096 (+) Hs PP

EP36018 (-) Hs nucleolin

EP41008 (+) Hs snRNP E

EP74358 (+) Hs ZWINT

EP74367 (+) Hs DKC1

EP73577 (+) Hs TGM2

EP73865 (+) Hs DDX24
EP74374 (+) Hs MRPL16
EP73240 (+) Hs FLJ11000
EP74376 (+) Hs HEXB
EP73337 (+) Hs RRP4
EP74379 (+) Hs GMFG
EP73055 (+) Hs HNRPR
EP74382 (+) Hs BZW2 P2+
EP74381 (+) Hs BZW2 P1
EP74383 (+) Hs TBL2
EP74384 (+) Hs NDUFS3
EP73263 (+) Hs JWA
EP74396 (+) Hs IMPA2
EP74395 (+) Hs MCM2
EP74142 (+) Hs RFX4
EP73948 (+) Hs KHDRBS1
EP74402 (+) Hs SNURF
EP74408 (+) Hs TAF9
EP74309 (+) Hs RPS15A
EP07105 (-) Hs POMC
(ACTH,b'LPH)
EP73280 (+) Hs FLJ10276
EP74413 (+) Hs PCBP2
EP73032 (+) Hs PLP1
EP26019 (+) Hs neurofilament L
EP74515 (+) Hs KDELR1 P1
EP73291 (+) Hs LOC51706
EP74436 (+) Hs AUTL1
EP74441 (+) Hs ETFB
EP74439 (+) Hs UQCRH
EP73311 (+) Hs GORASP2
EP74114 (+) Hs MAD2L2
EP73319 (+) Hs ZDHHC9
EP73318 (+) Hs ADIPOR1
EP73669 (+) Hs SEMG1
EP74462 (+) Hs PSME1
EP74463 (+) Hs CPE
EP74467 (+) Hs QDPR
EP73579 (+) Hs TM4SF3
EP35012 (+) Hs P-glycoprotein 1
EP74471 (+) Hs HSPA2
EP73689 (+) Hs UBE2L3
EP14058 (-) Hs IFN-a'13
EP74483 (+) Hs TMSB4X
EP73349 (+) Hs HSU53209
EP74489 (+) Hs ATP1A1
EP74492 (+) Hs ATP1B3
EP16046 (-) Hs a'-fetoprotein
EP73111 (+) Hs RNF2
EP73362 (+) Hs SLC2A3
EP73365 (+) Hs D1S155E
EP74502 (+) Hs SNRPG
EP74506 (+) Hs PSMD10
EP74507 (+) Hs PLK
EP74512 (+) Hs ERCC3
EP74513 (+) Hs PPIG
EP74516 (+) Hs KDELR1 P2+
EP73192 (+) Hs SERPIND1
EP74075 (+) Hs WBSCR1
EP73539 (+) Hs PRPF4
EP73199 (+) Hs CTRC
EP74527 (+) Hs MLF2 P1
EP74528 (+) Hs MLF2 P2+
EP73145 (+) Hs ST13
EP74530 (+) Hs MRPL3 P1
EP74531 (+) Hs MRPL3 P2+
EP73397 (+) Hs RNP24
EP74533 (+) Hs MEST
EP73745 (+) Hs KPNB3
EP73153 (+) Hs NDUFS1
EP74539 (+) Hs H2AFO
EP74538 (+) Hs YWHAE
EP73408 (+) Hs BTG1
EP73335 (+) Hs COX7C
EP74549 (+) Hs NTS
EP73417 (+) Hs ANP32B
EP48005 (+) Hs VLDL rec.
EP73419 (+) Hs TIMM23
EP74304 (+) Hs DAXX
EP73426 (+) Hs PSMC4
EP73885 (+) Hs NOSIP
EP73924 (+) Hs HN1
EP73778 (+) Hs RAB7
EP74571 (+) Hs PRIM1
EP25009 (-) Hs N-myc E1P2
EP07077 (-) Hs ^Ag'-globin
EP73443 (+) Hs CCT3
EP73789 (+) Hs PSMD8
EP74582 (+) Hs S100B
EP73029 (+) Hs FLJ14904
EP74584 (+) Hs CKS2
EP26030 (+) Hs c-abl 7 kb E1P1
EP48003 (+) Hs collagenaseIV 72K
EP74590 (+) Hs WDR1
EP74593 (+) Hs SNRPD1
EP74493 (+) Hs RPS3A
EP73216 (+) Hs MYL6
EP73364 (+) Hs CORO1A
EP74601 (+) Hs PGRMC1
EP74602 (+) Hs ADRM1
EP73469 (+) Hs PBEF
EP73478 (+) Hs SLC35A2
EP11114 (+) Hs ASS
EP30056 (+) Hs OAT
EP73601 (+) Hs VDAC2
EP73242 (+) Hs FLJ11160
EP73497 (+) Hs GSTO1
EP73616 (+) Hs PHB
EP16050 (+) Hs HMG-CoA red.
EP68001 (-) Hs CA2
EP73261 (+) Hs DEPP
EP73514 (+) Hs GPD1
EP73384 (+) Hs CA12
EP26015 (+) Hs ALDOA E1P1
EP73116 (+) Hs TOPK
EP31010 (+) Hs hepatic lipase
EP73538 (+) Hs USP11
EP73353 (+) Hs STAF65
EP73255 (+) Hs BAP29
EP73894 (+) Hs TRAP1
EP07095 (+) Hs factor IX
EP73556 (+) Hs HIF1A
EP73900 (+) Hs MAP1LC3B
EP73558 (+) Hs ANXA11
EP16056 (-) Hs renin
EP74440 (+) Hs SFRS2
EP73902 (+) Hs RPL10A
EP28005 (+) Hs pepsinogen C
EP74600 (+) Hs STMN2
EP73923 (+) Hs SYNJ2BP
EP73574 (+) Hs PET112L
EP73040 (+) Hs DKFZP564G2022
EP73041 (+) Hs PEF
EP73387 (+) Hs SFRS7
EP73582 (+) Hs EIF3S6
EP73583 (+) Hs AMD1
EP73589 (+) Hs RTCD1
EP73788 (+) Hs PSMD1
EP74146 (+) Hs NIF3L1
EP74056 (+) Hs MIC2
EP73602 (+) Hs YWHAB
EP73809 (+) Hs MYL2
EP73614 (+) Hs MTIF2
EP73956 (+) Hs MAGEE1
EP73619 (+) Hs RBMX
EP73101 (+) Hs NP
EP73863 (+) Hs PEPD
EP16058 (+) Hs somatostatin I
EP73624 (+) Hs TCF12
EP29004 (-) Hs calcitonin/a'CGRP

EP74317 (+) Hs RPLP0 P1
EP73207 (+) Hs RPL22
EP73628 (+) Hs PDHX
EP74318 (+) Hs RPLP0 P2+
EP16068 (+) Hs MHCII HLA DQ2b'
EP74071 (+) Hs RARS
EP73103 (+) Hs CBARA1
EP26025 (+) Hs MRP-8
EP73653 (+) Hs GOT1
EP73707 (+) Hs C1S
EP74116 (+) Hs SAA4
EP59011 (+) Hs elk-1
EP14057 (+) Hs thyroglobulin
EP73129 (+) Hs ECH1
EP74354 (+) Hs SERPINB6
EP73471 (+) Hs FRSB
EP28010 (+) Hs IGF II E2P2
EP17072 (-) Hs IGF II E3P3
EP73676 (+) Hs SRP9
EP28009 (-) Hs IGF II E4P4
EP73726 (+) Hs SNAP25
EP73827 (+) Hs DC6
EP26031 (-) Hs c-abl 7 kb E1P2
EP73603 (+) Hs P4HA2
EP73264 (+) Hs PROL4
EP73799 (+) Hs CNIH
EP73687 (+) Hs UBE2D1
EP73155 (+) Hs PSMF1
EP11135 (-) Hs IFN l'2h (-a'14)
EP11107 (-) Hs ^Gg'-globin
EP11134 (-) Hs IFN l'2c1 (-a'17)
EP73699 (+) Hs PFN2
EP14063 (+) Hs IL-1a'
EP74589 (+) Hs CLNS1A
EP73382 (+) Hs PNLIP
EP74144 (+) Hs RNF5
EP74595 (+) Hs SORD
EP73519 (+) Hs ATP5C1
EP73805 (+) Hs SMT3H2
EP73717 (+) Hs PSMC5
EP73079 (+) Hs TMEM14B
EP74063 (+) Hs CDKN1A
EP73189 (+) Hs SERPINB2
EP73864 (+) Hs CYP3A5
EP74069 (+) Hs SCARB1
EP33011 (+) Hs DES
EP17088 (+) Hs hsp 70K
EP73957 (+) Hs APOH
EP15044 (+) Hs EGF receptor P2
EP74118 (+) Hs KRT5
EP26027 (+) Hs th'-globin
EP73834 (+) Hs LYZ
EP73716 (+) Hs PSMC1
EP73495 (+) Hs SCYE1
EP73213 (+) Hs TOMM22
EP73440 (+) Hs PPIE
EP73702 (+) Hs POU2F1
EP11142 (+) Hs IL-2 receptor P1
EP73448 (+) Hs AGR2
EP73225 (+) Hs RISC
EP74256 (+) Hs ANXA5
EP73625 (+) Hs UGDH
EP73369 (+) Hs COG2
EP17084 (+) Hs IgE rec. FC_e'R
EP73859 (+) Hs LPL
EP73234 (+) Hs DLD
EP73464 (+) Hs PRDX2
EP73752 (+) Hs NDUFS4
EP73288 (+) Hs LOC51629
EP73106 (+) Hs ZFP36L1
EP26017 (+) Hs ALDOA E4P3
EP73015 (+) Hs RPS11
EP25008 (+) Hs N-myc E1P1+
EP73246 (+) Hs C6ORF37
EP74124 (+) Hs VBP1
EP73249 (+) Hs FLJ10509
EP73832 (+) Hs GM2A
EP73300 (+) Hs DSTN
EP73372 (+) Hs HBP1
EP07117 (+) Hs Ig k' HK101
EP73843 (+) Hs SMTN
EP73352 (+) Hs C14ORF92
EP73148 (+) Hs INDO
EP74061 (+) Hs RNASEH1
EP74295 (+) Hs CDC2
EP73766 (+) Hs TXNL2
EP73890 (+) Hs URKL1
EP73898 (+) Hs CSTF1
EP73810 (+) Hs COPE
EP74321 (+) Hs POLR2K
EP73623 (+) Hs TARS
EP73929 (+) Hs MRPL42
EP73333 (+) Hs LTA4H
EP73950 (+) Hs ABCE1
EP73913 (+) Hs DFFA
EP73642 (+) Hs BLMH
EP73620 (+) Hs RCN1
EP73632 (+) Hs ASNS
EP40002 (+) Hs complement C5
EP73941 (+) Hs ERH
EP73578 (+) Hs TM4SF2
EP73231 (+) Hs PES1
EP73581 (+) Hs DNAJA1
EP07106 (-) Hs CG/LH/FSH/TSH a'
EP73104 (+) Hs RELA
EP74001 (+) Hs STK25 P2
EP73013 (+) Hs CRYZ
EP73082 (+) Hs SPP1
EP73208 (+) Hs RPL32
EP73597 (+) Hs DEK
EP73114 (+) Hs HSPC117
EP73413 (+) Hs PPP5C
EP73851 (+) Hs ATP5E
EP74066 (+) Hs MRPS16
EP73025 (+) Hs MGC2714
EP73381 (+) Hs GNPAT
EP73076 (+) Hs LOC81501
EP73121 (+) Hs ACTG2
EP73858 (+) Hs ITGB2
EP73259 (+) Hs DAB2
EP73861 (+) Hs PP1201
EP73672 (+) Hs SNRPB
EP73038 (+) Hs DDX17
EP74267 (+) Hs HSPA9B P1
EP73873 (+) Hs FAM14A
EP73454 (+) Hs CCT7
EP73343 (+) Hs TM4SF13
EP74029 (+) Hs ETFA
EP73796 (+) Hs SRP54
EP73031 (+) Hs C14ORF2
EP73350 (+) Hs HSU15552
EP73912 (+) Hs CSRP2
EP73173 (+) Hs SAP18
EP73517 (+) Hs HADHSC
EP14062 (-) Hs IFN-a'4b
EP73792 (+) Hs SPOP
EP73460 (+) Hs ARPC5
EP73508 (+) Hs ANXA4
EP74461 (+) Hs NOLC1
EP74135 (+) Hs YME1L1
EP73171 (+) Hs LUC7A
EP74356 (+) Hs BAG1
EP74173 (+) Hs ART3
EP73176 (+) Hs SPUVE
EP74475 (+) Hs UROS
EP73479 (+) Hs CEPT1
EP73480 (+) Hs MAT2A
EP73499 (+) Hs CAPZB
EP73190 (+) Hs SERPINA3
EP73599 (+) Hs UQCRC1

EP74036 (+) Hs IMPA1
EP73049 (+) Hs B4GALT4
EP74078 (+) Hs RPS9 P2+
EP73537 (+) Hs UXT
EP73675 (+) Hs SRP14
EP73530 (+) Hs AQP3
EP73057 (+) Hs TPM1
EP74037 (+) Hs AK2
EP73888 (+) Hs C20ORF43
EP74289 (+) Hs SEPP1
EP73434 (+) Hs CTGF
EP73071 (+) Hs RPL34
EP26018 (+) Hs ALDOA E4P4
EP73187 (+) Hs PON2
EP74166 (+) Hs ARL1
EP73268 (+) Hs UQCRB
EP73087 (+) Hs DREV1
EP73586 (+) Hs APOL1
EP60004 (+) Hs link
EP73652 (+) Hs GDI2
EP73572 (+) Hs DRG1
EP73133 (+) Hs RPS6
EP74152 (+) Hs BET1
EP74281 (+) Hs PLDN
EP73376 (+) Hs MYCBP
EP73937 (+) Hs RFC3
EP73466 (+) Hs SDCCAG10
EP74585 (+) Hs NDUFA5
EP73438 (+) Hs NFE2L2
EP74556 (+) Hs UBL1
EP73074 (+) Hs DDX5
EP74491 (+) Hs ATP5O
EP73709 (+) Hs FKBP3
EP73659 (+) Hs ENO3
EP73276 (+) Hs FLJ20420
EP73842 (+) Hs TES
EP74597 (+) Hs ARF6
EP73308 (+) Hs FKBP1A
EP73596 (+) Hs SLC7A7
EP73256 (+) Hs HMT
EP30003 (+) Hs c-N-ras
EP73946 (+) Hs HEXA
EP73701 (+) Hs PIP
EP74457 (+) Hs TFAM
EP73270 (+) Hs RSU1
EP73007 (+) Hs IF
EP74547 (+) Hs HMGB2
EP73550 (+) Hs EI24
EP74077 (+) Hs RPS9 P1
EP73363 (+) Hs SMT3H1
EP73180 (+) Hs FLJ11730
EP73914 (+) Hs SNRPC
EP40004 (+) Hs C4BP b' A19 mRNA
EP73918 (+) Hs ATP6V0D1
EP74337 (+) Hs COPS7A P1
EP73078 (+) Hs ITM2C
EP11146 (+) Hs c-myc P1
EP73928 (+) Hs RBM14
EP73857 (+) Hs FH
EP73869 (+) Hs SNW1
EP73673 (+) Hs SPARC
EP73605 (+) Hs ASNA1
EP73677 (+) Hs STATH
EP73496 (+) Hs VAMP3
EP74172 (+) Hs HNRPA1
EP73313 (+) Hs LR8
EP74432 (+) Hs MOCS2
EP73416 (+) Hs SPTLC1
EP73532 (+) Hs GATM
EP73024 (+) Hs DKFZp564K142
EP73838 (+) Hs CDC42EP2
EP74485 (+) Hs IER3
EP73758 (+) Hs HDAC2
EP74476 (+) Hs STIP1
EP74498 (+) Hs GYS1
EP73117 (+) Hs AKR1C3
EP73316 (+) Hs LOC51064
EP74588 (+) Hs NFYB
EP74362 (+) Hs ETV5
EP74129 (+) Hs CHEK1
EP73545 (+) Hs SURB7
EP73594 (+) Hs NRP1
EP73229 (+) Hs CG005
EP26016 (+) Hs ALDOA E3P2
EP73877 (+) Hs EIF3S3
EP73212 (+) Hs HNOEL
EP48006 (+) Hs IRF-2
EP73108 (+) Hs CD44
EP74427 (+) Hs MRPS6
EP73729 (+) Hs SUPT4H1
EP73708 (+) Hs ACTA2
EP73995 (+) Hs EIF1AY
EP73690 (+) Hs UBE2N

AGTTATTT

EP74604 (+) Hs HLA-DPA1
EP74605 (+) Hs PMSCL1 P1
EP74606 (+) Hs PMSCL1 P2+
EP74610 (+) Hs PMF1
EP74613 (+) Hs CPSF5
EP74621 (+) Hs ATF5
EP74622 (+) Hs PDHB
EP74628 (+) Hs LDHC
EP74630 (+) Hs VAPA
EP73708 (+) Hs ACTA2
EP39001 (+) Hs ARF1
EP56002 (+) Hs glycophorin A
EP56003 (-) Hs glycophorin B
EP73978 (+) Hs NUTF2
EP73733 (+) Hs CCND3
EP15044 (+) Hs EGF receptor P2
EP15046 (+) Hs c-erbB2/neu P2+
EP15045 (+) Hs c-erbB2/neu P1
EP73755 (+) Hs PLEK
EP73758 (+) Hs HDAC2
EP74012 (+) Hs DAP3
EP73517 (+) Hs HADHSC
EP74021 (+) Hs CDK5
EP74023 (+) Hs LUC7L
EP74034 (+) Hs SKP1A
EP74041 (+) Hs NOC4
EP74047 (+) Hs RAC1
EP26030 (+) Hs c-abl 7 kb E1P1
EP48006 (+) Hs IRF-2
EP74059 (+) Hs RPL8 P2+
EP74060 (+) Hs CTNNBL1
EP17091 (+) Hs SERPINA1 E1P1+
EP73827 (+) Hs DC6
EP74085 (+) Hs HNRPC
EP74089 (+) Hs MRPL15
EP74091 (+) Hs SERPINB1
EP74096 (+) Hs PP
EP74101 (+) Hs RPL24
EP74106 (+) Hs PAH
EP74113 (+) Hs TARDBP
EP74114 (+) Hs MAD2L2
EP74124 (+) Hs VBP1
EP40002 (+) Hs complement C5
EP74129 (+) Hs CHEK1
EP74134 (+) Hs UMPS
EP74139 (+) Hs ANGPTL3
EP74143 (+) Hs TUBG1
EP73021 (+) Hs NRBP
EP74159 (+) Hs RAB11B
EP74158 (+) Hs RACGAP1
EP73034 (+) Hs GBAS
EP74166 (+) Hs ARL1
EP73050 (+) Hs CDC10
EP73054 (+) Hs HNRPH2
EP74187 (+) Hs PLGL
EP73933 (+) Hs RAD51C
EP74189 (+) Hs GSTZ1
EP73062 (+) Hs ACTR1A
EP74193 (+) Hs APG12L
EP14061 (-) Hs IFN-a'16
EP73945 (+) Hs S100A10
EP74201 (+) Hs PLAT
EP14063 (+) Hs IL-1a'
EP74203 (+) Hs MRPS18B
EP74204 (+) Hs SURF6
EP74206 (+) Hs EEF1B2 P1
EP74207 (+) Hs EEF1B2 P2+
EP73957 (+) Hs APOH
EP74213 (+) Hs APLP2
EP74594 (+) Hs CTSL2
EP74215 (+) Hs SEPX1
EP74216 (+) Hs MRPL4 P1
EP74217 (+) Hs MRPL4 P2+
EP74218 (+) Hs HNRPH3
EP74220 (+) Hs BCCIP
EP73719 (+) Hs RGS2
EP74223 (+) Hs CRSP9
EP74225 (+) Hs EIF3S4
EP74224 (+) Hs RBX1
EP74226 (+) Hs PLTP
EP74326 (+) Hs FBXO22
EP73103 (+) Hs CBARA1
EP74236 (+) Hs SSR3
EP73737 (+) Hs H2AFZ
EP74245 (+) Hs BCL2L12
EP74246 (+) Hs DDX19
EP73119 (+) Hs TF
EP74251 (+) Hs GARS P1
EP73124 (+) Hs ENSA
EP74253 (+) Hs SRP19
EP73128 (+) Hs ADAM15
EP73134 (+) Hs FABP5
EP74268 (+) Hs HSPA9B P2+
EP74271 (+) Hs HLA DRB3
EP73144 (+) Hs CDC5L
EP49001 (+) Hs histone H1t
EP73147 (+) Hs MASP1
EP74281 (+) Hs PLDN
EP74283 (+) Hs COX5A
EP74284 (+) Hs TIMM9
EP74287 (+) Hs NACA
EP74288 (+) Hs CKN1
EP73156 (+) Hs RTN4
EP74292 (+) Hs MRPL13
EP11070 (+) Hs histone H2B
EP26031 (-) Hs c-abl 7 kb E1P2
EP73170 (+) Hs ANLN
EP73516 (+) Hs H3F3B
EP73173 (+) Hs SAP18
EP74315 (+) Hs RPL29
EP73187 (+) Hs PON2
EP73190 (+) Hs SERPINA3
EP74325 (+) Hs DUT
EP74327 (+) Hs MAGEA3
EP74337 (+) Hs COPS7A P1
EP33023 (+) Hs TNP1
EP74340 (+) Hs GUK1
EP73210 (+) Hs SLC31A1
EP74344 (+) Hs ARL4 P1
EP74345 (+) Hs ARL4 P2+
EP74347 (+) Hs MYOZ2 P1
EP74348 (+) Hs MYOZ2 P2+
EP74350 (+) Hs AP1M1
EP73218 (+) Hs PPP2R4
EP74353 (+) Hs BUB3
EP74355 (+) Hs MRPS36
EP41008 (+) Hs snRNP E
EP74357 (+) Hs MRPL37
EP74358 (+) Hs ZWINT
EP74359 (+) Hs RBM4
EP74374 (+) Hs MRPL16
EP73241 (+) Hs HIMAP4
EP74382 (+) Hs BZW2 P2+
EP73252 (+) Hs TMEM30A
EP74387 (+) Hs TAX1BP1
EP73256 (+) Hs HMT
EP74395 (+) Hs MCM2
EP74414 (+) Hs MAP2K1IP1
EP74416 (+) Hs JTB
EP74420 (+) Hs GABARAPL2 P2+
EP73289 (+) Hs YPEL5
EP73293 (+) Hs POLE3
EP73297 (+) Hs ABCB8
EP24025 (-) Hs a'2(I) collagen
EP15029 (+) Hs b'-fibrinogen
EP74450 (+) Hs PSMD2
EP74451 (+) Hs NDUFA1
EP16037 (+) Hs involucrin
EP47002 (+) Hs a'_2 integrin

EP74459 (+) Hs H2AFY
EP74460 (+) Hs TCTE1L
EP73330 (+) Hs C14ORF129
EP74464 (+) Hs HSPA5
EP74465 (+) Hs DAZAP2
EP73337 (+) Hs RRP4
EP73087 (+) Hs DREV1
EP26028 (+) Hs apolipop. B
EP73095 (+) Hs MEP50
EP74480 (+) Hs CRI1
EP73347 (+) Hs PTD004
EP16042 (+) Hs ALB
EP74488 (+) Hs ECHS1
EP16046 (-) Hs a'-fetoprotein
EP73111 (+) Hs RNF2
EP74496 (+) Hs GABARAP P1
EP74502 (+) Hs SNRPG
EP14047 (+) Hs PRBP
EP74511 (+) Hs HARS
EP74521 (+) Hs JUN
EP74531 (+) Hs MRPL3 P2+
EP74533 (+) Hs MEST
EP73746 (+) Hs MCM3
EP73405 (+) Hs PWP1
EP74542 (+) Hs SNRPA1
EP74547 (+) Hs HMGB2
EP74551 (+) Hs HNMT
EP57008 (+) Hs FBP
EP74558 (+) Hs SDCBP
EP74563 (+) Hs HPRT1 P1

EP74564 (+) Hs HPRT1 P2+
EP11105 (-) Hs d'-globin
EP73436 (+) Hs MFAP1
EP74572 (+) Hs PSMB3
EP74581 (+) Hs GNG11
EP11110 (-) Hs e'-globin
EP74585 (+) Hs NDUFA5
EP73450 (+) Hs SLC35A1
EP35012 (+) Hs P-glycoprotein 1
EP60011 (+) Hs Cyclin D1
EP74597 (+) Hs ARF6
EP74601 (+) Hs PGRMC1
EP07056 (+) Hs DHFR
EP73474 (+) Hs SERF2
EP74105 (+) Hs PRKRA
EP30054 (+) Hs arginase liver
EP73239 (+) Hs C20ORF44
EP42001 (+) Hs BCKDHA
EP73498 (+) Hs PLS3
EP56004 (-) Hs glycophorin E
EP73505 (+) Hs RBM3
EP73508 (+) Hs ANXA4
EP73518 (+) Hs DNAJB6
EP73277 (+) Hs FLJ20424
EP73533 (+) Hs ANXA1
EP74419 (+) Hs GABARAPL2 P1
EP73540 (+) Hs CD83
EP73543 (+) Hs TM9SF2
EP73545 (+) Hs SURB7
EP73546 (+) Hs USP49

EP73298 (+) Hs ACAT2
EP16056 (-) Hs renin
EP15034 (+) Hs collagenase
EP74455 (+) Hs RNPS1
EP73041 (+) Hs PEF
EP73578 (+) Hs TM4SF2
EP73582 (+) Hs EIF3S6
EP73586 (+) Hs APOL1
EP35073 (-) Hs P450 XVIIA1 CYP17
EP73596 (+) Hs SLC7A7
EP73612 (+) Hs MDM4
EP73616 (+) Hs PHB
EP74503 (+) Hs HLA-DMA
EP73619 (+) Hs RBMX
EP16058 (+) Hs somatostatin I
EP73625 (+) Hs UGDH
EP73626 (+) Hs ZNF134
EP73627 (+) Hs PTP4A1
EP73531 (+) Hs G22P1
EP14056 (+) Hs prolactin
EP73743 (+) Hs COX6B
EP74535 (+) Hs PSMC6
EP30043 (-) Hs PTH P1+
EP73751 (+) Hs NDUFB6
EP73416 (+) Hs SPTLC1
EP17071 (+) Hs IGF II E1P1
EP74311 (+) Hs PSMA3
EP74573 (+) Hs DAD1
EP14060 (-) Hs IFN-a'5
EP27006 (+) Hs Bcl-2 E1P1+

EP73695 (+) Hs NME2
EP14062 (-) Hs IFN-a'4b
EP11134 (-) Hs IFN l'2c1 (-a'17)
EP07111 (+) Hs LeIF-J (IFN-a'7)
EP07112 (-) Hs b'-interferon
EP73699 (+) Hs PFN2
EP07113 (+) Hs g'-interferon
EP74396 (+) Hs IMPA2
EP73709 (+) Hs FKBP3
EP25044 (-) Hs IL-5 (EDF/TRF)
EP17080 (+) Hs IL-6 (BSF-2) P1
EP17081 (+) Hs IL-6 (BSF-2) P2+
EP73811 (+) Hs HPCL2
EP74066 (+) Hs MRPS16
EP73842 (+) Hs TES
EP73732 (+) Hs CAPG
EP74616 (+) Hs COIL
EP17092 (+) Hs SERPINA1 E2P2
EP73485 (+) Hs BNIP3L
EP30056 (+) Hs OAT
EP73106 (+) Hs ZFP36L1
EP73749 (+) Hs NDUFA4
EP73225 (+) Hs RISC
EP73765 (+) Hs PKIA
EP07121 (+) Hs MHCII HLA-DRA
EP73777 (+) Hs FLJ20920
EP73869 (+) Hs SNW1
EP30017 (+) Hs PGK1
EP73782 (+) Hs HADHA
EP40003 (+) Hs DAF

EP73689 (+) Hs UBE2L3
EP30003 (+) Hs c-N-ras
EP26032 (+) Hs c-abl 6 kb E2P3
EP73263 (+) Hs JWA
EP73801 (+) Hs RDHL
EP74432 (+) Hs MOCS2
EP70003 (+) Hs CXCL14
EP25036 (+) Hs MT-IB
EP73030 (+) Hs MGC14156
EP73567 (+) Hs GYG
EP73820 (+) Hs CKLFSF6
EP73037 (+) Hs HBXIP
EP73037 (+) Hs LOC51705
EP73574 (+) Hs PET112L
EP73829 (+) Hs ALG2
EP73832 (+) Hs GM2A
EP73584 (+) Hs DCTD
EP73834 (+) Hs LYZ
EP73839 (+) Hs HNRPA3
EP73850 (+) Hs SDBCAG84
EP73324 (+) Hs VRK3
EP73859 (+) Hs LPL
EP73861 (+) Hs PP1201
EP40004 (+) Hs C4BP b' A19 mRNA
EP73880 (+) Hs RPL35
EP73006 (+) Hs CAV1
EP73007 (+) Hs IF
EP73884 (+) Hs LIPA
EP73352 (+) Hs C14ORF92
EP73012 (+) Hs MGC3248

EP73017 (+) Hs UNRIP
EP73901 (+) Hs PPP1R12A
EP73025 (+) Hs MGC2714
EP73026 (+) Hs MGC11061
EP73143 (+) Hs NQO1
EP73029 (+) Hs FLJ14904
EP73912 (+) Hs CSRP2
EP73038 (+) Hs DDX17
EP73131 (+) Hs TFG
EP74264 (+) Hs PSCD2
EP11113 (+) Hs ADA
EP73051 (+) Hs PDLIM1
EP73929 (+) Hs MRPL42
EP73928 (+) Hs RBM14
EP73057 (+) Hs TPM1
EP73400 (+) Hs TOMM34
EP14077 (+) Hs factor VIII
EP73175 (+) Hs PXMP3
EP11135 (-) Hs IFN l'2h (-a'14)
EP73408 (+) Hs BTG1
EP73942 (+) Hs TSG101
EP73696 (+) Hs OMG
EP73944 (+) Hs IGFBP7
EP73072 (+) Hs WDR23
EP74300 (+) Hs GABARAPL1
EP73076 (+) Hs LOC81501
EP73953 (+) Hs ELAC2
EP73707 (+) Hs C1S
EP74534 (+) Hs UBE2V2
EP73423 (+) Hs CCT8

EP73081 (+) Hs KIF22
EP73425 (+) Hs PC4
EP73712 (+) Hs PRPS1
EP73772 (+) Hs FLJ13188
EP73964 (+) Hs TBPL1
EP73965 (+) Hs RNF7
EP73434 (+) Hs CTGF
EP73094 (+) Hs HDAC1
EP73185 (+) Hs CLU
EP33035 (+) Hs CNTF
EP74125 (+) Hs WRB P1
EP73875 (+) Hs SMARCE1
EP73976 (+) Hs XBP1
EP31009 (+) Hs HMG-17
EP73113 (+) Hs MKLN1
EP73117 (+) Hs AKR1C3
EP73459 (+) Hs CTNNA1
EP74145 (+) Hs ANXA2
EP60012 (+) Hs a'-1a-AdrenR
EP73752 (+) Hs NDUFS4
EP57007 (+) Hs IL-8 receptor B
EP73467 (+) Hs LRRC17
EP74003 (+) Hs PRPF18
EP73130 (+) Hs CRACC
EP74099 (+) Hs CDK7 P2+
EP73471 (+) Hs FRSB
EP73759 (+) Hs NME1
EP73135 (+) Hs CDW52
EP73762 (+) Hs C3ORF4
EP73478 (+) Hs SLC35A2

EP73480 (+) Hs MAT2A
EP73141 (+) Hs MRPL44
EP73482 (+) Hs CSE1L
EP74020 (+) Hs CSNK2B
EP73145 (+) Hs ST13
EP73774 (+) Hs MLPH
EP73148 (+) Hs INDO
EP74025 (+) Hs SDHD
EP73152 (+) Hs NDUFA9
EP73151 (+) Hs C5orf13
EP74031 (+) Hs GOT2
EP74381 (+) Hs BZW2 P1
EP73500 (+) Hs CCNC
EP74036 (+) Hs IMPA1
EP73160 (+) Hs CKM
EP73161 (+) Hs PIGPC1
EP73795 (+) Hs SP100
EP73169 (+) Hs D21S2056E
EP73797 (+) Hs ATIC
EP73800 (+) Hs PSMD14
EP74398 (+) Hs PELO P1
EP74400 (+) Hs TIMP1
EP73178 (+) Hs PLOD3
EP74169 (+) Hs TCL1A
EP74058 (+) Hs RPL8 P1
EP73183 (+) Hs RPS4X
EP73527 (+) Hs LGMN
EP73189 (+) Hs SERPINB2
EP73191 (+) Hs ATP6V1C1
EP73192 (+) Hs SERPIND1

EP73193 (+) Hs RGS4
EP26019 (+) Hs neurofilament L
EP74074 (+) Hs IFNGR1
EP73632 (+) Hs ASNS
EP25041 (+) Hs a'1(III) collagen
EP73201 (+) Hs RIC
EP73443 (+) Hs CCT3
EP73203 (+) Hs PSAP
EP74082 (+) Hs NARS
EP73300 (+) Hs DSTN
EP73258 (+) Hs RPL37A
EP73763 (+) Hs TACSTD1
EP73215 (+) Hs NFKBIA
EP73216 (+) Hs MYL6
EP73333 (+) Hs LTA4H
EP73560 (+) Hs B2M
EP73564 (+) Hs STOM
EP73223 (+) Hs SQRDL
EP73231 (+) Hs PES1
EP73551 (+) Hs PRDX6
EP73132 (+) Hs IL10RB
EP73325 (+) Hs PAIP2
EP74458 (+) Hs IDH3G
EP73862 (+) Hs TGOLN2
EP74115 (+) Hs ZNF265
EP73864 (+) Hs CYP3A5
EP74119 (+) Hs HLA DQA1
EP74120 (+) Hs NDUFA10
EP73335 (+) Hs COX7C
EP73249 (+) Hs FLJ10509

EP73149 (+) Hs RAB1A
EP73877 (+) Hs EIF3S3
EP17030 (-) Hs snRNA U1 (pU1-6)
EP73154 (+) Hs RPL10
EP73255 (+) Hs BAP29
EP74457 (+) Hs TFAM
EP73259 (+) Hs DAB2
EP73014 (+) Hs LMCD1
EP73260 (+) Hs COX4I1
EP73887 (+) Hs RDH11
EP74140 (+) Hs DNAJA2
EP74237 (+) Hs RPL35A P1
EP73015 (+) Hs RPS11
EP26008 (+) Hs PBGD E E2P2
EP73019 (+) Hs SSBP1
EP73896 (+) Hs C3F
EP73857 (+) Hs FH
EP74152 (+) Hs BET1
EP74153 (+) Hs SKP2
EP74154 (+) Hs TIMM13
EP73027 (+) Hs MGC15429
EP73963 (+) Hs CAMLG
EP73280 (+) Hs FLJ10276
EP73281 (+) Hs FANCL
EP73716 (+) Hs PSMC1
EP74160 (+) Hs YARS
EP73910 (+) Hs CLTA
EP48007 (+) Hs TNNI1
EP74413 (+) Hs PCBP2
EP73382 (+) Hs PNLIP

EP73294 (+) Hs SLC38A2
EP73043 (+) Hs ADH1C
EP73301 (+) Hs AF1Q
EP73870 (+) Hs DKFZP586F1524
EP74295 (+) Hs CDC2
EP73392 (+) Hs CALM1
EP74181 (+) Hs ELOVL1
EP73646 (+) Hs CPA2
EP73055 (+) Hs HNRPR
EP73208 (+) Hs RPL32
EP73310 (+) Hs COX7B
EP73060 (+) Hs PLOD
EP73061 (+) Hs ITM2B
EP73315 (+) Hs OVCOV1
EP64001 (+) Hs rag-1
EP73221 (+) Hs PSA1
EP74431 (+) Hs SUCLG1
EP73372 (+) Hs HBP1
EP73075 (+) Hs DUSP11
EP73647 (+) Hs CPA3
EP74555 (+) Hs HLA-DPB1
EP73878 (+) Hs AP1S2
EP73331 (+) Hs HSPC251
EP74559 (+) Hs GYPC
EP74022 (+) Hs SFRS5
EP73340 (+) Hs E2IG3
EP73343 (+) Hs TM4SF13
EP73016 (+) Hs RPS12
EP73346 (+) Hs DDX25
EP73247 (+) Hs FLJ20274
EP73692 (+) Hs LGALS3
EP73897 (+) Hs DDX39
EP73354 (+) Hs KIAA0971
EP73355 (+) Hs CGI51
EP73357 (+) Hs MGP
EP73704 (+) Hs PRG1
EP74243 (+) Hs PDCD10
EP73365 (+) Hs D1S155E
EP73573 (+) Hs SFRS10
EP74249 (+) Hs PPP4C
EP73576 (+) Hs RAB5A
EP74483 (+) Hs TMSB4X
EP73371 (+) Hs CHORDC1
EP74600 (+) Hs STMN2
EP14030 (+) Hs a'-tubulin ba'1
EP74505 (+) Hs HMGN3
EP73494 (+) Hs CCNB2
EP73727 (+) Hs SNX1
EP73479 (+) Hs CEPT1
EP74087 (+) Hs GOLPH3
EP73150 (+) Hs COX7A2L
EP73642 (+) Hs BLMH
EP73724 (+) Hs SH3BGRL
EP73453 (+) Hs HMGN4
EP74338 (+) Hs COPS7A P2+
EP73753 (+) Hs NDUFS8
EP74442 (+) Hs RPL15
EP73412 (+) Hs POLD2
EP73641 (+) Hs CXCL6
EP73542 (+) Hs TXNL
EP73200 (+) Hs NR1H2
EP74104 (+) Hs ACPP
EP73469 (+) Hs PBEF
EP74109 (+) Hs DYT1
EP74399 (+) Hs PELO P2+
EP74309 (+) Hs RPS15A
EP73213 (+) Hs TOMM22
EP73672 (+) Hs SNRPB
EP73430 (+) Hs TFPI2
EP73776 (+) Hs PCNT1
EP73661 (+) Hs PRSS1
EP74238 (+) Hs RPL35A P2+
EP73186 (+) Hs PRKCABP
EP41007 (+) Hs rp S17
EP73439 (+) Hs NNMT
EP73895 (+) Hs RNF13
EP73114 (+) Hs HSPC117
EP74476 (+) Hs STIP1
EP74556 (+) Hs UBL1
EP11074 (+) Hs histone H4-A1
EP74136 (+) Hs TIMM17A
EP73009 (+) Hs GJA1
EP74308 (+) Hs RPL36
EP74341 (+) Hs UP
EP73522 (+) Hs HLA-B
EP73893 (+) Hs PHGDH
EP73556 (+) Hs HIF1A
EP74157 (+) Hs HIBCH
EP73906 (+) Hs PGLS
EP73569 (+) Hs NAP1L1
EP73376 (+) Hs MYCBP
EP74593 (+) Hs SNRPD1
EP73188 (+) Hs SERPINH1
EP74596 (+) Hs ATP5G2
EP74517 (+) Hs DHX38
EP73536 (+) Hs UCHL1
EP74487 (+) Hs BAD
EP73492 (+) Hs ILK
EP73490 (+) Hs GBP2
EP73495 (+) Hs SCYE1
EP73246 (+) Hs C6ORF37
EP73396 (+) Hs RIPK3
EP73841 (+) Hs NAPA
EP73890 (+) Hs URKL1
EP23004 (+) Hs ALDH_2 mit
EP73745 (+) Hs KPNB3
EP74434 (+) Hs ZNF259
EP73847 (+) Hs RPS27
EP73898 (+) Hs CSTF1
EP74543 (+) Hs NDUFS5
EP73510 (+) Hs RAD23A
EP73853 (+) Hs CBX3
EP74394 (+) Hs TNRC5
EP73070 (+) Hs APOL3
EP73608 (+) Hs AHCY
EP74493 (+) Hs RPS3A
EP73496 (+) Hs VAMP3
EP14057 (+) Hs thyroglobulin
EP73272 (+) Hs SC4MOL
EP73524 (+) Hs IFI27
EP49012 (-) Hs histone H4t
EP74407 (+) Hs PRKAB1
EP73982 (+) Hs MTAP
EP74510 (+) Hs KNG
EP73872 (+) Hs DC50
EP74415 (+) Hs HTATIP
EP73587 (+) Hs TCAP
EP73435 (+) Hs MDH1
EP74513 (+) Hs PPIG
EP07109 (+) Hs insulin
EP73655 (+) Hs LGALS1
EP73100 (+) Hs KAI1
EP73406 (+) Hs SLC25A5
EP74427 (+) Hs MRPS6
EP73547 (+) Hs PMPCB
EP45003 (+) Hs endothelin-B rec.
EP73107 (+) Hs IGFBP3
EP73321 (+) Hs LOC51125
EP48005 (+) Hs VLDL rec.
EP17050 (+) Hs vWf
EP73798 (+) Hs CIR
EP73812 (+) Hs HSPBP1
EP24040 (+) Hs rp S14
EP73900 (+) Hs MAP1LC3B
EP73904 (+) Hs RPL18
EP73541 (+) Hs TRIP15
EP74252 (+) Hs GARS P2+
EP73729 (+) Hs SUPT4H1
EP73317 (+) Hs RPS27L
EP74364 (+) Hs RPL28

EP73911 (+) Hs ENC1
EP74453 (+) Hs ARHG
EP73407 (+) Hs ARHGDIB
EP74609 (+) Hs EIF4B
EP73395 (+) Hs OS
EP73039 (+) Hs DDX18
EP74497 (+) Hs GABARAP P2+
EP73329 (+) Hs C20ORF111
EP73787 (+) Hs PNN
EP73698 (+) Hs PBP
EP73534 (+) Hs CTSC
EP74561 (+) Hs SDC1
EP73583 (+) Hs AMD1
EP73452 (+) Hs DNAJB1
EP73930 (+) Hs IDH1
EP73833 (+) Hs IRF3
EP73198 (+) Hs HLA C
EP73153 (+) Hs NDUFS1
EP73919 (+) Hs BYSL
EP14058 (-) Hs IFN-a'13
EP49022 (+) Hs lck P2
EP73462 (+) Hs ARPC1B
EP73790 (+) Hs PSMD11
EP73831 (+) Hs GLA
EP73353 (+) Hs STAF65
EP74178 (+) Hs RTN3
EP74584 (+) Hs CKS2
EP73610 (+) Hs M6PR
EP73852 (+) Hs ATP6V1D
EP73614 (+) Hs MTIF2

EP74614 (+) Hs TSNAX
EP74354 (+) Hs SERPINB6
EP73579 (+) Hs TM4SF3
EP73415 (+) Hs ARPC1A
EP73937 (+) Hs RFC3
EP73521 (+) Hs SCARB2
EP74013 (+) Hs VARS2
EP73181 (+) Hs FXR1
EP73725 (+) Hs SMARCB1
EP30074 (-) Hs FSH b'
EP73336 (+) Hs IMAGE145052
EP74321 (+) Hs POLR2K
EP30071 (+) Hs TSH b'
EP73684 (+) Hs EPHX2
EP73240 (+) Hs FLJ11000
EP74519 (+) Hs EIF3S5
EP73387 (+) Hs SFRS7
EP73504 (+) Hs BNIP3
EP27007 (+) Hs Bcl-2 E2P2
EP73410 (+) Hs OSF
EP73069 (+) Hs CDK5RAP3
EP73351 (+) Hs KIAA0102
EP73319 (+) Hs ZDHHC9
EP73949 (+) Hs HSPE1
EP73397 (+) Hs RNP24
EP73993 (+) Hs HRB2
EP73653 (+) Hs GOT1
EP74392 (+) Hs RAB6A
EP73603 (+) Hs P4HA2
EP74436 (+) Hs AUTL1

EP73750 (+) Hs NDUFA6
EP73525 (+) Hs MEP1A
EP74142 (+) Hs RFX4
EP73105 (+) Hs LGTN
EP73219 (+) Hs RPL36A
EP73433 (+) Hs ATF4
EP73891 (+) Hs CMAS
EP74126 (+) Hs WRB P2+
EP73276 (+) Hs FLJ20420
EP74177 (+) Hs PTTG1
EP73229 (+) Hs CG005
EP73165 (+) Hs RAB18
EP74557 (+) Hs DECR1
EP74595 (+) Hs SORD
EP74018 (+) Hs AATF
EP73818 (+) Hs C14ORF100
EP73920 (+) Hs MRPS22
EP73238 (+) Hs FLJ10525
EP73845 (+) Hs ATP6IP2
EP73924 (+) Hs HN1
EP73688 (+) Hs UBE2D3
EP73770 (+) Hs SSB
EP74173 (+) Hs ART3
EP73791 (+) Hs ZNF207
EP73886 (+) Hs CRYL1
EP73809 (+) Hs MYL2
EP73697 (+) Hs PRDX1
EP74486 (+) Hs DCN
EP73597 (+) Hs DEK
EP73466 (+) Hs SDCCAG10

EP73760 (+) Hs AHSA1
EP73794 (+) Hs ATP5G3
EP73537 (+) Hs UXT
EP73036 (+) Hs SH3BGRL3
EP74449 (+) Hs DEAH
EP74590 (+) Hs WDR1
EP73754 (+) Hs ORC4L
EP74267 (+) Hs HSPA9B P1
EP30044 (-) Hs PTH P2
EP74538 (+) Hs YWHAE
EP11104 (+) Hs b'-globin
EP73369 (+) Hs COG2
EP74019 (+) Hs CALR
EP74602 (+) Hs ADRM1
EP73907 (+) Hs RBM8A
EP25085 (-) Hs opsin red
EP74273 (+) Hs PMVK
EP73640 (+) Hs UBE2A
EP74067 (+) Hs RPS24
EP74530 (+) Hs MRPL3 P1
EP74151 (+) Hs BCAS2
EP73284 (+) Hs CDC20
EP74093 (+) Hs TDO2
EP73286 (+) Hs PIGT
EP74468 (+) Hs HSD17B4
EP74569 (+) Hs LAMP2
EP74098 (+) Hs CDK7 P1
EP73826 (+) Hs ACTR10
EP68001 (-) Hs CA2
EP73670 (+) Hs SEMG2

EP74389 (+) Hs BAT1
EP73118 (+) Hs ADH5
EP73837 (+) Hs IDI1
EP73675 (+) Hs SRP14
EP73744 (+) Hs KPNB2
EP73607 (+) Hs APEX1
EP73285 (+) Hs COX7A2
EP73628 (+) Hs PDHX
EP73796 (+) Hs SRP54
EP73958 (+) Hs TIMM8A
EP73694 (+) Hs NASP
EP73652 (+) Hs GDI2
EP73390 (+) Hs MGST1
EP73643 (+) Hs CNN3
EP73657 (+) Hs ATP6V1E1
EP73902 (+) Hs RPL10A
EP74440 (+) Hs SFRS2
EP73851 (+) Hs ATP5E
EP11141 (+) Hs estrogen receptor
EP73403 (+) Hs KIF3A
EP74049 (+) Hs EIF4A2
EP73180 (+) Hs FLJ11730
EP73658 (+) Hs ADFP
EP73032 (+) Hs PLP1
EP73980 (+) Hs MPHOSPH6
EP74117 (+) Hs APCS
EP73228 (+) Hs CCT2
EP73624 (+) Hs TCF12
EP73806 (+) Hs GPI
EP73950 (+) Hs ABCE1

EP73066 (+) Hs MMS19L

EP73581 (+) Hs DNAJA1

EP73222 (+) Hs LOC57862

EP73146 (+) Hs ATP5J

EP73741 (+) Hs HTN1

EP74418 (+) Hs AASDHPPT

EP74135 (+) Hs YME1L1

EP73778 (+) Hs RAB7

EP73108 (+) Hs CD44

EP73764 (+) Hs RAD18

EP73593 (+) Hs SNAP23

EP73637 (+) Hs CRISP2

EP73318 (+) Hs ADIPOR1

EP74463 (+) Hs CPE

EP73167 (+) Hs FLJ13390

EP73539 (+) Hs PRPF4

EP73299 (+) Hs ACTR3

EP73424 (+) Hs PROL3

EP73349 (+) Hs HSU53209

EP74328 (+) Hs UQCRFS1

EP74565 (+) Hs PROCR

EP73580 (+) Hs TSN

EP73975 (+) Hs VDAC1

EP73269 (+) Hs QPCT

EP73793 (+) Hs RPL14

EP73512 (+) Hs CALM3

EP73706 (+) Hs BPGM

EP73273 (+) Hs SC5DL

EP74313 (+) Hs CSH1

EP73810 (+) Hs COPE

EP73477 (+) Hs TAF11

AGAAATTT

EP74604 (+) Hs HLA-DPA1
EP74606 (+) Hs PMSCL1 P2+
EP74605 (+) Hs PMSCL1 P1
EP74609 (+) Hs EIF4B
EP73933 (+) Hs RAD51C
EP74612 (+) Hs PMAIP1
EP74613 (+) Hs CPSF5
EP74619 (+) Hs PSMD3 P1
EP74620 (+) Hs PSMD3 P2+
EP74622 (+) Hs PDHB
EP74624 (+) Hs NUP62
EP74628 (+) Hs LDHC
EP14077 (+) Hs factor VIII
EP73952 (+) Hs SNX3 P2+
EP73954 (+) Hs WDR13
EP41009 (+) Hs ARF 3
EP56002 (+) Hs glycophorin A
EP56003 (-) Hs glycophorin B
EP56004 (-) Hs glycophorin E
EP15056 (+) Hs E-resp. RNA pS2
EP25021 (+) Hs IFIT1
EP73468 (+) Hs P5
EP27009 (+) Hs IFI 6-16
EP73971 (+) Hs NAT8
EP73977 (+) Hs HGD
EP58019 (+) Hs WT1
EP64001 (+) Hs rag-1
EP15043 (+) Hs EGF receptor P1
EP15044 (+) Hs EGF receptor P2
EP73988 (+) Hs APEX2
EP73995 (+) Hs EIF1AY
EP73998 (+) Hs TAF7
EP48005 (+) Hs VLDL rec.
EP73769 (+) Hs SEPT6
EP73777 (+) Hs FLJ20920
EP74029 (+) Hs ETFA
EP74035 (+) Hs PPP2R5C
EP74038 (+) Hs NUP88
EP74043 (+) Hs SNRPA P2+
EP74049 (+) Hs EIF4A2
EP74053 (+) Hs FAU P1
EP74054 (+) Hs FAU P2+
EP74066 (+) Hs MRPS16
EP74074 (+) Hs IFNGR1
EP74084 (+) Hs UFD1L
EP74085 (+) Hs HNRPC
EP74092 (+) Hs CRABP2
EP74103 (+) Hs TCEB1 P2+
EP74120 (+) Hs NDUFA10
EP74143 (+) Hs TUBG1
EP73040 (+) Hs DKFZP564G2022
EP74173 (+) Hs ART3
EP73928 (+) Hs RBM14
EP74183 (+) Hs NUBP2
EP74185 (+) Hs FTSJ3
EP74187 (+) Hs PLGL
EP73065 (+) Hs MATR3
EP74193 (+) Hs APG12L
EP73943 (+) Hs SMC2L1
EP07112 (-) Hs b'-interferon
EP73071 (+) Hs RPL34
EP73950 (+) Hs ABCE1
EP73705 (+) Hs PRKAR1A
EP74209 (+) Hs KRT17 P1
EP74210 (+) Hs KRT17 P2+
EP74211 (+) Hs HFL1
EP74213 (+) Hs APLP2
EP74215 (+) Hs SEPX1
EP74220 (+) Hs BCCIP
EP73094 (+) Hs HDAC1
EP74224 (+) Hs RBX1
EP74228 (+) Hs STARD7
EP74229 (+) Hs RNASE4
EP11159 (+) Hs TNF-b'
EP74236 (+) Hs SSR3
EP74237 (+) Hs RPL35A P1
EP73990 (+) Hs HFL3
EP74256 (+) Hs ANXA5
EP57007 (+) Hs IL-8 receptor B
EP74259 (+) Hs SPR
EP74260 (+) Hs RPS7 P1
EP74261 (+) Hs RPS7 P2
EP74262 (+) Hs RPS7 P3+
EP74266 (+) Hs CDK5RAP1
EP73140 (+) Hs PFDN5
EP74270 (+) Hs PRAME
EP49001 (+) Hs histone H1t
EP73148 (+) Hs INDO
EP30042 (+) Hs histone H1a
EP74288 (+) Hs CKN1
EP74293 (+) Hs KRT6A
EP73166 (+) Hs LIMR
EP74304 (+) Hs DAXX
EP74307 (+) Hs CKLFSF7
EP74315 (+) Hs RPL29
EP11073 (+) Hs histone H3b
EP73189 (+) Hs SERPINB2
EP73191 (+) Hs ATP6V1C1
EP11074 (+) Hs histone H4-A1
EP31007 (+) Hs HMG-14
EP74336 (+) Hs HLA DRB1
EP33038 (+) Hs PRM2
EP73217 (+) Hs PPIA
EP74352 (+) Hs SIAT7D
EP73229 (+) Hs CG005
EP73230 (+) Hs SERPINA5
EP37014 (+) Hs[rig] rp S15
EP73240 (+) Hs FLJ11000
EP74374 (+) Hs MRPL16
EP73247 (+) Hs FLJ20274
EP74382 (+) Hs BZW2 P2+
EP74390 (+) Hs GGPS1
EP73263 (+) Hs JWA
EP48007 (+) Hs TNNI1
EP74404 (+) Hs U2AF1
EP74407 (+) Hs PRKAB1
EP74417 (+) Hs SDHB
EP26019 (+) Hs neurofilament L
EP74419 (+) Hs GABARAPL2 P1
EP25041 (+) Hs a'1(III) collagen
EP73293 (+) Hs POLE3
EP73295 (+) Hs P17
EP74432 (+) Hs MOCS2
EP60004 (+) Hs link
EP74435 (+) Hs MRPS7
EP74436 (+) Hs AUTL1
EP74439 (+) Hs UQCRH
EP73074 (+) Hs DDX5
EP28010 (+) Hs IGF II E2P2
EP17072 (-) Hs IGF II E3P3
EP74465 (+) Hs DAZAP2
EP26028 (+) Hs apolipop. B
EP73343 (+) Hs TM4SF13
EP74483 (+) Hs TMSB4X
EP16042 (+) Hs ALB
EP74492 (+) Hs ATP1B3
EP74495 (+) Hs ARAF1
EP73360 (+) Hs CSNK1A1
EP74503 (+) Hs HLA-DMA
EP73370 (+) Hs GTF3C5
EP73371 (+) Hs CHORDC1
EP73374 (+) Hs FTSJ1
EP73126 (+) Hs GLO1
EP74512 (+) Hs ERCC3
EP74517 (+) Hs DHX38
EP73385 (+) Hs CSNK2A1
EP73392 (+) Hs CALM1
EP11091 (+) Hs MB
EP74534 (+) Hs UBE2V2

EP74535 (+) Hs PSMC6
EP74539 (+) Hs H2AFO
EP74540 (+) Hs CD79A
EP11098 (-) Hs z'-globin
EP74548 (+) Hs DARS
EP74553 (+) Hs MRPS21
EP74559 (+) Hs GYPC
EP74561 (+) Hs SDC1
EP74563 (+) Hs HPRT1 P1
EP74564 (+) Hs HPRT1 P2+
EP11104 (+) Hs b'-globin
EP11105 (-) Hs d'-globin
EP73439 (+) Hs NNMT
EP74575 (+) Hs CCT6A P2+
EP74577 (+) Hs TYMS P1
EP74579 (+) Hs PEX11B
EP74581 (+) Hs GNG11
EP74583 (+) Hs TNFSF10
EP74592 (+) Hs FADD
EP74593 (+) Hs SNRPD1
EP74594 (+) Hs CTSL2
EP74596 (+) Hs ATP5G2
EP25014 (+) Hs 2'5'-oligoA synt.
EP07056 (+) Hs DHFR
EP73477 (+) Hs TAF11
EP64003 (+) Hs TdT
EP74111 (+) Hs BIN1
EP73235 (+) Hs SLC31A2
EP73496 (+) Hs VAMP3
EP73503 (+) Hs LDHA

EP16050 (+) Hs HMG-CoA red.
EP73524 (+) Hs IFI27
EP11083 (+) Hs keratin 67K
EP73533 (+) Hs ANXA1
EP16071 (+) Hs[ERV3]
EP73537 (+) Hs UXT
EP31010 (+) Hs hepatic lipase
EP73540 (+) Hs CD83
EP73544 (+) Hs SLC22A8
EP73545 (+) Hs SURB7
EP73551 (+) Hs PRDX6
EP73019 (+) Hs SSBP1
EP07095 (+) Hs factor IX
EP73021 (+) Hs NRBP
EP28005 (+) Hs pepsinogen C
EP73564 (+) Hs STOM
EP15034 (+) Hs collagenase
EP73566 (+) Hs GTF2F2
EP48003 (+) Hs collagenaseIV 72K
EP30065 (+) Hs AMY1
EP24034 (-) Hs P450 IA1 +MC
EP11121 (+) Hs P450 IA1 +TCDD
EP23004 (+) Hs ALDH_2 mit
EP73595 (+) Hs EIF2S2
EP68001 (-) Hs CA2
EP30047 (+) Hs CKB
EP73602 (+) Hs YWHAB
EP73608 (+) Hs AHCY
EP73610 (+) Hs M6PR
EP74242 (+) Hs MRPL50

EP29004 (-) Hs calcitonin/a'CGRP
EP17067 (+) Hs glucagon
EP73630 (+) Hs TPST2
EP07106 (-) Hs CG/LH/FSH/TSH a'
EP73636 (+) Hs TPM2
EP49012 (-) Hs histone H4t
EP30074 (-) Hs FSH b'
EP11125 (-) Hs growth hormone
EP73652 (+) Hs GDI2
EP74549 (+) Hs NTS
EP73669 (+) Hs SEMG1
EP73670 (+) Hs SEMG2
EP28009 (-) Hs IGF II E4P4
EP73435 (+) Hs MDH1
EP14059 (-) Hs IFN-a'6
EP74574 (+) Hs CCT6A P1
EP07113 (+) Hs g'-interferon
EP14063 (+) Hs IL-1a'
EP73711 (+) Hs PPP1R7
EP74597 (+) Hs ARF6
EP73719 (+) Hs RGS2
EP11158 (+) Hs TNF-a'
EP74615 (+) Hs PRCC
EP73734 (+) Hs FNTB
EP73738 (+) Hs HLA DRB1
EP73741 (+) Hs HTN1
EP73744 (+) Hs KPNB2
EP25085 (-) Hs opsin red
EP11142 (+) Hs IL-2 receptor P1
EP11143 (+) Hs IL-2 receptor P2

EP15041 (+) Hs transferrin rec.
EP73762 (+) Hs C3ORF4
EP73765 (+) Hs PKIA
EP73522 (+) Hs HLA-B
EP73580 (+) Hs TSN
EP73526 (+) Hs NFIC
EP27010 (+) Hs L-myc
EP27006 (+) Hs Bcl-2 E1P1+
EP73792 (+) Hs SPOP
EP73794 (+) Hs ATP5G3
EP73797 (+) Hs ATIC
EP73548 (+) Hs ITM2A
EP73804 (+) Hs CCT6B
EP71003 (+) Hs RGS1
EP73819 (+) Hs FLJ10458
EP73823 (+) Hs ESRRBL1
EP73828 (+) Hs RPS23
EP73638 (+) Hs TXN
EP07117 (+) Hs Ig k' HK101
EP73842 (+) Hs TES
EP73846 (+) Hs NOLA2
EP73599 (+) Hs UQCRC1
EP73860 (+) Hs CPR2
EP73328 (+) Hs HSPC148
EP14076 (+) Hs CD74
EP73674 (+) Hs SRI
EP73865 (+) Hs DDX24
EP73336 (+) Hs IMAGE145052
EP73962 (+) Hs RPL39L
EP73339 (+) Hs SMP1

EP40002 (+) Hs complement C5
EP40003 (+) Hs DAF
EP40004 (+) Hs C4BP b' A19 mRNA
EP73881 (+) Hs ODC1
EP73008 (+) Hs PDHA1
EP73593 (+) Hs SNAP23
EP73010 (+) Hs CD9
EP73641 (+) Hs CXCL6
EP73363 (+) Hs SMT3H1
EP73901 (+) Hs PPP1R12A
EP73026 (+) Hs MGC11061
EP73030 (+) Hs MGC14156
EP73034 (+) Hs GBAS
EP73035 (+) Hs PCCA
EP73039 (+) Hs DDX18
EP73042 (+) Hs ADH1B
EP73387 (+) Hs SFRS7
EP73925 (+) Hs GADD45B
EP74426 (+) Hs VPS29
EP73927 (+) Hs PTTG1IP
EP73054 (+) Hs HNRPH2
EP73057 (+) Hs TPM1
EP73061 (+) Hs ITM2B
EP73063 (+) Hs ABP1
EP73064 (+) Hs OCIA
EP73410 (+) Hs OSF
EP73944 (+) Hs IGFBP7
EP74040 (+) Hs VAMP8
EP73946 (+) Hs HEXA
EP73073 (+) Hs SID6

EP73949 (+) Hs HSPE1
EP73072 (+) Hs WDR23
EP74589 (+) Hs CLNS1A
EP73082 (+) Hs SPP1
EP73083 (+) Hs IL8
EP73095 (+) Hs MEP50
EP73096 (+) Hs MGC5306
EP73723 (+) Hs SFRS3
EP33035 (+) Hs CNTF
EP73726 (+) Hs SNAP25
EP73727 (+) Hs SNX1
EP73102 (+) Hs CARS
EP73103 (+) Hs CBARA1
EP73879 (+) Hs EIF2S1
EP73108 (+) Hs CD44
EP73112 (+) Hs NONO
EP73113 (+) Hs MKLN1
EP73227 (+) Hs SQSTM1
EP73991 (+) Hs MRPS31
EP73208 (+) Hs RPL32
EP73119 (+) Hs TF
EP73120 (+) Hs CHIT1
EP73749 (+) Hs NDUFA4
EP73751 (+) Hs NDUFB6
EP73754 (+) Hs ORC4L
EP73757 (+) Hs HSD3B2
EP73131 (+) Hs TFG
EP73132 (+) Hs IL10RB
EP73760 (+) Hs AHSA1
EP73820 (+) Hs CKLFSF6
EP73479 (+) Hs CEPT1
EP74018 (+) Hs AATF
EP73146 (+) Hs ATP5J
EP73488 (+) Hs CCNG1
EP73149 (+) Hs RAB1A
EP73150 (+) Hs COX7A2L
EP73155 (+) Hs PSMF1
EP73500 (+) Hs CCNC
EP74036 (+) Hs IMPA1
EP74386 (+) Hs CLDN4
EP73255 (+) Hs BAP29
EP30003 (+) Hs c-N-ras
EP74042 (+) Hs SNRPA P1
EP26030 (+) Hs c-abl 7 kb E1P1
EP73170 (+) Hs ANLN
EP73171 (+) Hs LUC7A
EP73175 (+) Hs PXMP3
EP73177 (+) Hs MKKS
EP73179 (+) Hs FLJ13868
EP73182 (+) Hs RPLP1
EP30058 (-) Hs MT-IE
EP73281 (+) Hs FANCL
EP74414 (+) Hs MAP2K1IP1
EP74071 (+) Hs RARS
EP73193 (+) Hs RGS4
EP73195 (+) Hs SFRS6
EP73197 (+) Hs MCP
EP73543 (+) Hs TM9SF2
EP73205 (+) Hs LPXN
EP73835 (+) Hs PECI
EP73211 (+) Hs DHRS6
EP74091 (+) Hs SERPINB1
EP74095 (+) Hs AGER
EP74099 (+) Hs CDK7 P2+
EP74445 (+) Hs CLPP
EP73225 (+) Hs RISC
EP74104 (+) Hs ACPP
EP73965 (+) Hs RNF7
EP73228 (+) Hs CCT2
EP16068 (+) Hs MHCII HLA DQ2b'
EP74112 (+) Hs MRPL45
EP74113 (+) Hs TARDBP
EP73919 (+) Hs BYSL
EP73138 (+) Hs B3GAT3
EP73582 (+) Hs EIF3S6
EP73869 (+) Hs SNW1
EP74469 (+) Hs SPARCL1 P1
EP73876 (+) Hs RAE1
EP74129 (+) Hs CHEK1
EP74139 (+) Hs ANGPTL3
EP73264 (+) Hs PROL4
EP73356 (+) Hs DKFZP566C243
EP73891 (+) Hs CMAS
EP74146 (+) Hs NIF3L1
EP73896 (+) Hs C3F
EP73272 (+) Hs SC4MOL
EP74499 (+) Hs PTDSS1
EP73395 (+) Hs OS
EP73283 (+) Hs ALDH1A1
EP73286 (+) Hs PIGT
EP74166 (+) Hs ARL1
EP74172 (+) Hs HNRPA1
EP74521 (+) Hs JUN
EP73298 (+) Hs ACAT2
EP73299 (+) Hs ACTR3
EP17094 (-) Hs TCR vb'8.2
EP73646 (+) Hs CPA2
EP73647 (+) Hs CPA3
EP73055 (+) Hs HNRPR
EP73308 (+) Hs FKBP1A
EP73313 (+) Hs LR8
EP74191 (+) Hs TSTA3 P1
EP74169 (+) Hs TCL1A
EP73316 (+) Hs LOC51064
EP73319 (+) Hs ZDHHC9
EP73321 (+) Hs LOC51125
EP73322 (+) Hs COPS4
EP74203 (+) Hs MRPS18B
EP73326 (+) Hs UBAP1
EP73418 (+) Hs THY1
EP73672 (+) Hs SNRPB
EP74013 (+) Hs VARS2
EP73956 (+) Hs MAGEE1
EP74310 (+) Hs RAD51
EP73238 (+) Hs FLJ10525
EP73966 (+) Hs RFP
EP74223 (+) Hs CRSP9
EP73344 (+) Hs ASML3B
EP74320 (+) Hs TM4SF1 P2+
EP73347 (+) Hs PTD004
EP73440 (+) Hs PPIE
EP73708 (+) Hs ACTA2
EP73352 (+) Hs C14ORF92
EP73106 (+) Hs ZFP36L1
EP74239 (+) Hs CREB3
EP73359 (+) Hs CBR1
EP74243 (+) Hs PDCD10
EP73365 (+) Hs D1S155E
EP74251 (+) Hs GARS P1
EP74253 (+) Hs SRP19
EP74252 (+) Hs GARS P2+
EP73124 (+) Hs ENSA
EP74600 (+) Hs STMN2
EP73466 (+) Hs SDCCAG10
EP74409 (+) Hs PSMC2
EP73384 (+) Hs CA12
EP74360 (+) Hs METTL1
EP74164 (+) Hs COX11
EP73390 (+) Hs MGST1
EP73509 (+) Hs CTSB
EP73394 (+) Hs LILRA2
EP74083 (+) Hs LAMR1
EP73399 (+) Hs TMP21
EP74283 (+) Hs COX5A
EP73152 (+) Hs NDUFA9
EP73153 (+) Hs NDUFS1
EP73405 (+) Hs PWP1
EP11068 (+) Hs histone H2A
EP73525 (+) Hs MEP1A
EP74098 (+) Hs CDK7 P1

EP11070 (+) Hs histone H2B
EP74562 (+) Hs PSMA1
EP25038 (+) Hs IFI 54K
EP73964 (+) Hs TBPL1
EP74392 (+) Hs RAB6A
EP73422 (+) Hs TXNIP
EP73423 (+) Hs CCT8
EP73424 (+) Hs PROL3
EP73426 (+) Hs PSMC4
EP73656 (+) Hs ATP5F1
EP73408 (+) Hs BTG1
EP73774 (+) Hs MLPH
EP73184 (+) Hs ENG
EP73303 (+) Hs AUP1
EP73783 (+) Hs ICAM1
EP73441 (+) Hs NDRG1
EP73442 (+) Hs SKB1
EP73443 (+) Hs CCT3
EP73650 (+) Hs FVT1
EP74331 (+) Hs MFGE8
EP73541 (+) Hs TRIP15
EP73448 (+) Hs AGR2
EP74486 (+) Hs DCN
EP73886 (+) Hs CRYL1
EP73461 (+) Hs ARPC2
EP73807 (+) Hs GSN
EP73579 (+) Hs TM4SF3
EP73465 (+) Hs LHFP
EP73467 (+) Hs LRRC17
EP41008 (+) Hs snRNP E

EP74449 (+) Hs DEAH
EP74162 (+) Hs CS
EP74165 (+) Hs KRT8
EP73573 (+) Hs SFRS10
EP73575 (+) Hs PIGH
EP73236 (+) Hs CDCA8
EP73832 (+) Hs GM2A
EP73245 (+) Hs NAGK
EP74493 (+) Hs RPS3A
EP74379 (+) Hs GMFG
EP73249 (+) Hs FLJ10509
EP73931 (+) Hs RPS25
EP73844 (+) Hs COL6A1
EP73618 (+) Hs RARRES2
EP73505 (+) Hs RBM3
EP73257 (+) Hs RPL23A
EP74192 (+) Hs TSTA3 P2+
EP73740 (+) Hs HSPD1
EP73852 (+) Hs ATP6V1D
EP73517 (+) Hs HADHSC
EP74399 (+) Hs PELO P2+
EP73270 (+) Hs RSU1
EP74451 (+) Hs NDUFA1
EP73523 (+) Hs IDH3A
EP74406 (+) Hs ERCC1
EP73957 (+) Hs APOH
EP74214 (+) Hs PSMD13
EP74155 (+) Hs MRPS25
EP73285 (+) Hs COX7A2
EP74418 (+) Hs AASDHPPT

EP74421 (+) Hs ATP6P1
EP73633 (+) Hs ADM
EP73884 (+) Hs LIPA
EP73107 (+) Hs IGFBP3
EP74431 (+) Hs SUCLG1
EP73782 (+) Hs HADHA
EP26031 (-) Hs c-abl 7 kb E1P2
EP73556 (+) Hs HIF1A
EP74244 (+) Hs MRPL9
EP14056 (+) Hs prolactin
EP73899 (+) Hs HSBP1
EP74186 (+) Hs TOR3A
EP73676 (+) Hs SRP9
EP73311 (+) Hs GORASP2
EP73562 (+) Hs CNN2
EP74601 (+) Hs PGRMC1
EP15029 (+) Hs b'-fibrinogen
EP73910 (+) Hs CLTA
EP73570 (+) Hs NDUFC2
EP74028 (+) Hs UBE2L6
EP73471 (+) Hs FRSB
EP73576 (+) Hs RAB5A
EP74458 (+) Hs IDH3G
EP74578 (+) Hs TYMS P2+
EP73920 (+) Hs MRPS22
EP73629 (+) Hs TAF15
EP74271 (+) Hs HLA DRB3
EP73827 (+) Hs DC6
EP73587 (+) Hs TCAP
EP73145 (+) Hs ST13

EP74470 (+) Hs SPARCL1 P2+
EP73930 (+) Hs IDH1
EP74473 (+) Hs NEDD8
EP74475 (+) Hs UROS
EP17051 (+) Hs apolipop. CII
EP73494 (+) Hs CCNB2
EP73038 (+) Hs DDX17
EP73715 (+) Hs PSMB7
EP73601 (+) Hs VDAC2
EP74485 (+) Hs IER3
EP73606 (+) Hs AKR1B1
EP73164 (+) Hs C9ORF19
EP73947 (+) Hs EIF3S6IP
EP74298 (+) Hs WBP11
EP26029 (+) Hs CRP
EP73854 (+) Hs HSPC177
EP73616 (+) Hs PHB
EP73959 (+) Hs HNRPK P1
EP73619 (+) Hs RBMX
EP73620 (+) Hs RCN1
EP30079 (+) Hs ATIII
EP73829 (+) Hs ALG2
EP73625 (+) Hs UGDH
EP74062 (+) Hs CNP
EP74514 (+) Hs TINF2
EP15024 (+) Hs histone H3.3
EP73382 (+) Hs PNLIP
EP74415 (+) Hs HTATIP
EP73534 (+) Hs CTSC
EP74327 (+) Hs MAGEA3

EP74558 (+) Hs SDCBP
EP11112 (-) Hs ferritin H
EP74420 (+) Hs GABARAPL2 P2+
EP73644 (+) Hs COPEB
EP73011 (+) Hs NTPBP
EP73660 (+) Hs PRKCSH
EP73649 (+) Hs DPT
EP73878 (+) Hs AP1S2
EP73017 (+) Hs UNRIP
EP73748 (+) Hs NDUFA2
EP73654 (+) Hs HMOX1
EP73784 (+) Hs MYL3
EP73750 (+) Hs NDUFA6
EP73863 (+) Hs PEPD
EP74349 (+) Hs TPMT
EP74543 (+) Hs NDUFS5
EP73219 (+) Hs RPL36A
EP74005 (+) Hs HAGH
EP74297 (+) Hs RPL30 P2+
EP74356 (+) Hs BAG1
EP73894 (+) Hs TRAP1
EP74358 (+) Hs ZWINT
EP57008 (+) Hs FBP
EP73569 (+) Hs NAP1L1
EP74012 (+) Hs DAP3
EP30051 (-) Hs stromelysin
EP74014 (+) Hs RBBP4
EP73677 (+) Hs STATH
EP73425 (+) Hs PC4
EP74457 (+) Hs TFAM

EP73967 (+) Hs NOLA3
EP73772 (+) Hs FLJ13188
EP73581 (+) Hs DNAJA1
EP74026 (+) Hs KYNU
EP74568 (+) Hs SNRPB2
EP73434 (+) Hs CTGF
EP73814 (+) Hs KIAA0174
EP25009 (-) Hs N-myc E1P2
EP25010 (+) Hs N-myc E2P3
EP73438 (+) Hs NFE2L2
EP27007 (+) Hs Bcl-2 E2P2
EP73493 (+) Hs SDHA
EP73252 (+) Hs TMEM30A
EP73700 (+) Hs PGM1
EP73498 (+) Hs PLS3
EP74586 (+) Hs SFRS8
EP35012 (+) Hs P-glycoprotein 1
EP74048 (+) Hs NIPSNAP1
EP74591 (+) Hs B3GNT6
EP73709 (+) Hs FKBP3
EP74102 (+) Hs TCEB1 P1
EP73460 (+) Hs ARPC5
EP73157 (+) Hs RPL19
EP73717 (+) Hs PSMC5
EP73159 (+) Hs FABP4
EP73724 (+) Hs SH3BGRL
EP73818 (+) Hs C14ORF100
EP74464 (+) Hs HSPA5
EP73172 (+) Hs FBL
EP73594 (+) Hs NRP1
EP74125 (+) Hs WRB P1
EP73329 (+) Hs C20ORF111
EP73558 (+) Hs ANXA11
EP73637 (+) Hs CRISP2
EP73603 (+) Hs P4HA2
EP73791 (+) Hs ZNF207
EP73882 (+) Hs DSIPI
EP73033 (+) Hs TGFBI
EP74093 (+) Hs TDO2
EP73913 (+) Hs DFFA
EP16046 (-) Hs a'-fetoprotein
EP73081 (+) Hs KIF22
EP74443 (+) Hs CLIC1 P1
EP74444 (+) Hs CLIC1 P2+
EP74381 (+) Hs BZW2 P1
EP73787 (+) Hs PNN
EP26001 (+) Hs TCR va' HD-Mar
EP73875 (+) Hs SMARCE1
EP74450 (+) Hs PSMD2
EP73766 (+) Hs TXNL2
EP25008 (+) Hs N-myc E1P1+
EP74110 (+) Hs UBE2G2
EP73098 (+) Hs UBL5
EP47002 (+) Hs a'_2 integrin
EP74511 (+) Hs HARS
EP73557 (+) Hs HRMT1L1
EP73912 (+) Hs CSRP2
EP73331 (+) Hs HSPC251
EP74463 (+) Hs CPE
EP73574 (+) Hs PET112L
EP73447 (+) Hs PAICS
EP73528 (+) Hs COPS3
EP74410 (+) Hs APPBP1
EP73893 (+) Hs PHGDH
EP73830 (+) Hs ASAH1
EP74138 (+) Hs MAEA
EP73698 (+) Hs PBP
EP73786 (+) Hs RPL27
EP73002 (+) Hs RPL7
EP73589 (+) Hs RTCD1
EP73841 (+) Hs NAPA
EP14060 (-) Hs IFN-a'5
EP73009 (+) Hs GJA1
EP74427 (+) Hs MRPS6
EP73980 (+) Hs MPHOSPH6
EP73013 (+) Hs CRYZ
EP74376 (+) Hs HEXB
EP73407 (+) Hs ARHGDIB
EP73018 (+) Hs NK4
EP74126 (+) Hs WRB P2+
EP73070 (+) Hs APOL3
EP73811 (+) Hs HPCL2
EP74525 (+) Hs C1QBP
EP73565 (+) Hs FDFT1
EP73567 (+) Hs GYG
EP73889 (+) Hs FLJ10298
EP73821 (+) Hs FLJ20422
EP73736 (+) Hs GNAI2
EP73801 (+) Hs RDHL
EP74476 (+) Hs STIP1
EP73156 (+) Hs RTN4
EP73941 (+) Hs ERH
EP73976 (+) Hs XBP1
EP73274 (+) Hs SSR4
EP74587 (+) Hs TTID
EP24039 (+) Hs vimentin
EP73737 (+) Hs H2AFZ
EP74397 (+) Hs FUCA1
EP73839 (+) Hs HNRPA3
EP74330 (+) Hs KDELR2
EP74533 (+) Hs MEST
EP74585 (+) Hs NDUFA5
EP73958 (+) Hs TIMM8A
EP73597 (+) Hs DEK
EP73847 (+) Hs RPS27
EP73604 (+) Hs LIPF
EP73453 (+) Hs HMGN4
EP73834 (+) Hs LYZ
EP73942 (+) Hs TSG101
EP73332 (+) Hs HPGD
EP73066 (+) Hs MMS19L
EP73093 (+) Hs CASQ2
EP73877 (+) Hs EIF3S3
EP73320 (+) Hs SBDS
EP73187 (+) Hs PON2
EP73173 (+) Hs SAP18
EP73973 (+) Hs NIT1
EP73078 (+) Hs ITM2C
EP74554 (+) Hs HNRPAB
EP73377 (+) Hs ARFIP2
EP73409 (+) Hs CST4
EP26025 (+) Hs MRP-8
EP74124 (+) Hs VBP1
EP74240 (+) Hs PTGES2
EP74234 (+) Hs RPL4
EP73432 (+) Hs BTF3
EP73333 (+) Hs LTA4H
EP74618 (+) Hs NFKBIB
EP73456 (+) Hs CHI3L1
EP73745 (+) Hs KPNB3
EP73951 (+) Hs SNX3 P1
EP28004 (-) Hs pepsinogen A_A
EP73151 (+) Hs C5orf13
EP74398 (+) Hs PELO P1
EP73028 (+) Hs FLJ14800
EP74238 (+) Hs RPL35A P2+
EP73764 (+) Hs RAD18
EP73911 (+) Hs ENC1
EP74142 (+) Hs RFX4
EP73907 (+) Hs RBM8A
EP25044 (-) Hs IL-5 (EDF/TRF)
EP73768 (+) Hs STMN1
EP73349 (+) Hs HSU53209
EP73036 (+) Hs SH3BGRL3
EP73815 (+) Hs HSPC142
EP73291 (+) Hs LOC51706
EP73141 (+) Hs MRPL44
EP73627 (+) Hs PTP4A1
EP73029 (+) Hs FLJ14904
EP73262 (+) Hs G3BP

EP74557 (+) Hs DECR1
EP73130 (+) Hs CRACC
EP73134 (+) Hs FABP5
EP73755 (+) Hs PLEK
EP74296 (+) Hs RPL30 P1
EP73642 (+) Hs BLMH
EP17093 (+) Hs TCR vb'8.1
EP73062 (+) Hs ACTR1A
EP74625 (+) Hs MRPS12
EP73397 (+) Hs RNP24
EP73480 (+) Hs MAT2A
EP74502 (+) Hs SNRPG
EP74365 (+) Hs POLR2I
EP73747 (+) Hs MIF
EP74178 (+) Hs RTN3
EP73793 (+) Hs RPL14
EP73147 (+) Hs MASP1
EP45003 (+) Hs endothelin-B rec.
EP73657 (+) Hs ATP6V1E1
EP73361 (+) Hs RER1
EP73691 (+) Hs LDHB
EP73628 (+) Hs PDHX
EP74075 (+) Hs WBSCR1
EP73542 (+) Hs TXNL
EP73497 (+) Hs GSTO1
EP74157 (+) Hs HIBCH
EP74523 (+) Hs DDX1
EP74480 (+) Hs CRI1
EP74526 (+) Hs PCNA
EP73502 (+) Hs TOM1
EP73696 (+) Hs OMG
EP74089 (+) Hs MRPL15
EP73887 (+) Hs RDH11
EP73945 (+) Hs S100A10
EP74163 (+) Hs NCBP2
EP73378 (+) Hs SF3B1
EP74468 (+) Hs HSD17B4
EP73045 (+) Hs CYP11A1
EP73268 (+) Hs UQCRB
EP73598 (+) Hs UNG
EP73536 (+) Hs UCHL1
EP73701 (+) Hs PIP
EP73300 (+) Hs DSTN
EP73960 (+) Hs HNRPK P2+
EP73024 (+) Hs DKFZp564K142
EP36018 (-) Hs nucleolin
EP73368 (+) Hs HSPCB
EP11135 (-) Hs IFN l'2h (-a'14)
EP73968 (+) Hs ING3
EP73318 (+) Hs ADIPOR1
EP73790 (+) Hs PSMD11
EP73481 (+) Hs CNN1
EP73302 (+) Hs PRDX4
EP73532 (+) Hs GATM
EP73459 (+) Hs CTNNA1
EP73032 (+) Hs PLP1
EP74046 (+) Hs UQCRC2
EP73648 (+) Hs CPB1
EP73622 (+) Hs SSR1
EP74460 (+) Hs TCTE1L
EP73444 (+) Hs TSC22
EP74319 (+) Hs TM4SF1 P1
EP73778 (+) Hs RAB7
EP73242 (+) Hs FLJ11160
EP73592 (+) Hs EIF3S1
EP73450 (+) Hs SLC35A1
EP73600 (+) Hs VASP
EP74422 (+) Hs NDUFB7
EP73346 (+) Hs DDX25
EP73853 (+) Hs CBX3
EP73232 (+) Hs RPL37
EP73209 (+) Hs DRG2
EP74408 (+) Hs TAF9
EP73087 (+) Hs DREV1
EP17079 (+) Hs IL1B
EP73367 (+) Hs ATF6
EP74306 (+) Hs TSFM
EP73788 (+) Hs PSMD1
EP74506 (+) Hs PSMD10
EP73810 (+) Hs COPE
EP11141 (+) Hs estrogen receptor
EP73342 (+) Hs p100
EP73813 (+) Hs M9
EP73888 (+) Hs C20ORF43
EP74387 (+) Hs TAX1BP1
EP73872 (+) Hs DC50
EP73176 (+) Hs SPUVE
EP73429 (+) Hs SLC2A1
EP73938 (+) Hs KNS2
EP73583 (+) Hs AMD1
EP74273 (+) Hs PMVK
EP73982 (+) Hs MTAP
EP74140 (+) Hs DNAJA2
EP73381 (+) Hs GNPAT
EP73984 (+) Hs CD37
EP74152 (+) Hs BET1
EP74595 (+) Hs SORD
EP74145 (+) Hs ANXA2
EP73388 (+) Hs BCAP31
EP17031 (-) Hs snRNA U1 (pHU1-1)
EP73174 (+) Hs PRNP
EP73290 (+) Hs LOC51705
EP73559 (+) Hs CTSL
EP26020 (+) Hs CA III muscle
EP73288 (+) Hs LOC51629
EP74309 (+) Hs RPS15A
EP73277 (+) Hs FLJ20424
EP74452 (+) Hs TBCC
EP73403 (+) Hs KIF3A
EP73354 (+) Hs KIAA0971

AGATATTT

EP73928 (+) Hs RBM14
EP73933 (+) Hs RAD51C
EP74613 (+) Hs CPSF5
EP74614 (+) Hs TSNAX
EP73940 (+) Hs NEU1
EP73942 (+) Hs TSG101
EP74622 (+) Hs PDHB
EP07113 (+) Hs g'-interferon
EP73949 (+) Hs HSPE1
EP74630 (+) Hs VAPA
EP14077 (+) Hs factor VIII
EP73706 (+) Hs BPGM
EP25044 (-) Hs IL-5 (EDF/TRF)
EP17080 (+) Hs IL-6 (BSF-2) P1
EP73961 (+) Hs EWSR1
EP73714 (+) Hs PSMB1
EP56004 (-) Hs glycophorin E
EP15056 (+) Hs E-resp. RNA pS2
EP25038 (+) Hs IFI 54K
EP27009 (+) Hs IFI 6-16
EP73972 (+) Hs HERPUD1
EP73973 (+) Hs NIT1
EP73980 (+) Hs MPHOSPH6
EP64001 (+) Hs rag-1
EP73985 (+) Hs CDC23
EP73986 (+) Hs CKMT1
EP73988 (+) Hs APEX2
EP73998 (+) Hs TAF7
EP74029 (+) Hs ETFA
EP74034 (+) Hs SKP1A
EP74035 (+) Hs PPP2R5C
EP74036 (+) Hs IMPA1
EP74038 (+) Hs NUP88
EP73791 (+) Hs ZNF207
EP73794 (+) Hs ATP5G3
EP74052 (+) Hs LIG1
EP74053 (+) Hs FAU P1
EP74055 (+) Hs APP
EP71003 (+) Hs RGS1
EP73807 (+) Hs GSN
EP30059 (-) Hs MT-IF
EP74076 (+) Hs RPS4Y
EP17091 (+) Hs SERPINA1 E1P1+
EP73829 (+) Hs ALG2
EP74091 (+) Hs SERPINB1
EP74104 (+) Hs ACPP
EP74105 (+) Hs PRKRA
EP16068 (+) Hs MHCII HLA DQ2b'
EP74133 (+) Hs UBE1
EP74134 (+) Hs UMPS
EP74141 (+) Hs TXN2
EP74143 (+) Hs TUBG1
EP73892 (+) Hs ZNF9
EP74174 (+) Hs PSMA4
EP72002 (+) Hs AGT P2
EP74181 (+) Hs ELOVL1
EP73931 (+) Hs RPS25
EP74186 (+) Hs TOR3A
EP73934 (+) Hs RABAC1
EP74191 (+) Hs TSTA3 P1
EP74192 (+) Hs TSTA3 P2+
EP73065 (+) Hs MATR3
EP74197 (+) Hs GPX2
EP74199 (+) Hs WBSCR20A
EP73699 (+) Hs PFN2
EP74203 (+) Hs MRPS18B
EP74204 (+) Hs SURF6
EP74205 (+) Hs RPS18
EP74206 (+) Hs EEF1B2 P1
EP74207 (+) Hs EEF1B2 P2+
EP73077 (+) Hs ALDH7A1
EP73078 (+) Hs ITM2C
EP74212 (+) Hs SNX17
EP74213 (+) Hs APLP2
EP73083 (+) Hs IL8
EP73084 (+) Hs CD63
EP73085 (+) Hs PMM2
EP74563 (+) Hs HPRT1 P1
EP73087 (+) Hs DREV1
EP74220 (+) Hs BCCIP
EP74223 (+) Hs CRSP9
EP73721 (+) Hs SAT
EP74224 (+) Hs RBX1
EP33035 (+) Hs CNTF
EP74227 (+) Hs LYPLA2
EP73726 (+) Hs SNAP25
EP74229 (+) Hs RNASE4
EP11107 (-) Hs ^Gg'-globin
EP74233 (+) Hs ANP32A
EP74237 (+) Hs RPL35A P1
EP74236 (+) Hs SSR3
EP74240 (+) Hs PTGES2
EP74241 (+) Hs CTSH
EP74242 (+) Hs MRPL50
EP74243 (+) Hs PDCD10
EP74248 (+) Hs HPD
EP74250 (+) Hs S100A1
EP74251 (+) Hs GARS P1
EP73133 (+) Hs RPS6
EP74265 (+) Hs PQBP1
EP74267 (+) Hs HSPA9B P1
EP74271 (+) Hs HLA DRB3
EP49001 (+) Hs histone H1t
EP30042 (+) Hs histone H1a
EP74285 (+) Hs DPH2L2
EP74286 (+) Hs CASP6
EP74288 (+) Hs CKN1
EP74292 (+) Hs MRPL13
EP73161 (+) Hs PIGPC1
EP11070 (+) Hs histone H2B
EP74300 (+) Hs GABARAPL1
EP74305 (+) Hs GPX3
EP74307 (+) Hs CKLFSF7
EP74309 (+) Hs RPS15A
EP74313 (+) Hs CSH1
EP73811 (+) Hs HPCL2
EP73185 (+) Hs CLU
EP74327 (+) Hs MAGEA3
EP30079 (+) Hs ATIII
EP73207 (+) Hs RPL22
EP73834 (+) Hs LYZ
EP74344 (+) Hs ARL4 P1
EP74345 (+) Hs ARL4 P2+
EP74346 (+) Hs HLA-E
EP74353 (+) Hs BUB3
EP74354 (+) Hs SERPINB6
EP74356 (+) Hs BAG1
EP74359 (+) Hs RBM4
EP74362 (+) Hs ETV5
EP24040 (+) Hs rp S14
EP74369 (+) Hs VRK1
EP74370 (+) Hs PDE6D
EP74373 (+) Hs PAPOLA P2+
EP74374 (+) Hs MRPL16
EP74375 (+) Hs SAS
EP74376 (+) Hs HEXB
EP74377 (+) Hs NDUFV2
EP74378 (+) Hs CCNH
EP73248 (+) Hs OSGEP
EP74381 (+) Hs BZW2 P1
EP74396 (+) Hs IMPA2
EP74398 (+) Hs PELO P1
EP74399 (+) Hs PELO P2+
EP73273 (+) Hs SC5DL
EP11083 (+) Hs keratin 67K
EP74157 (+) Hs HIBCH
EP24039 (+) Hs vimentin
EP74414 (+) Hs MAP2K1IP1
EP26019 (+) Hs neurofilament L

EP73287 (+) Hs ATP6V1H
EP25041 (+) Hs a'1(III) collagen
EP74423 (+) Hs DSCR5
EP74169 (+) Hs TCL1A
EP74429 (+) Hs AKR7A2
EP15029 (+) Hs b'-fibrinogen
EP11087 (+) Hs g'-fibrinogen
EP74455 (+) Hs RNPS1
EP47002 (+) Hs a'_2 integrin
EP73082 (+) Hs SPP1
EP74467 (+) Hs QDPR
EP74470 (+) Hs SPARCL1 P2+
EP73341 (+) Hs NAKAP95
EP73346 (+) Hs DDX25
EP74481 (+) Hs MRPL49
EP74238 (+) Hs RPL35A P2+
EP73381 (+) Hs GNPAT
EP74523 (+) Hs DDX1
EP73390 (+) Hs MGST1
EP74526 (+) Hs PCNA
EP74530 (+) Hs MRPL3 P1
EP74531 (+) Hs MRPL3 P2+
EP74535 (+) Hs PSMC6
EP74536 (+) Hs GCA
EP73402 (+) Hs JM5
EP74541 (+) Hs POLR2J
EP74544 (+) Hs TCEA2
EP74555 (+) Hs HLA-DPB1
EP74559 (+) Hs GYPC
EP73428 (+) Hs SARS

EP74564 (+) Hs HPRT1 P2+
EP74565 (+) Hs PROCR
EP11105 (-) Hs d'-globin
EP07077 (-) Hs ^Ag'-globin
EP11110 (-) Hs e'-globin
EP73448 (+) Hs AGR2
EP74585 (+) Hs NDUFA5
EP74587 (+) Hs TTID
EP57001 (-) Hs haptoglob Hp2
EP11111 (+) Hs haptoglob Hp1F
EP73552 (+) Hs FUS
EP25014 (+) Hs 2'5'-oligoA synt.
EP73277 (+) Hs FLJ20424
EP74609 (+) Hs EIF4B
EP73476 (+) Hs SR
EP64003 (+) Hs TdT
EP74367 (+) Hs DKC1
EP73237 (+) Hs RNPC4
EP73494 (+) Hs CCNB2
EP73499 (+) Hs CAPZB
EP73845 (+) Hs ATP6IP2
EP16050 (+) Hs HMG-CoA red.
EP11119 (+) Hs aldolase B
EP73862 (+) Hs TGOLN2
EP73962 (+) Hs RPL39L
EP16058 (+) Hs somatostatin I
EP74413 (+) Hs PCBP2
EP73534 (+) Hs CTSC
EP74176 (+) Hs RPS3
EP73551 (+) Hs PRDX6

EP73559 (+) Hs CTSL
EP73565 (+) Hs FDFT1
EP73566 (+) Hs GTF2F2
EP48003 (+) Hs collagenaseIV 72K
EP30051 (-) Hs stromelysin
EP30065 (+) Hs AMY1
EP73577 (+) Hs TGM2
EP73578 (+) Hs TM4SF2
EP73583 (+) Hs AMD1
EP73590 (+) Hs EIF3S7
EP73594 (+) Hs NRP1
EP73597 (+) Hs DEK
EP73604 (+) Hs LIPF
EP73611 (+) Hs MAD2L1
EP73367 (+) Hs ATF6
EP07105 (-) Hs POMC
(ACTH,b'LPH)
EP17067 (+) Hs glucagon
EP73496 (+) Hs VAMP3
EP73633 (+) Hs ADM
EP73636 (+) Hs TPM2
EP30074 (-) Hs FSH b'
EP73111 (+) Hs RNF2
EP14056 (+) Hs prolactin
EP73118 (+) Hs ADH5
EP74538 (+) Hs YWHAE
EP73467 (+) Hs LRRC17
EP28010 (+) Hs IGF II E2P2
EP17072 (-) Hs IGF II E3P3
EP28009 (-) Hs IGF II E4P4

EP73677 (+) Hs STATH
EP36006 (+) Hs AGT P3
EP73147 (+) Hs MASP1
EP73146 (+) Hs ATP5J
EP73439 (+) Hs NNMT
EP14061 (-) Hs IFN-a'16
EP14062 (-) Hs IFN-a'4b
EP11134 (-) Hs IFN l'2c1 (-a'17)
EP07111 (+) Hs LeIF-J (IFN-a'7)
EP17079 (+) Hs IL1B
EP73711 (+) Hs PPP1R7
EP73712 (+) Hs PRPS1
EP74598 (+) Hs PSMB4
EP30057 (-) Hs MT-IA
EP73719 (+) Hs RGS2
EP73187 (+) Hs PON2
EP73818 (+) Hs C14ORF100
EP73192 (+) Hs SERPIND1
EP26025 (+) Hs MRP-8
EP30056 (+) Hs OAT
EP73490 (+) Hs GBP2
EP73746 (+) Hs MCM3
EP57007 (+) Hs IL-8 receptor B
EP56002 (+) Hs glycophorin A
EP73765 (+) Hs PKIA
EP73770 (+) Hs SSB
EP73772 (+) Hs FLJ13188
EP11146 (+) Hs c-myc P1
EP74464 (+) Hs HSPA5
EP30017 (+) Hs PGK1

EP73005 (+) Hs RUVBL2
EP73259 (+) Hs DAB2
EP16042 (+) Hs ALB
EP73012 (+) Hs MGC3248
EP48006 (+) Hs IRF-2
EP59011 (+) Hs elk-1
EP74145 (+) Hs ANXA2
EP73556 (+) Hs HIF1A
EP73618 (+) Hs RARRES2
EP73285 (+) Hs COX7A2
EP73573 (+) Hs SFRS10
EP17092 (+) Hs SERPINA1 E2P2
EP73295 (+) Hs P17
EP73045 (+) Hs CYP11A1
EP73837 (+) Hs IDI1
EP73648 (+) Hs CPB1
EP74189 (+) Hs GSTZ1
EP73752 (+) Hs NDUFS4
EP73852 (+) Hs ATP6V1D
EP74295 (+) Hs CDC2
EP73856 (+) Hs CLN2
EP73858 (+) Hs ITGB2
EP36009 (+) Hs CEA
EP73866 (+) Hs SLC25A19
EP73620 (+) Hs RCN1
EP74562 (+) Hs PSMA1
EP73773 (+) Hs RNF25
EP73874 (+) Hs PSMA7
EP73876 (+) Hs RAE1
EP40003 (+) Hs DAF

EP73877 (+) Hs EIF3S3
EP73004 (+) Hs BZW1
EP73009 (+) Hs GJA1
EP73011 (+) Hs NTPBP
EP73019 (+) Hs SSBP1
EP73340 (+) Hs E2IG3
EP73022 (+) Hs CPSF3
EP73026 (+) Hs MGC11061
EP73653 (+) Hs GOT1
EP73033 (+) Hs TGFBI
EP73132 (+) Hs IL10RB
EP73042 (+) Hs ADH1B
EP73043 (+) Hs ADH1C
EP73920 (+) Hs MRPS22
EP73922 (+) Hs COG4
EP74276 (+) Hs TM7SF2
EP74567 (+) Hs POLD4
EP73062 (+) Hs ACTR1A
EP73692 (+) Hs LGALS3
EP73941 (+) Hs ERH
EP73066 (+) Hs MMS19L
EP74579 (+) Hs PEX11B
EP07112 (-) Hs b'-interferon
EP74294 (+) Hs BEX1
EP73073 (+) Hs SID6
EP73422 (+) Hs TXNIP
EP73081 (+) Hs KIF22
EP74595 (+) Hs SORD
EP73964 (+) Hs TBPL1
EP73965 (+) Hs RNF7

EP74119 (+) Hs HLA DQA1
EP73968 (+) Hs ING3
EP73435 (+) Hs MDH1
EP73098 (+) Hs UBL5
EP73440 (+) Hs PPIE
EP73442 (+) Hs SKB1
EP73103 (+) Hs CBARA1
EP73982 (+) Hs MTAP
EP74137 (+) Hs MRPS2
EP73452 (+) Hs DNAJB1
EP73989 (+) Hs ARL2
EP73116 (+) Hs TOPK
EP73117 (+) Hs AKR1C3
EP74343 (+) Hs MT1L
EP73121 (+) Hs ACTG2
EP73554 (+) Hs NDUFB9
EP45002 (+) Hs endothelin-A rec.
EP73560 (+) Hs B2M
EP74352 (+) Hs SIAT7D
EP74003 (+) Hs PRPF18
EP73684 (+) Hs EPHX2
EP73136 (+) Hs SLC26A3
EP73766 (+) Hs TXNL2
EP11145 (+) Hs FOS
EP73484 (+) Hs CTRB1
EP73488 (+) Hs CCNG1
EP74024 (+) Hs BFAR
EP74025 (+) Hs SDHD
EP74026 (+) Hs KYNU
EP11148 (+) Hs c-myc P2+

EP74122 (+) Hs NDUFB8
EP74121 (+) Hs NDUFB2
EP74031 (+) Hs GOT2
EP73156 (+) Hs RTN4
EP73500 (+) Hs CCNC
EP73593 (+) Hs SNAP23
EP73165 (+) Hs RAB18
EP74046 (+) Hs UQCRC2
EP73262 (+) Hs G3BP
EP73263 (+) Hs JWA
EP73264 (+) Hs PROL4
EP73800 (+) Hs PSMD14
EP73517 (+) Hs HADHSC
EP73175 (+) Hs PXMP3
EP73181 (+) Hs FXR1
EP74151 (+) Hs BCAS2
EP73810 (+) Hs COPE
EP74409 (+) Hs PSMC2
EP73427 (+) Hs REG1B
EP74298 (+) Hs WBP11
EP73533 (+) Hs ANXA1
EP73625 (+) Hs UGDH
EP73199 (+) Hs CTRC
EP73634 (+) Hs EIF5
EP73294 (+) Hs SLC38A2
EP73545 (+) Hs SURB7
EP73203 (+) Hs PSAP
EP73212 (+) Hs HNOEL
EP17050 (+) Hs vWf
EP73839 (+) Hs HNRPA3

EP73215 (+) Hs NFKBIA
EP73558 (+) Hs ANXA11
EP73217 (+) Hs PPIA
EP74096 (+) Hs PP
EP73317 (+) Hs RPS27L
EP74451 (+) Hs NDUFA1
EP74109 (+) Hs DYT1
EP73579 (+) Hs TM4SF3
EP73245 (+) Hs NAGK
EP74469 (+) Hs SPARCL1 P1
EP73639 (+) Hs TXNRD1
EP73363 (+) Hs SMT3H1
EP73252 (+) Hs TMEM30A
EP73002 (+) Hs RPL7
EP73599 (+) Hs UQCRC1
EP73601 (+) Hs VDAC2
EP73502 (+) Hs TOM1
EP73266 (+) Hs MBNL2
EP73267 (+) Hs PRDX3
EP73359 (+) Hs CBR1
EP73269 (+) Hs QPCT
EP73020 (+) Hs TOSO
EP74150 (+) Hs NDUFB4
EP74270 (+) Hs PRAME
EP74153 (+) Hs SKP2
EP73825 (+) Hs FLJ10374
EP73519 (+) Hs ATP5C1
EP73279 (+) Hs TAPBP
EP73029 (+) Hs FLJ14904
EP73281 (+) Hs FANCL

EP73283 (+) Hs ALDH1A1
EP74161 (+) Hs CCNB1
EP73376 (+) Hs MYCBP
EP74163 (+) Hs NCBP2
EP73377 (+) Hs ARFIP2
EP73289 (+) Hs YPEL5
EP73191 (+) Hs ATP6V1C1
EP74519 (+) Hs EIF3S5
EP74266 (+) Hs CDK5RAP1
EP73638 (+) Hs TXN
EP73690 (+) Hs UBE2N
EP73299 (+) Hs ACTR3
EP73301 (+) Hs AF1Q
EP73351 (+) Hs KIAA0102
EP73305 (+) Hs BPNT1
EP73652 (+) Hs GDI2
EP73061 (+) Hs ITM2B
EP73655 (+) Hs LGALS1
EP73316 (+) Hs LOC51064
EP73107 (+) Hs IGFBP3
EP74296 (+) Hs RPL30 P1
EP73324 (+) Hs VRK3
EP74549 (+) Hs NTS
EP73895 (+) Hs RNF13
EP73951 (+) Hs SNX3 P1
EP73956 (+) Hs MAGEE1
EP74558 (+) Hs SDCBP
EP73335 (+) Hs COX7C
EP41007 (+) Hs rp S17
EP74447 (+) Hs APLP1 P2+

EP74371 (+) Hs EIF3S8
EP73088 (+) Hs GNPI
EP11104 (+) Hs b'-globin
EP73342 (+) Hs p100
EP73344 (+) Hs ASML3B
EP73436 (+) Hs MFAP1
EP74426 (+) Hs VPS29
EP73347 (+) Hs PTD004
EP73783 (+) Hs ICAM1
EP73101 (+) Hs NP
EP73696 (+) Hs OMG
EP74503 (+) Hs HLA-DMA
EP74332 (+) Hs APOA1BP
EP73702 (+) Hs POU2F1
EP73360 (+) Hs CSNK1A1
EP74246 (+) Hs DDX19
EP73368 (+) Hs HSPCB
EP74252 (+) Hs GARS P2+
EP73372 (+) Hs HBP1
EP73373 (+) Hs HU
EP73375 (+) Hs LYPLA3
EP73723 (+) Hs SFRS3
EP73472 (+) Hs RBM5
EP73383 (+) Hs PPIB
EP74268 (+) Hs HSPA9B P2+
EP73138 (+) Hs B3GAT3
EP73957 (+) Hs APOH
EP73480 (+) Hs MAT2A
EP73391 (+) Hs TMSB10
EP73443 (+) Hs CCT3

EP73398 (+) Hs TDE1
EP31007 (+) Hs HMG-14
EP73745 (+) Hs KPNB3
EP73401 (+) Hs IDH3B
EP73495 (+) Hs SCYE1
EP74287 (+) Hs NACA
EP73751 (+) Hs NDUFB6
EP73409 (+) Hs CST4
EP74550 (+) Hs ATP5G1
EP73869 (+) Hs SNW1
EP56003 (-) Hs glycophorin B
EP73898 (+) Hs CSTF1
EP74391 (+) Hs MRPL51
EP73418 (+) Hs THY1
EP73320 (+) Hs SBDS
EP74569 (+) Hs LAMP2
EP73767 (+) Hs SOD2
EP74051 (+) Hs IFITM2
EP73425 (+) Hs PC4
EP73176 (+) Hs SPUVE
EP73214 (+) Hs LOC57019
EP74115 (+) Hs ZNF265
EP11127 (-) Hs LH b'
EP73447 (+) Hs PAICS
EP35012 (+) Hs P-glycoprotein 1
EP73784 (+) Hs MYL3
EP73444 (+) Hs TSC22
EP73689 (+) Hs UBE2L3
EP73054 (+) Hs HNRPH2
EP73350 (+) Hs HSU15552

EP73352 (+) Hs C14ORF92
EP74139 (+) Hs ANGPTL3
EP73891 (+) Hs CMAS
EP73509 (+) Hs CTSB
EP73460 (+) Hs ARPC5
EP74459 (+) Hs H2AFY
EP74349 (+) Hs TPMT
EP73471 (+) Hs FRSB
EP74158 (+) Hs RACGAP1
EP74048 (+) Hs NIPSNAP1
EP73228 (+) Hs CCT2
EP73232 (+) Hs RPL37
EP73270 (+) Hs RSU1
EP74170 (+) Hs PPAP2C
EP74114 (+) Hs MAD2L2
EP74173 (+) Hs ART3
EP74074 (+) Hs IFNGR1
EP74372 (+) Hs PAPOLA P1
EP11074 (+) Hs histone H4-A1
EP73050 (+) Hs CDC10
EP73498 (+) Hs PLS3
EP74497 (+) Hs GABARAP P2+
EP73823 (+) Hs ESRRBL1
EP73622 (+) Hs SSR1
EP73899 (+) Hs HSBP1
EP73753 (+) Hs NDUFS8
EP74488 (+) Hs ECHS1
EP73612 (+) Hs MDM4
EP74397 (+) Hs FUCA1
EP73072 (+) Hs WDR23

EP74493 (+) Hs RPS3A
EP73838 (+) Hs CDC42EP2
EP74495 (+) Hs ARAF1
EP73647 (+) Hs CPA3
EP73764 (+) Hs RAD18
EP73523 (+) Hs IDH3A
EP74155 (+) Hs MRPS25
EP74502 (+) Hs SNRPG
EP74410 (+) Hs APPBP1
EP73643 (+) Hs CNN3
EP74012 (+) Hs DAP3
EP73330 (+) Hs C14ORF129
EP73646 (+) Hs CPA2
EP73432 (+) Hs BTF3
EP73322 (+) Hs COPS4
EP74165 (+) Hs KRT8
EP07106 (-) Hs CG/LH/FSH/TSH a'
EP73787 (+) Hs PNN
EP11098 (-) Hs z'-globin
EP73757 (+) Hs HSD3B2
EP74432 (+) Hs MOCS2
EP15041 (+) Hs transferrin rec.
EP73608 (+) Hs AHCY
EP73991 (+) Hs MRPS31
EP73610 (+) Hs M6PR
EP73366 (+) Hs PARK7
EP73122 (+) Hs ADPRT
EP74417 (+) Hs SDHB
EP74254 (+) Hs RFXANK
EP17039 (-) Hs snRNA U3

EP73129 (+) Hs ECH1
EP16037 (+) Hs involucrin
EP73323 (+) Hs LOC51185
EP73229 (+) Hs CG005
EP73575 (+) Hs PIGH
EP74033 (+) Hs HADH2
EP74577 (+) Hs TYMS P1
EP73970 (+) Hs CUL1
EP73331 (+) Hs HSPC251
EP73582 (+) Hs EIF3S6
EP74551 (+) Hs HNMT
EP73926 (+) Hs CLDN5
EP74468 (+) Hs HSD17B4
EP73145 (+) Hs ST13
EP74160 (+) Hs YARS
EP73691 (+) Hs LDHB
EP73641 (+) Hs CXCL6
EP73343 (+) Hs TM4SF13
EP14058 (-) Hs IFN-a'13
EP73318 (+) Hs ADIPOR1
EP74600 (+) Hs STMN2
EP73357 (+) Hs MGP
EP73698 (+) Hs PBP
EP74442 (+) Hs RPL15
EP14063 (+) Hs IL-1a'
EP73950 (+) Hs ABCE1
EP74475 (+) Hs UROS
EP74596 (+) Hs ATP5G2
EP74418 (+) Hs AASDHPPT
EP73174 (+) Hs PRNP

EP73619 (+) Hs RBMX
EP73937 (+) Hs RFC3
EP30054 (+) Hs arginase liver
EP68001 (-) Hs CA2
EP73407 (+) Hs ARHGDIB
EP73541 (+) Hs TRIP15
EP73865 (+) Hs DDX24
EP73183 (+) Hs RPS4X
EP74366 (+) Hs SYAP1
EP73952 (+) Hs SNX3 P2+
EP74483 (+) Hs TMSB4X
EP73379 (+) Hs SPON2
EP73380 (+) Hs PPP2R1A
EP73382 (+) Hs PNLIP
EP73923 (+) Hs SYNJ2BP
EP74326 (+) Hs FBXO22
EP73637 (+) Hs CRISP2
EP74329 (+) Hs GLTSCR2
EP73830 (+) Hs ASAH1
EP73235 (+) Hs SLC31A2
EP73315 (+) Hs OVCOV1
EP73255 (+) Hs BAP29
EP74388 (+) Hs MAT2B
EP73470 (+) Hs PPIF
EP74110 (+) Hs UBE2G2
EP73705 (+) Hs PRKAR1A
EP74592 (+) Hs FADD
EP73656 (+) Hs ATP5F1
EP73801 (+) Hs RDHL
EP74463 (+) Hs CPE
EP73106 (+) Hs ZFP36L1
EP72001 (+) Hs AGT P1
EP73758 (+) Hs HDAC2
EP16046 (-) Hs a'-fetoprotein
EP74357 (+) Hs MRPL37
EP73516 (+) Hs H3F3B
EP74590 (+) Hs WDR1
EP74557 (+) Hs DECR1
EP74416 (+) Hs JTB
EP73776 (+) Hs PCNT1
EP33011 (+) Hs DES
EP73707 (+) Hs C1S
EP73028 (+) Hs FLJ14800
EP30003 (+) Hs c-N-ras
EP73241 (+) Hs HIMAP4
EP73586 (+) Hs APOL1
EP73701 (+) Hs PIP
EP74446 (+) Hs APLP1 P1
EP17084 (+) Hs IgE rec. FC_e'R
EP74496 (+) Hs GABARAP P1
EP73944 (+) Hs IGFBP7
EP74382 (+) Hs BZW2 P2+
EP74599 (+) Hs CD79B
EP74433 (+) Hs GNB3
EP73761 (+) Hs TGFB1I1
EP73749 (+) Hs NDUFA4
EP73814 (+) Hs KIAA0174
EP73535 (+) Hs TTC4
EP73703 (+) Hs PPP2CA
EP73795 (+) Hs SP100
EP74486 (+) Hs DCN
EP73164 (+) Hs C9ORF19
EP73804 (+) Hs CCT6B
EP73298 (+) Hs ACAT2
EP74415 (+) Hs HTATIP
EP73754 (+) Hs ORC4L
EP57002 (-) Hs haptoglob HpR
EP73511 (+) Hs ALDOC
EP73614 (+) Hs MTIF2
EP74312 (+) Hs MRPL10
EP73616 (+) Hs PHB
EP28005 (+) Hs pepsinogen C
EP73720 (+) Hs RNF4
EP74297 (+) Hs RPL30 P2+
EP74460 (+) Hs TCTE1L
EP74117 (+) Hs APCS
EP73353 (+) Hs STAF65
EP74582 (+) Hs S100B
EP73826 (+) Hs ACTR10
EP73205 (+) Hs LPXN
EP73031 (+) Hs C14ORF2
EP74588 (+) Hs NFYB
EP73196 (+) Hs EBNA1BP2
EP17081 (+) Hs IL-6 (BSF-2) P2+
EP45003 (+) Hs endothelin-B rec.
EP74172 (+) Hs HNRPA1
EP73312 (+) Hs SDCBP2
EP74299 (+) Hs SELENBP1
EP73189 (+) Hs SERPINB2
EP73512 (+) Hs CALM3
EP74401 (+) Hs MRPS23
EP60004 (+) Hs link
EP26038 (-) Hs IL-4 (BSF-1)
EP74457 (+) Hs TFAM
EP74257 (+) Hs PHF7
EP73548 (+) Hs ITM2A
EP73842 (+) Hs TES
EP74330 (+) Hs KDELR2
EP73451 (+) Hs NPC2
EP74427 (+) Hs MRPS6
EP73735 (+) Hs GLUL
EP73629 (+) Hs TAF15
EP73131 (+) Hs TFG
EP74128 (+) Hs BZRP
EP73354 (+) Hs KIAA0971
EP73395 (+) Hs OS
EP74152 (+) Hs BET1
EP73424 (+) Hs PROL3
EP73035 (+) Hs PCCA
EP30039 (-) Hs CRF
EP73929 (+) Hs MRPL42
EP73080 (+) Hs FLJ12525
EP74054 (+) Hs FAU P2+
EP74321 (+) Hs POLR2K
EP73675 (+) Hs SRP14
EP74505 (+) Hs HMGN3
EP74177 (+) Hs PTTG1
EP73240 (+) Hs FLJ11000
EP26029 (+) Hs CRP
EP73901 (+) Hs PPP1R12A
EP73687 (+) Hs UBE2D1
EP74573 (+) Hs DAD1
EP73790 (+) Hs PSMD11
EP73037 (+) Hs HBXIP
EP74578 (+) Hs TYMS P2+
EP73907 (+) Hs RBM8A
EP73596 (+) Hs SLC7A7
EP74506 (+) Hs PSMD10
EP73492 (+) Hs ILK
EP73911 (+) Hs ENC1
EP73134 (+) Hs FABP5
EP73724 (+) Hs SH3BGRL
EP73162 (+) Hs LCN7
EP17096 (+) Hs CD3 (T3) g'
EP73222 (+) Hs LOC57862
EP73224 (+) Hs MASA
EP15024 (+) Hs histone H3.3
EP73024 (+) Hs DKFZp564K142
EP74603 (+) Hs ATP6V1G1
EP73854 (+) Hs HSPC177
EP73536 (+) Hs UCHL1
EP73310 (+) Hs COX7B
EP73588 (+) Hs STC2
EP74188 (+) Hs NUDC
EP73674 (+) Hs SRI
EP73649 (+) Hs DPT
EP73421 (+) Hs USP16
EP07056 (+) Hs DHFR
EP73332 (+) Hs HPGD
EP73233 (+) Hs RPL39

EP73193 (+) Hs RGS4

EP73994 (+) Hs WBSCR22

EP73455 (+) Hs NOL5A

EP74083 (+) Hs LAMR1

EP74234 (+) Hs RPL4

EP74597 (+) Hs ARF6

EP73268 (+) Hs UQCRB

EP73478 (+) Hs SLC35A2

EP74328 (+) Hs UQCRFS1

EP74089 (+) Hs MRPL15

EP73276 (+) Hs FLJ20420

EP73023 (+) Hs MGC2668

EP73913 (+) Hs DFFA

EP26028 (+) Hs apolipop. B

EP73438 (+) Hs NFE2L2

EP74453 (+) Hs ARHG

EP74456 (+) Hs ILF2

EP73859 (+) Hs LPL

AGTAATTT
EP73143 (+) Hs NQO1
EP73930 (+) Hs IDH1
EP74612 (+) Hs PMAIP1
EP74614 (+) Hs TSNAX
EP74613 (+) Hs CPSF5
EP73937 (+) Hs RFC3
EP14058 (-) Hs IFN-a'13
EP14059 (-) Hs IFN-a'6
EP74604 (+) Hs HLA-DPA1
EP73941 (+) Hs ERH
EP73946 (+) Hs HEXA
EP73699 (+) Hs PFN2
EP73954 (+) Hs WDR13
EP73952 (+) Hs SNX3 P2+
EP39004 (+) Hs CSRP1
EP56002 (+) Hs glycophorin A
EP73963 (+) Hs CAMLG
EP56004 (-) Hs glycophorin E
EP73965 (+) Hs RNF7
EP73718 (+) Hs PSMD7
EP27009 (+) Hs IFI 6-16
EP73723 (+) Hs SFRS3
EP28011 (+) Hs[GdX]
EP73980 (+) Hs MPHOSPH6
EP64001 (+) Hs rag-1
EP26025 (+) Hs MRP-8
EP73986 (+) Hs CKMT1
EP73744 (+) Hs KPNB2
EP16071 (+) Hs[ERV3]
EP73749 (+) Hs NDUFA4
EP73998 (+) Hs TAF7
EP73755 (+) Hs PLEK
EP74002 (+) Hs STK25 P3+
EP73760 (+) Hs AHSA1
EP57008 (+) Hs FBP
EP11148 (+) Hs c-myc P2+
EP74029 (+) Hs ETFA
EP74032 (+) Hs MSN
EP74033 (+) Hs HADH2
EP74035 (+) Hs PPP2R5C
EP74034 (+) Hs SKP1A
EP73790 (+) Hs PSMD11
EP74055 (+) Hs APP
EP74070 (+) Hs COX6A1
EP74071 (+) Hs RARS
EP74083 (+) Hs LAMR1
EP74090 (+) Hs SLC1A5
EP74095 (+) Hs AGER
EP74101 (+) Hs RPL24
EP74105 (+) Hs PRKRA
EP74096 (+) Hs PP
EP07121 (+) Hs MHCII HLA-DRA
EP74116 (+) Hs SAA4
EP74118 (+) Hs KRT5
EP74122 (+) Hs NDUFB8
EP74124 (+) Hs VBP1
EP74127 (+) Hs COPS5
EP74135 (+) Hs YME1L1
EP74134 (+) Hs UMPS
EP74152 (+) Hs BET1
EP73025 (+) Hs MGC2714
EP74158 (+) Hs RACGAP1
EP74160 (+) Hs YARS
EP74162 (+) Hs CS
EP74166 (+) Hs ARL1
EP74164 (+) Hs COX11
EP73918 (+) Hs ATP6V0D1
EP74173 (+) Hs ART3
EP74176 (+) Hs RPS3
EP74177 (+) Hs PTTG1
EP74183 (+) Hs NUBP2
EP74187 (+) Hs PLGL
EP73933 (+) Hs RAD51C
EP73061 (+) Hs ITM2B
EP74174 (+) Hs PSMA4
EP74193 (+) Hs APG12L
EP74197 (+) Hs GPX2
EP73944 (+) Hs IGFBP7
EP73070 (+) Hs APOL3
EP74549 (+) Hs NTS
EP74206 (+) Hs EEF1B2 P1
EP74207 (+) Hs EEF1B2 P2+
EP73080 (+) Hs FLJ12525
EP74214 (+) Hs PSMD13
EP73960 (+) Hs HNRPK P2+
EP73083 (+) Hs IL8
EP73085 (+) Hs PMM2
EP73082 (+) Hs SPP1
EP74218 (+) Hs HNRPH3
EP74223 (+) Hs CRSP9
EP74228 (+) Hs STARD7
EP74229 (+) Hs RNASE4
EP11107 (-) Hs ^Gg'-globin
EP73731 (+) Hs CALM2
EP74239 (+) Hs CREB3
EP74236 (+) Hs SSR3
EP17031 (-) Hs snRNA U1 (pHU1-1)
EP74250 (+) Hs S100A1
EP74251 (+) Hs GARS P1
EP74252 (+) Hs GARS P2+
EP30056 (+) Hs OAT
EP49001 (+) Hs histone H1t
EP73773 (+) Hs RNF25
EP74285 (+) Hs DPH2L2
EP74289 (+) Hs SEPP1
EP73161 (+) Hs PIGPC1
EP74297 (+) Hs RPL30 P2+
EP74295 (+) Hs CDC2
EP73796 (+) Hs SRP54
EP74300 (+) Hs GABARAPL1
EP74305 (+) Hs GPX3
EP73176 (+) Hs SPUVE
EP74061 (+) Hs RNASEH1
EP11073 (+) Hs histone H3b
EP74319 (+) Hs TM4SF1 P1
EP74069 (+) Hs SCARB1
EP74326 (+) Hs FBXO22
EP73191 (+) Hs ATP6V1C1
EP74327 (+) Hs MAGEA3
EP73193 (+) Hs RGS4
EP33023 (+) Hs TNP1
EP74339 (+) Hs USP5
EP74086 (+) Hs MRPL20
EP74341 (+) Hs UP
EP74345 (+) Hs ARL4 P2+
EP74349 (+) Hs TPMT
EP74441 (+) Hs ETFB
EP74097 (+) Hs CAPZA1
EP74354 (+) Hs SERPINB6
EP74355 (+) Hs MRPS36
EP73223 (+) Hs SQRDL
EP74353 (+) Hs BUB3
EP74373 (+) Hs PAPOLA P2+
EP74372 (+) Hs PAPOLA P1
EP74392 (+) Hs RAB6A
EP73009 (+) Hs GJA1
EP73263 (+) Hs JWA
EP74395 (+) Hs MCM2
EP73275 (+) Hs FLJ20320
EP73276 (+) Hs FLJ20420
EP74157 (+) Hs HIBCH
EP24039 (+) Hs vimentin
EP74419 (+) Hs GABARAPL2 P1
EP73287 (+) Hs ATP6V1H
EP74422 (+) Hs NDUFB7
EP74420 (+) Hs GABARAPL2 P2+
EP25041 (+) Hs a'1(III) collagen
EP24025 (-) Hs a'2(I) collagen
EP60004 (+) Hs link

EP74433 (+) Hs GNB3
EP73306 (+) Hs CARP
EP74440 (+) Hs SFRS2
EP73316 (+) Hs LOC51064
EP11087 (+) Hs g'-fibrinogen
EP73072 (+) Hs WDR23
EP74455 (+) Hs RNPS1
EP74460 (+) Hs TCTE1L
EP74465 (+) Hs DAZAP2
EP74475 (+) Hs UROS
EP73343 (+) Hs TM4SF13
EP74476 (+) Hs STIP1
EP73690 (+) Hs UBE2N
EP74486 (+) Hs DCN
EP73353 (+) Hs STAF65
EP73354 (+) Hs KIAA0971
EP16042 (+) Hs ALB
EP16046 (-) Hs a'-fetoprotein
EP74496 (+) Hs GABARAP P1
EP74497 (+) Hs GABARAP P2+
EP74242 (+) Hs MRPL50
EP74503 (+) Hs HLA-DMA
EP74504 (+) Hs ARF5
EP17080 (+) Hs IL-6 (BSF-2) P1
EP74253 (+) Hs SRP19
EP73374 (+) Hs FTSJ1
EP74518 (+) Hs ATP6V1F
EP73133 (+) Hs RPS6
EP74521 (+) Hs JUN
EP17092 (+) Hs SERPINA1 E2P2

EP74530 (+) Hs MRPL3 P1
EP74531 (+) Hs MRPL3 P2+
EP73147 (+) Hs MASP1
EP74523 (+) Hs DDX1
EP74539 (+) Hs H2AFO
EP74542 (+) Hs SNRPA1
EP73222 (+) Hs LOC57862
EP74548 (+) Hs DARS
EP73419 (+) Hs TIMM23
EP74563 (+) Hs HPRT1 P1
EP11146 (+) Hs c-myc P1
EP74570 (+) Hs TRA1
EP74572 (+) Hs PSMB3
EP74575 (+) Hs CCT6A P2+
EP74578 (+) Hs TYMS P2+
EP74133 (+) Hs UBE1
EP74582 (+) Hs S100B
EP74586 (+) Hs SFRS8
EP74587 (+) Hs TTID
EP74588 (+) Hs NFYB
EP73462 (+) Hs ARPC1B
EP30054 (+) Hs arginase liver
EP73489 (+) Hs FARSL
EP74630 (+) Hs VAPA
EP14077 (+) Hs factor VIII
EP47012 (+) Hs[NOS2] iNOS
EP74386 (+) Hs CLDN4
EP30014 (+) Hs G6PD
EP73537 (+) Hs UXT
EP74426 (+) Hs VPS29

EP73548 (+) Hs ITM2A
EP73550 (+) Hs EI24
EP73013 (+) Hs CRYZ
EP73302 (+) Hs PRDX4
EP73018 (+) Hs NK4
EP73554 (+) Hs NDUFB9
EP15054 (+) Hs complement f. B
EP73560 (+) Hs B2M
EP73561 (+) Hs CLK1
EP73564 (+) Hs STOM
EP73582 (+) Hs EIF3S6
EP73733 (+) Hs CCND3
EP73586 (+) Hs APOL1
EP73686 (+) Hs TPT1
EP73594 (+) Hs NRP1
EP73402 (+) Hs JM5
EP70008 (+) Hs ACP2
EP14063 (+) Hs IL-1a'
EP73706 (+) Hs BPGM
EP73614 (+) Hs MTIF2
EP73365 (+) Hs D1S155E
EP73959 (+) Hs HNRPK P1
EP73770 (+) Hs SSB
EP74505 (+) Hs HMGN3
EP73622 (+) Hs SSR1
EP07105 (-) Hs POMC
(ACTH,b'LPH)
EP73626 (+) Hs ZNF134
EP17067 (+) Hs glucagon
EP73630 (+) Hs TPST2

EP73633 (+) Hs ADM
EP73444 (+) Hs TSC22
EP73634 (+) Hs EIF5
EP73640 (+) Hs UBE2A
EP73647 (+) Hs CPA3
EP73655 (+) Hs LGALS1
EP73124 (+) Hs ENSA
EP73658 (+) Hs ADFP
EP73676 (+) Hs SRP9
EP73677 (+) Hs STATH
EP74562 (+) Hs PSMA1
EP73154 (+) Hs RPL10
EP14060 (-) Hs IFN-a'5
EP73694 (+) Hs NASP
EP74579 (+) Hs PEX11B
EP74580 (+) Hs GCHFR
EP74569 (+) Hs LAMP2
EP11110 (-) Hs e'-globin
EP74584 (+) Hs CKS2
EP73159 (+) Hs FABP4
EP73702 (+) Hs POU2F1
EP73707 (+) Hs C1S
EP73710 (+) Hs PPM1G
EP73803 (+) Hs TUBB5
EP74595 (+) Hs SORD
EP73579 (+) Hs TM4SF3
EP74404 (+) Hs U2AF1
EP73719 (+) Hs RGS2
EP73720 (+) Hs RNF4
EP73724 (+) Hs SH3BGRL

EP17088 (+) Hs hsp 70K
EP73196 (+) Hs EBNA1BP2
EP17091 (+) Hs SERPINA1 E1P1+
EP73543 (+) Hs TM9SF2
EP15044 (+) Hs EGF receptor P2
EP74087 (+) Hs GOLPH3
EP73748 (+) Hs NDUFA2
EP73497 (+) Hs GSTO1
EP73751 (+) Hs NDUFB6
EP45003 (+) Hs endothelin-B rec.
EP74098 (+) Hs CDK7 P1
EP73119 (+) Hs TF
EP15029 (+) Hs b'-fibrinogen
EP73573 (+) Hs SFRS10
EP73233 (+) Hs RPL39
EP73767 (+) Hs SOD2
EP73239 (+) Hs C20ORF44
EP73527 (+) Hs LGMN
EP73242 (+) Hs FLJ11160
EP73246 (+) Hs C6ORF37
EP73785 (+) Hs MKI67IP
EP73252 (+) Hs TMEM30A
EP73542 (+) Hs TXNL
EP73262 (+) Hs G3BP
EP59011 (+) Hs elk-1
EP73268 (+) Hs UQCRB
EP73799 (+) Hs CNIH
EP47009 (+) Hs DDH hepatic
EP71003 (+) Hs RGS1
EP73174 (+) Hs PRNP

EP73618 (+) Hs RARRES2
EP37011 (-) Hs MT-IG
EP07055 (+) Hs MT2A
EP73816 (+) Hs ABS
EP73567 (+) Hs GYG
EP73286 (+) Hs PIGT
EP73831 (+) Hs GLA
EP74333 (+) Hs COX8
EP73835 (+) Hs PECI
EP73839 (+) Hs HNRPA3
EP73840 (+) Hs LGALS4
EP73842 (+) Hs TES
EP73593 (+) Hs SNAP23
EP73312 (+) Hs SDCBP2
EP17081 (+) Hs IL-6 (BSF-2) P2+
EP25039 (-) Hs b'2-AR
EP73317 (+) Hs RPS27L
EP74546 (+) Hs UCP2
EP73664 (+) Hs PSMA2
EP73323 (+) Hs LOC51185
EP73073 (+) Hs SID6
EP14076 (+) Hs CD74
EP73214 (+) Hs LOC57019
EP73443 (+) Hs CCT3
EP73075 (+) Hs DUSP11
EP73335 (+) Hs COX7C
EP73232 (+) Hs RPL37
EP73875 (+) Hs SMARCE1
EP74568 (+) Hs SNRPB2
EP73002 (+) Hs RPL7
EP73346 (+) Hs DDX25
EP73004 (+) Hs BZW1
EP73345 (+) Hs ALP
EP73349 (+) Hs HSU53209
EP73012 (+) Hs MGC3248
EP73352 (+) Hs C14ORF92
EP74526 (+) Hs PCNA
EP73131 (+) Hs TFG
EP73017 (+) Hs UNRIP
EP73360 (+) Hs CSNK1A1
EP73019 (+) Hs SSBP1
EP74246 (+) Hs DDX19
EP73117 (+) Hs AKR1C3
EP73026 (+) Hs MGC11061
EP73028 (+) Hs FLJ14800
EP73029 (+) Hs FLJ14904
EP73772 (+) Hs FLJ13188
EP73037 (+) Hs HBXIP
EP73383 (+) Hs PPIB
EP73042 (+) Hs ADH1B
EP73921 (+) Hs ATP5L
EP73047 (+) Hs RPS5
EP73390 (+) Hs MGST1
EP73596 (+) Hs SLC7A7
EP73485 (+) Hs BNIP3L
EP73052 (+) Hs LTBR
EP74328 (+) Hs UQCRFS1
EP73931 (+) Hs RPS25
EP73687 (+) Hs UBE2D1
EP73400 (+) Hs TOMM34
EP74283 (+) Hs COX5A
EP73058 (+) Hs GPX1
EP74402 (+) Hs SNURF
EP73294 (+) Hs SLC38A2
EP73523 (+) Hs IDH3A
EP74099 (+) Hs CDK7 P2+
EP73814 (+) Hs KIAA0174
EP73871 (+) Hs FLJ12895
EP73416 (+) Hs SPTLC1
EP73074 (+) Hs DDX5
EP73853 (+) Hs CBX3
EP73508 (+) Hs ANXA4
EP73771 (+) Hs SNX6
EP73964 (+) Hs TBPL1
EP73180 (+) Hs FLJ11730
EP73865 (+) Hs DDX24
EP74430 (+) Hs CRIP2
EP73435 (+) Hs MDH1
EP73437 (+) Hs MYL9
EP73211 (+) Hs DHRS6
EP73438 (+) Hs NFE2L2
EP73215 (+) Hs NFKBIA
EP73878 (+) Hs AP1S2
EP73441 (+) Hs NDRG1
EP73652 (+) Hs GDI2
EP73978 (+) Hs NUTF2
EP73448 (+) Hs AGR2
EP73500 (+) Hs CCNC
EP73112 (+) Hs NONO
EP73113 (+) Hs MKLN1
EP73115 (+) Hs FLJ10769
EP73116 (+) Hs TOPK
EP73460 (+) Hs ARPC5
EP73995 (+) Hs EIF1AY
EP73900 (+) Hs MAP1LC3B
EP73812 (+) Hs HSPBP1
EP73367 (+) Hs ATF6
EP74003 (+) Hs PRPF18
EP73472 (+) Hs RBM5
EP73134 (+) Hs FABP5
EP74449 (+) Hs DEAH
EP73476 (+) Hs SR
EP73036 (+) Hs SH3BGRL3
EP73141 (+) Hs MRPL44
EP74005 (+) Hs HAGH
EP30065 (+) Hs AMY1
EP73260 (+) Hs COX4I1
EP73486 (+) Hs BST2
EP73578 (+) Hs TM4SF2
EP74023 (+) Hs LUC7L
EP73152 (+) Hs NDUFA9
EP73496 (+) Hs VAMP3
EP73590 (+) Hs EIF3S7
EP73675 (+) Hs SRP14
EP74037 (+) Hs AK2
EP73846 (+) Hs NOLA2
EP74388 (+) Hs MAT2B
EP73750 (+) Hs NDUFA6
EP73259 (+) Hs DAB2
EP73795 (+) Hs SP100
EP74138 (+) Hs MAEA
EP74049 (+) Hs EIF4A2
EP73169 (+) Hs D21S2056E
EP73495 (+) Hs SCYE1
EP74517 (+) Hs DHX38
EP74145 (+) Hs ANXA2
EP11068 (+) Hs histone H2A
EP73280 (+) Hs FLJ10276
EP73305 (+) Hs BPNT1
EP73432 (+) Hs BTF3
EP73533 (+) Hs ANXA1
EP73310 (+) Hs COX7B
EP74076 (+) Hs RPS4Y
EP73653 (+) Hs GOT1
EP73747 (+) Hs MIF
EP74474 (+) Hs ADSL
EP73827 (+) Hs DC6
EP74581 (+) Hs GNG11
EP73891 (+) Hs CMAS
EP73256 (+) Hs HMT
EP73209 (+) Hs DRG2
EP74089 (+) Hs MRPL15
EP73765 (+) Hs PKIA
EP74558 (+) Hs SDCBP
EP73272 (+) Hs SC4MOL
EP73910 (+) Hs CLTA
EP73854 (+) Hs HSPC177
EP73571 (+) Hs NDUFS2
EP74577 (+) Hs TYMS P1
EP36010 (-) Hs NCA

EP73678 (+) Hs THBS1	EP74335 (+) Hs SIRT2 P2+	EP73173 (+) Hs SAP18	EP73493 (+) Hs SDHA	EP73818 (+) Hs C14ORF100
EP73245 (+) Hs NAGK	EP73054 (+) Hs HNRPH2	EP74235 (+) Hs CD24	EP73494 (+) Hs CCNB2	EP73088 (+) Hs GNPI
EP73864 (+) Hs CYP3A5	EP73649 (+) Hs DPT	EP73447 (+) Hs PAICS	EP73405 (+) Hs PWP1	EP74384 (+) Hs NDUFS3
EP74564 (+) Hs HPRT1 P2+	EP73218 (+) Hs PPP2R4	EP74437 (+) Hs SFRS1	EP73190 (+) Hs SERPINA3	EP56003 (-) Hs glycophorin B
EP74243 (+) Hs PDCD10	EP73423 (+) Hs CCT8	EP42001 (+) Hs BCKDHA	EP73205 (+) Hs LPXN	EP73685 (+) Hs TM4SF6
EP73253 (+) Hs FLJ11305	EP73521 (+) Hs SCARB2	EP73703 (+) Hs PPP2CA	EP73570 (+) Hs NDUFC2	EP73705 (+) Hs PRKAR1A
EP73120 (+) Hs CHIT1	EP73407 (+) Hs ARHGDIB	EP73452 (+) Hs DNAJB1	EP73387 (+) Hs SFRS7	EP73638 (+) Hs TXN
EP73888 (+) Hs C20ORF43	EP73659 (+) Hs ENO3	EP11111 (+) Hs haptoglob Hp1F	EP73623 (+) Hs TARS	EP73281 (+) Hs FANCL
EP74483 (+) Hs TMSB4X	EP73066 (+) Hs MMS19L	EP74051 (+) Hs IFITM2	EP73804 (+) Hs CCT6B	EP74597 (+) Hs ARF6
EP73942 (+) Hs TSG101	EP73610 (+) Hs M6PR	EP73456 (+) Hs CHI3L1	EP74557 (+) Hs DECR1	EP73860 (+) Hs CPR2
EP73399 (+) Hs TMP21	EP74414 (+) Hs MAP2K1IP1	EP73873 (+) Hs FAM14A	EP74344 (+) Hs ARL4 P1	EP73569 (+) Hs NAP1L1
EP74493 (+) Hs RPS3A	EP73758 (+) Hs HDAC2	EP74334 (+) Hs SIRT2 P1	EP73022 (+) Hs CPSF3	EP73126 (+) Hs GLO1
EP73609 (+) Hs EIF4E	EP73111 (+) Hs RNF2	EP73187 (+) Hs PON2	EP74129 (+) Hs CHEK1	EP74030 (+) Hs EIF2B2
EP73269 (+) Hs QPCT	EP74009 (+) Hs VAT1	EP73122 (+) Hs ADPRT	EP74321 (+) Hs POLR2K	EP73076 (+) Hs LOC81501
EP74268 (+) Hs HSPA9B P2+	EP73468 (+) Hs P5	EP73920 (+) Hs MRPS22	EP73644 (+) Hs COPEB	EP73477 (+) Hs TAF11
EP73273 (+) Hs SC5DL	EP73328 (+) Hs HSPC148	EP73877 (+) Hs EIF3S3	EP73764 (+) Hs RAD18	EP73708 (+) Hs ACTA2
EP74306 (+) Hs TSFM	EP73332 (+) Hs HPGD	EP73616 (+) Hs PHB	EP74453 (+) Hs ARHG	EP73412 (+) Hs POLD2
EP73175 (+) Hs PXMP3	EP73424 (+) Hs PROL3	EP31007 (+) Hs HMG-14	EP73612 (+) Hs MDM4	EP73202 (+) Hs IK
EP74506 (+) Hs PSMD10	EP74368 (+) Hs FEN1	EP74491 (+) Hs ATP5O	EP15024 (+) Hs histone H3.3	EP74429 (+) Hs AKR7A2
EP73420 (+) Hs TIMM44	EP73394 (+) Hs LILRA2	EP30042 (+) Hs histone H1a	EP73212 (+) Hs HNOEL	EP74535 (+) Hs PSMC6
EP73031 (+) Hs C14ORF2	EP74418 (+) Hs AASDHPPT	EP73471 (+) Hs FRSB	EP73670 (+) Hs SEMG2	EP73484 (+) Hs CTRB1
EP73629 (+) Hs TAF15	EP73341 (+) Hs NAKAP95	EP73545 (+) Hs SURB7	EP74115 (+) Hs ZNF265	EP73156 (+) Hs RTN4
EP74320 (+) Hs TM4SF1 P2+	EP73823 (+) Hs ESRRBL1	EP73234 (+) Hs DLD	EP73475 (+) Hs SGK	EP73043 (+) Hs ADH1C
EP73912 (+) Hs CSRP2	EP73688 (+) Hs UBE2D3	EP73138 (+) Hs B3GAT3	EP74537 (+) Hs POLR2C	EP73436 (+) Hs MFAP1
EP73838 (+) Hs CDC42EP2	EP73482 (+) Hs CSE1L	EP74130 (+) Hs SGT	EP73624 (+) Hs TCF12	EP74438 (+) Hs VDAC3
EP73862 (+) Hs TGOLN2	EP74574 (+) Hs CCT6A P1	EP73439 (+) Hs NNMT	EP41008 (+) Hs snRNP E	EP73534 (+) Hs CTSC
EP74325 (+) Hs DUT	EP73488 (+) Hs CCNG1	EP14056 (+) Hs prolactin	EP73451 (+) Hs NPC2	EP73095 (+) Hs MEP50
EP74571 (+) Hs PRIM1	EP73440 (+) Hs PPIE	EP73469 (+) Hs PBEF	EP73197 (+) Hs MCP	EP27010 (+) Hs L-myc
EP73300 (+) Hs DSTN	EP23004 (+) Hs ALDH_2 mit	EP73602 (+) Hs YWHAB	EP74139 (+) Hs ANGPTL3	EP73490 (+) Hs GBP2
EP31009 (+) Hs HMG-17	EP73782 (+) Hs HADHA	EP73298 (+) Hs ACAT2	EP73576 (+) Hs RAB5A	EP73801 (+) Hs RDHL

EP73950 (+) Hs ABCE1
EP73290 (+) Hs LOC51705
EP74431 (+) Hs SUCLG1
EP73304 (+) Hs BLCAP
EP73896 (+) Hs C3F
EP73763 (+) Hs TACSTD1
EP73575 (+) Hs PIGH
EP73943 (+) Hs SMC2L1
EP74495 (+) Hs ARAF1
EP73461 (+) Hs ARPC2
EP73913 (+) Hs DFFA
EP73465 (+) Hs LHFP
EP73907 (+) Hs RBM8A
EP73264 (+) Hs PROL4
EP73348 (+) Hs HSPC051
EP74459 (+) Hs H2AFY
EP73639 (+) Hs TXNRD1
EP73908 (+) Hs GTF2H4
EP73375 (+) Hs LYPLA3
EP73541 (+) Hs TRIP15
EP73237 (+) Hs RNPC4
EP73826 (+) Hs ACTR10
EP73989 (+) Hs ARL2
EP74413 (+) Hs PCBP2
EP74389 (+) Hs BAT1
EP73050 (+) Hs CDC10
EP45002 (+) Hs endothelin-A rec.
EP74376 (+) Hs HEXB
EP30071 (+) Hs TSH b'
EP74369 (+) Hs VRK1

EP73800 (+) Hs PSMD14
EP73010 (+) Hs CD9
EP73709 (+) Hs FKBP3
EP73498 (+) Hs PLS3
EP73951 (+) Hs SNX3 P1
EP74450 (+) Hs PSMD2
EP73595 (+) Hs EIF2S2
EP16068 (+) Hs MHCII HLA DQ2b'
EP73340 (+) Hs E2IG3
EP14030 (+) Hs a'-tubulin ba'1
EP73453 (+) Hs HMGN4
EP17089 (+) Hs HSPA8
EP35012 (+) Hs P-glycoprotein 1
EP30051 (-) Hs stromelysin
EP73177 (+) Hs MKKS
EP73148 (+) Hs INDO
EP74257 (+) Hs PHF7
EP74593 (+) Hs SNRPD1
EP74084 (+) Hs UFD1L
EP74267 (+) Hs HSPA9B P1
EP74427 (+) Hs MRPS6
EP73991 (+) Hs MRPS31
EP74565 (+) Hs PROCR
EP73032 (+) Hs PLP1
EP74536 (+) Hs GCA
EP73532 (+) Hs GATM
EP73589 (+) Hs RTCD1
EP73376 (+) Hs MYCBP
EP73051 (+) Hs PDLIM1
EP73874 (+) Hs PSMA7

EP74140 (+) Hs DNAJA2
EP74296 (+) Hs RPL30 P1
EP73339 (+) Hs SMP1
